# *Lutzomyia longipalpis* salivary proteins elicit human innate and adaptive immune responses detrimental to *Leishmania* parasites

**DOI:** 10.1101/2025.02.25.640210

**Published:** 2025-03-04

**Authors:** Maha Abdeladhim, Clarissa Teixeira, Roseanne Ressner, Kelly Hummer, Ranadhir Dey, Regis Gomes, Waldionê de Castro, Fernanda Fortes de Araujo, George W. Turiansky, Eva Iniguez, Claudio Meneses, Fabiano Oliveira, Naomi Aronson, Joshua R. Lacsina, Jesus G. Valenzuela, Shaden Kamhawi

**Affiliations:** 1 Vector Molecular Biology Section, Laboratory of Malaria and Vector Research, National Institute of Allergy and Infectious Diseases, National Institutes of Health, Rockville, Maryland, United States.; 2 Laboratory of Immunoparasitology, Department of Biotechnology, Oswaldo Cruz Foundation, Eusébio, Ceará, Brazil.; 3 Walter Reed National Military Medical Center, Bethesda, Maryland, United States.; 4 Center for Infectious Disease Research, Walter Reed Army Institute of Research, Silver Spring, Maryland, United States.; 5 Infectious Diseases Division, Department of Medicine, Uniformed Services University of the Health Sciences, Bethesda, Maryland, United States.; 6 Henry M Jackson Foundation for the Advancement of Military Medicine, Bethesda, Maryland, United States.; 7 Division of Emerging and Transfusion Transmitted Diseases, Office of Blood Research and Review, Center for Biologics Evaluation and Research, Food and Drug Administration, Silver Spring, Maryland, United States.

## Abstract

*Leishmania* parasites are transmitted via the bite of infected sand flies, whose saliva modulates host immune responses to promote *Leishmania* infection, especially in unexposed individuals. For humans in endemic areas, the immune consequences of chronic exposure to sand fly saliva remain poorly understood. We performed a human challenge study with *Lutzomyia longipalpis*, the primary vector of visceral leishmaniasis in the Americas. Fifteen healthy volunteers were exposed multiple times to uninfected *Lu. longipalpis* bites over the course of a year. PBMCs collected after several exposures were stimulated *ex vivo* by recombinant *Lu. longipalpis* salivary proteins to measure cytokine responses. Two salivary proteins, LJM19 and LJL143, elicited T_H_1-polarized cytokine responses, but with high co-expression of the T_H_2 cytokine IL-13. LJM19 also induced higher levels of IL-6 and IL-7, while both LJM19 and LJL143 induced the innate cytokines IL-1β and IFN-α. Importantly, T_H_1 polarization induced by LJM19 or LJL143 in PBMCs correlated with enhanced killing of *Leishmania* in co-cultured macrophages. Skin biopsies from two volunteers revealed bite site infiltration with CD4^−^CD8^-^ T cells. Our data suggest that sand fly exposed individuals demonstrate robust innate and adaptive cellular immune responses to vector salivary proteins that can be co-opted to protect humans against *Leishmania* infection.

## INTRODUCTION

Leishmaniasis is a neglected vector-borne disease transmitted by the bite of phlebotomine sand flies infected with *Leishmania* protozoan parasites. The disease has three primary clinical manifestations which correlates with the parasite species: cutaneous leishmaniasis (CL), the most common; mucosal leishmaniasis (ML), highly disfiguring and resistant to treatment; and visceral leishmaniasis (VL), the most severe and fatal if untreated (1). Although *Leishmania* infections cause over 700,000 new cases worldwide every year (1), there is no vaccine available for human leishmaniasis.

Sand fly saliva plays a critical role in *Leishmania* transmission. When female sand flies bite their host to take a blood meal, they egest a complex inoculum of vector-derived factors, including salivary proteins, that trigger an immune response at the bite site favoring parasite establishment (reviewed in (2)). In *Leishmania*-endemic areas, humans are constantly exposed to sand fly salivary proteins as foreign antigens that are deposited in the skin with each sand fly bite, however there are few clinical studies that characterize the consequences of this chronic antigenic exposure on the human immune system. Remarkably, the cytokine response of PBMCs to sand fly saliva can be recalled anywhere from one to ten years after a person’s most recent sand fly exposure (3, 4) and depends on the duration and intensity of exposure (5). Similarly in the skin, the ability to elicit a delayed type hypersensitivity (DTH) response to sand fly bites can persist for decades in humans with ongoing sand fly exposure (6), demonstrating that human immunity to sand fly saliva is long-lived. Interestingly, in patients with CL, the cellular and antibody responses to certain sand fly salivary proteins correlate strongly with CL lesion size and whether disease is localized or disseminated (7, 8). These studies suggest that chronic exposure to sand fly saliva exerts profound and durable effects on human immunity, including immune responses to *Leishmania*. Thus, a more detailed understanding of human immunity to sand fly saliva is critical for developing novel approaches to disrupt the early steps of *Leishmania* infection.

In preclinical models, several studies have demonstrated that the host immune response to sand fly salivary proteins can be co-opted to facilitate protection against *Leishmania* infection. Using sand fly salivary proteins as *Leishmania* vaccine candidates originated from the observation that pre-exposure to uninfected sand fly bites protects mice against experimental challenge with sand fly-transmitted *Leishmania* (9). This protection was associated with a DTH response at the bite site and upregulation of interferon gamma (IFN-γ), a key T_H_1 cytokine that promotes the killing of *Leishmania* parasites within infected macrophages (reviewed in (10)). Multiple salivary protein vaccine candidates that elicit robust protective responses against *Leishmania* have been identified in rodents (reviewed in (11, 12)), dogs (13), and non-human primates (14), however the immunogenicity of these salivary proteins in a human immune context remains largely unexplored.

While most investigations have focused on adaptive immunity to sand fly salivary proteins, comparatively little is known about how these proteins modulate innate immune responses. Certain sand fly salivary proteins exhibit adjuvant-like activity and can induce robust immunity against *Leishmania*, even in the absence of exogenous adjuvant or parasite-derived antigens (14–16). This suggests that these salivary proteins are “self-adjuvanted,” acting both as immunogens themselves and potentiating the immunogenicity of other vaccine antigens (17, 18), however the innate immune signals that mediate these adjuvant effects remain to be elucidated.

Human immunity to sand fly saliva has been investigated via human challenge studies in which healthy human volunteers are exposed to the bites of uninfected sand flies. Although only a few such human challenge studies have been performed, they demonstrate that most sand fly exposed volunteers develop a skin DTH response to sand fly bites and a mixed cellular T_H_1/T_H_2 cytokine response when their PBMCs are stimulated with salivary gland extract (SGE) *ex vivo* (4, 6, 19). A recent human challenge study extended these findings from whole saliva to identify individual salivary proteins from the Afro-Asian sand fly *Phlebotomus duboscqi* that induce strong IFN-γ or IL-10 responses in PBMCs (19).

In this clinical study, we investigated human cellular immunity to the saliva of the sand fly *Lutzomyia longipalpis*, the principal vector of visceral leishmaniasis in the Americas. We identified two sand fly salivary proteins that elicit robust innate and T_H_1-polarized cytokine responses in human immune cells and enhance killing of *Leishmania* parasites *ex vivo*. Importantly, these cellular immune responses were characterized by challenging individuals with uninfected *Lu. longipalpis* bites multiple times over the course of nearly a year, thereby simulating more closely the chronic immune exposure to sand fly saliva that occurs in *Leishmania*-endemic populations.

## METHODS

### Sex as a Biological Variable

Our study investigated male and female participants, but sex was not specifically tested as a biological variable due to the limited sample size (*n* = 15).

### Study Population

The screening cohort and enrollment criteria have been published previously for a related study on human immunity to the bites of *Phlebotomus duboscqi*, a sand fly vector of cutaneous leishmaniasis predominantly found in Africa (19), however there was no overlap in the enrollment cohort of the prior study and the current one. In this single site study conducted at Walter Reed Army Medical Center (WRAMC), 68 healthy individuals were screened and 15 were enrolled in the *Lu. longipalpis* study cohort. Study participants provided demographic information using options defined by the investigator. A medical history and physical examination were obtained and included a review of allergies and travel history. The inclusion criteria were healthy military healthcare beneficiaries between 18 and 50 years old who were willing to remain in the local area for the next 12 months and participate in all study procedures. Exclusion criteria included a history of travel for more than 30 consecutive days to a geographic area where *Lutzomyia longipalpis* is present, positivity by screening ELISA to IgG that bind *Lu. longipalpis* salivary gland extract (SGE), elevated serum IgE >144 kU/L, pregnancy, history of chronic medical illness, large skin reactions to insect bites, problems with prior phlebotomy, or use of medications that may interfere with immune responses.

### Human Controlled Exposure to Uninfected Laboratory-Reared *Lutzomyia longipalpis*

The colony of *Lu. longipalpis* sand flies used for this study was originally field collected in Jacobina, Brazil. For the clinical study, *Lu. longipalpis* were reared in a pathogen-free insectary at the Laboratory of Malaria and Vector Research (LMVR), NIAID and were maintained as a closed colony. For each participant, 10 female *Lu. longipalpis* sand flies were starved overnight, loaded into a feeding chamber, then transported to WRAMC on the day of the exposure. Each feeding chamber is composed of a sealed Plexiglass capsule with a fine mesh surface (Precision Plastics, Inc.). The feeding chamber was secured to the upper arm of each participant with the mesh side contacting the skin, allowing the sand flies to feed through the mesh. Each sand fly exposure lasted 20 minutes, during which the feeding chamber was lightly covered with a dark fabric to promote a dark feeding environment. Areas of skin with tattoos were avoided for feeding sites. At the end of each exposure, all sand flies were accounted for and examined by microscopy to assess the number of flies that had taken a blood meal.

**Figure 1.**
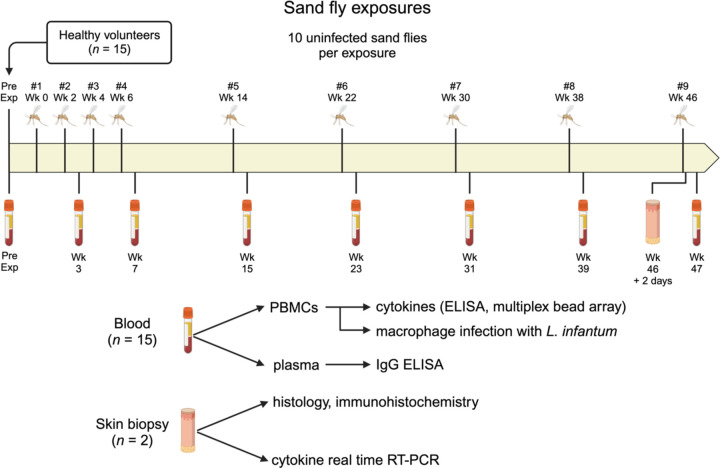
Schematic of clinical study. Healthy volunteers (*n* = 15) were exposed to the bites of uninfected *Lutzomyia longipalpis* sand flies once every two weeks for exposures #1 through #4, then once every eight weeks for exposures #5 to #9. Blood was collected pre-exposure (Pre Exp) and ~1 week after exposures #2, #4, and #5 to #9, then separated into peripheral blood mononuclear cells (PBMCs) and plasma to perform the indicated assays. Skin punch biopsies were obtained from two participants 48 hours after exposure #9, with one biopsy from a sand fly bite site and one biopsy from the contralateral arm for each participant. Created in BioRender.com.

Bite site skin reactions were observed by study physicians for 10 minutes immediately following the end of the sand fly exposure. Any participants with skin reactions deemed to be large or potentially allergic were observed for a longer period. Bite site photographs were taken and the physical appearance of the bite site rash and any associated symptoms were recorded. Participants were counseled to refrain from using antihistamines or topical steroids for bite site symptoms until consultation with study physicians. Sand fly exposures were performed once every two weeks for exposures #1 through #4, then once every eight weeks for exposures #5 through #9 (Figure 1). Feeding sites were alternated between different arms on consecutive visits. Blood was collected from participants 7 ± 3 days following each sand fly exposure, at which time the bite site was reassessed for delayed skin reactions. Participants who completed all nine planned sand fly exposures could elect to participate in an optional sub-study investigating skin immunity to *Lu. longipalpis* bites. In this sub-study, a 3 mm skin punch biopsy (Miltex sterile skin punch biopsy tool) was collected from a sand fly bite site 48 hours after the final sand fly exposure (#9), along with a 2 mm biopsy of skin from the contralateral arm as a negative control.

### Preparation of *Lu. longipalpis* Salivary Gland Extract

Salivary gland extract (SGE) was prepared by dissection of salivary glands from seven day old, laboratory-reared, uninfected adult female *Lu. longipalpis*. Glands were homogenized by ultrasonication with a Branson Sonifier 450 for three 30 second cycles then clarified by centrifugation at 10,000 *xg* for 3 min at 4 °C. Supernatant extracts were collected and stored at −80 °C until use.

### Blood Collection and Storage

Blood was collected from each study participant in heparinized Vacutainer tubes (BD Diagnostics). Collections were performed after exposures #2, #4, and #5 through #9 (Figure 1). Peripheral blood mononuclear cells (PBMCs) were isolated by density gradient centrifugation using a Ficoll-Paque PLUS solution (GE Healthcare). Plasma supernatants were collected and stored at −80 °C. PBMCs were counted, resuspended in fetal bovine serum (FBS) with 10% dimethyl sulfoxide (DMSO) solution, and transferred to cryovials which were slowly cooled to −80 °C overnight in a Mr. Frosty freezing container (Thermo Fisher Scientific) then transferred to liquid nitrogen.

To obtain pre-exposure negative controls, PBMCs were collected from each study participant by apheresis prior to sand fly exposure. However, initial tests of these apheresed PBMCs demonstrated that they were broadly reactive to the majority of recombinant sand fly salivary proteins tested (data not shown), indicating that the apheresis procedure had caused non-specific activation of these PBMC batches. Thus, we elected to use PBMCs collected from healthy volunteers at the NIH Blood Bank as *Lu. longipalpis* unexposed negative controls in our stimulation assays (Figure 3 and Figure 6). Blood from each donor was screened by ELISA to verify they were negative for IgG against *Lu. longipalpis* SGE. All post-exposure blood was collected via conventional venous phlebotomy.

### Measurement of Anti-SGE IgG

To measure specific anti-*Lu. longipalpis* SGE IgG by enzyme-linked immunosorbent assay (ELISA), 96-well high-binding microtiter plates (Thermo Fisher Scientific) were coated with 50 μl of SGE (1 pair/ml) in 0.1 M carbonate-bicarbonate buffer overnight at 4 °C. The wells were washed with wash buffer (Tris buffered saline, TBS + 0.05% Tween 20) then blocked with TBS + 4% bovine serum albumin (BSA) for 1 hour at room temperature (RT). Wells were washed three times with wash buffer, then 50 μl of 1:100 diluted sample plasma was added to each well and incubated for 1 hour at 37 °C. Antibody-antigen complexes were detected using alkaline phosphatase-conjugated goat anti-human IgG (H+L) antibody (Sigma, MO) diluted to 1:5000 for 1 hour at RT then measured using a nitrophenyl phosphate liquid substrate kit according to the manufacturer’s instructions (Sigma). Absorbance was measured at 405 nm using a VersaMax Microplate Reader (Molecular Devices).

### Cloning and Expression of *Lu. longipalpis* Salivary Proteins

Recombinant salivary proteins from *Lu. longipalpis* were produced as previously described (19). Briefly, DNA of the salivary proteins was amplified by PCR with addition of a hexa-histidine tag, cloned into the VR2001-TOPO vector (20), then expressed in HEK-293F cells at the Protein Expression Laboratory at NCI Frederick (Frederick, Maryland). At 72 hours, supernatants were collected, concentrated, then purified by HPLC using NiSO_4_-charged columns and imidazole gradient elution. Specific fractions were selected based on molecular weight, concentrated, then purified further via molecular sieving. Fractions of interest were collected, pooled, and protein concentration was measured by spectrophotometry. Endotoxin levels were <20 EU/ml for all proteins.

### Peripheral Blood Mononuclear Cell Culture and Stimulation

For stimulation assays, cryopreserved PBMCs were thawed quickly at 37 °C, diluted in RPMI 1640 medium, then pelleted by centrifugation for 10 min at 350 *xg* at RT. PBMCs were cultured in RPMI 1640 medium supplemented with 10% AB human serum (Sigma), 1% sodium pyruvate, 1% non-essential amino acids, 1% HEPES buffer, 50 µM beta-mercaptoethanol, and 40 mg/ml penicillin/streptomycin in a 5% CO_2_ humidified atmosphere at 37 °C overnight. Cells were counted and viability was assessed using Trypan blue (Hyclone, Thermo Fisher Scientific). PBMCs were then cultured in 96-well plates in cell culture medium at 1 × 10^6^ cells/ml in a final volume of 200 μl per well and incubated with medium alone, SGE (0.5 pairs/ml), 10 μg/ml recombinant *Lu. longipalpis* salivary proteins, or 2.5 μg/ml concavalin A (ConA). Cell supernatants were collected after 96 hours, clarified by centrifugation for 10 min at 350 *xg* at 4 °C, then stored at −80 °C until further use.

Within each assay, PBMCs from each individual were taken from a single time point, though the time point used varied between individuals, depending on the availability of PBMCs. The range of time points used for each assay are detailed in the sections that follow.

### Interferon Gamma ELISA

ELISA was performed on thawed supernatants of stimulated PBMCs using the anti-human interferon gamma ELISA kit (BD Biosciences) according to the manufacturer’s instructions. The results were interpolated from a standard curve using recombinant IFN-γ and expressed as the concentration of IFN-γ (pg/ml) minus the amount of background IFN-γ secreted from cells treated with media alone. PBMCs from exposure #2 or #4 were used for the initial screening of recombinant salivary proteins (Figure 3A), while PBMCs from exposure #4 through #9 were used for validation of the two candidate proteins found to induce the highest levels of IFN-γ (Figure 3, C and D).

### Cytokine Multiplex Bead Array

Cytokine concentrations in the supernatants of PBMCs from exposure #4 through #9 were measured using the Cytokine Human Magnetic 25-Plex Panel (Invitrogen) according to the manufacturer’s instructions.

### PBMC-Macrophage Co-Culture *Leishmania* Killing Assay

Human macrophages were derived from PBMCs of exposures #7 through #9 in a subset of study participants (*n* = 4) who completed all nine *Lu. longipalpis* exposures or from NIH blood bank volunteers (*n* = 4). Two technical replicates were performed on PBMCs from each participant, for a total of 8 samples per sand fly exposure group. For differentiation into macrophages, PBMCs were plated in a 16-well chamber slide at 3 × 10^5^ cells per well in the presence of complete RPMI and 20 ng/ml of GM-CSF for 1 hour at 37 °C with 5% CO_2_. Non-adherent cells were removed, then media was replaced with complete RPMI + 20 ng/ml GM-CSF. On day 5, matched donor PBMCs were thawed for use in the autologous killing assay and cultured in suspension in RPMI. On day 6, the cultured macrophages were infected with stationary phase *L*. *infantum* at a parasite:macrophage ratio of 5:1, then incubated for 8 hours at 37 °C with 5% CO_2_. Non-internalized parasites were removed by washing. Autologous PBMCs were treated with media, phytohemagglutinin (PHA) (6.25 µg/ml), or salivary protein (10 µg/ml) for 4 hours at 37 °C with 5% CO_2_, then added to the *Leishmania*-infected macrophages at 5 × 10^5^ PBMCs per well. After 5 days of co-culture, cells were fixed, stained with Giemsa, and assessed by light microscopy. Approximately 300 macrophages were counted per well and evaluated for the number of intracellular *Leishmania* amastigotes.

### Skin Histology and Immunohistochemistry

Forty-eight hours after their final exposure (#9) to *Lu. longipalpis*, two study participants (#1 and #13) consented to have a 3 mm skin punch biopsy taken at a bite site from the most recent exposure and a 2 mm punch biopsy from normal appearing skin on the contralateral arm as a negative control. Each biopsy was bisected, with one half stored in 10% buffered formalin for histology and the other half stored in RNAlater (Ambion) for quantitative (real time) RT-PCR.

Embedding of the biopsies as formalin-fixed paraffin-embedded (FFPE) blocks and histological staining was performed by Histoserv (Germantown, Maryland). Five micron-thick tissue sections were stained with hematoxylin and eosin and evaluated by light microscopy. For immunohistochemistry (IHC), primary antibodies against the following targets were used at the dilutions listed: CD3 at 1:100 (Dako #A0452), CD4 at 1:80 (Dako #M7310), CD8 at 1:75 (Dako #M7103) at 1:75, CD20 at 1:300 (Dako #M0755), CD68 at 1:100 (Dako #M0814), and myeloperoxidase (MPO) at 1:400 (Dako#A0398). For secondary antibodies, biotinylated anti-mouse IgG (1:500) was used to detect primary antibodies binding CD4, CD8, CD20, CD68, and biotinylated anti-rabbit IgG (1:500) was used to detect anti-CD3. Streptavidin-horseradish peroxidase was used to visualize the protein targets. Slide photography was performed using an Olympus DP73 camera microscope BX51 with Cellsens Dimension Olympus software. The percentage of cells positive for each IHC marker was quantitated with ImageJ software.

### Quantitative RT-PCR of Skin Cytokines

For each biopsy portion stored in RNAlater, RNA was extracted using the RNeasy Fibrous Tissue Mini Kit (Qiagen) and treated with DNase I to remove contaminating genomic DNA. Total RNA (100 ng) was used for cDNA synthesis using the qScript cDNA Supermix (Quanta Biosciences). Absence of genomic DNA contamination was verified by PCR of total RNA. Relative quantification of IFN-γ, IL-12, IL-4, IL-5, and IL-13 was performed in a LightCycler 480 (Roche Applied Science) using the Universal ProbeLibrary system (Roche). Primers and probes were designed using ProbeFinder software (v 2.45, Roche). Relative quantification of target genes normalized to 18S rRNA was performed using the LightCycler 480 software. Cytokine gene expression from the bite site biopsies was then normalized to the skin biopsy from the contralateral arm.

### Statistics

Statistical analyses were performed in GraphPad Prism (v 10.2.0). For analysis of the anti-SGE IgG ELISA data (Supplementary Figure 2), the datasets for all time points were found to be lognormally distributed. Thus, we used log2-transformed data to analyze the IgG level comparing each time point to the pre-exposure level. Because some data points were missing due to study dropouts or a missed study visit, we used a mixed effects model with the Geisser-Greenhouse correction and Dunnett’s multiple comparisons test to perform the analysis.

For the IFN-γ ELISA (Figure 3), non-parametric statistical tests were used because the dataset failed both normality and lognormality tests. Friedman test with Dunn’s multiple comparisons test was used for pairwise comparisons of IFN-γ concentration between SGE (comparator) and each of the *Lu. longipalpis* recombinant salivary proteins. Mann-Whitney test was used to compare IFN-γ levels between *Lu. longipalpis*-exposed and unexposed participants.

For cytokine measurements by multiplex bead array (Figure 4 and Figure 5), the distributions of both raw and log2-transformed cytokine concentrations failed normality testing, so nonparametric tests were used for these analyses. For each cytokine, comparisons between the three treatment groups (SGE, LJM19, and LJL143) were performed with the Friedman test (due to having participant-matched samples) and Dunn’s test for multiple comparisons. To test for significant polarization towards T_H_1 or T_H_2, we calculated the log2 of the ratio of each T_H_1 cytokine (IFN-γ or IL-12) to each T_H_2 cytokine (IL-4, IL-5, IL-10, or IL-13), where a value of zero represents the null hypothesis, an equal balance of T_H_1 and T_H_2 cytokines. To each ratio, we then applied a Wilcoxon signed-rank test versus a value of zero to determine whether the cytokine ratio was significantly skewed towards T_H_1 or T_H_2.

For the *Leishmania* killing assay (Figure 6), within each of the two exposure groups (*Lu. longipalpis* Unexposed or Exposed) pairwise differences were analyzed between PBMCs stimulated with media alone versus each of the other treatment conditions. For the percentage of infected macrophages, the dataset passed all normality tests with participant-matched samples and three missing samples (*n* = 1 from Unexposed/media and *n* = 2 from *Lu. longipalpis* Exposed/media due to random failure of these macrophages to adhere and grow in culture), thus a mixed effects model was applied followed by Dunnett’s test for multiple comparisons. For the number of amastigotes per infected macrophage, the dataset failed normality testing, thus the non-parametric Kruskal-Wallis test was used for analysis. Because the log2 T_H_1/T_H_2 cytokine ratios are non-normally distributed, we applied Spearman’s test to determine the correlation between the log2 ratio of T_H_1/T_H_2 cytokines and the percentage of infected macrophages.

Differences were considered significant for *p* < 0.05 (*), however due to the exploratory nature of our study we have also indicated where *p* > 0.05 but < 0.1 (denoted by “#”) to identify potential trends that may be biologically relevant but did not achieve the significance threshold.

### Study Approval

The study was approved by the Institutional Review Boards of Walter Reed Army Medical Center, Walter Reed National Military Medical Center (protocol number WR355023), the National Institute of Allergy and Infectious Diseases (NIAID), and the Uniformed Services University of the Health Sciences (USUHS). All human subjects research was conducted according to the principles of the Declaration of Helsinki. All participants provided written informed consent prior to study participation. All participants provided written informed consent for use of their photographs; these informed consent records for photography have been retained by the investigators. The study was registered on www.clinicaltrials.gov as NCT01289977.

### Data Availability

All data values for all graphs are available on the accompanying Supporting Data Values file.

## RESULTS

### Characteristics of Study Participants

The demographics, allergy history, and baseline plasma IgE level of the study participants are presented in Table 1. The median age was 27 (range 22 to 46) years, and the majority were male (12 of 15, 80%). A schematic of the study design is presented in Figure 1. Participant #11 missed two sand fly exposures – one because of a medical hold due to delayed onset of significant localized pruritus and vesicles that lasted two days, and the other because of a scheduling conflict. There was significant loss to follow up starting at exposure #6 (Supplementary Figure 1). Most were due to active duty military participants being transferred out of the study area (n = 4) and one being discharged from the military (Participant #3). Three of 15 participants (20%) halted their participation due to study-related reactions to sand fly bites. Participants #6 and #7 both requested to withdraw from the study after exposure #6 due to developing urticaria, pruritus, a large area of erythema, delayed reactions at the bite site, and concern for potentially worse symptoms with future sand fly exposures. For Participant #9, sand fly exposures were halted by the investigator after exposure #5 due to the development of a large area of urticaria, pruritus, and erythema at the bite site, with delayed onset of pruritic papules. The number of fed sand flies was similar across all feedings, with an average of 8 to 9 fully fed and 0 to 1 partially fed sand flies out of 10 total per participant at each exposure.

### Progressive Exposures to *Lu. longipalpis* Alter the Bite Site Rash Phenotype

**Figure 2.**
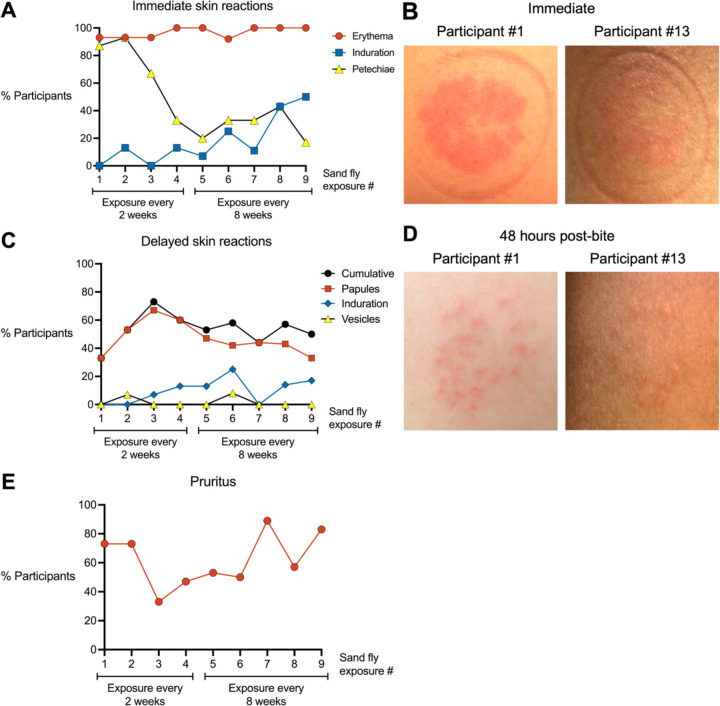
Characteristics of rashes at the sites of uninfected Lutzomyia longipalpis bites. (**A**) Skin reactions observed within the first 10 minutes after completion of each sand fly exposure. (**B**) Representative photographs from two participants immediately after Exposure #9. (**C**) Delayed skin reactions at the bite site that developed one or more days after completion of each sand fly exposure. (**D**) Representative photographs from two participants 48 hours after Exposure #9. (**E**) Pruritus at the bite site.

We qualitatively characterized the phenotype and symptoms of rashes induced by uninfected *Lu. longipalpis* bites and how these rash characteristics changed with successive sand fly exposures. For immediate bite site reactions, 85% to 100% of participants exhibited erythema across all exposures (Figure 2, A and B), consistent with the potent vasodilatory activity of the *Lu. longipalpis* salivary protein maxadilan (21). Petechiae showed a biphasic decrease with successive exposures, with only one of six participants exhibiting petechiae at the final exposure (#9). Induration was initially absent but overall showed an increasing trend to 50% of participants by the final exposure.

We next evaluated delayed bite site reactions that developed hours to one week after each sand fly exposure (Figure 2, C and D). Papules were the most frequent delayed rash phenotype (Figure 2C), increasing from 33% to 67% of participants over the first three exposures before declining to 33% by the final exposure. Induration and vesicles appeared inconsistently across exposures in a maximum of 23% and 8% of participants, respectively. Localized pruritus at the bite site (either delayed or immediate) was initially reported by 73% of participants and increased to 83% at the final exposure (Figure 2E). Across all exposures, the median pruritus duration was 2 days (range 30 min to 3 months).

Interestingly, for 8 of 15 participants we observed that sand fly exposure triggered the appearance of a rash at a previous bite site on the contralateral arm, which had been exposed weeks prior (median 6.5 weeks, range 2 to 11 weeks). This phenomenon, which we term distal bite site reactivation, occurred approximately one week after the most recent sand fly exposure after variable numbers of exposures (median 4.5, range 2 to 9).

### Development of Humoral Immunity to *Lu. longipalpis* Saliva

We next investigated how repeated sand fly exposures affect the development of antibodies directed against *Lu. longipalpis* salivary proteins. The concentration of plasma IgG that bound *Lu. longipalpis* salivary gland extract (SGE) was measured via ELISA (Supplemental Figure 2). The amount of anti-SGE IgG was significantly higher than pre-exposure levels starting in exposure #4 and was maintained in all subsequent exposures. These differences were largely driven by five individuals (#6, 11, 13, 14, and 15) whose anti-SGE IgG levels were notably higher than the other participants. These data demonstrate that progressive *Lu. longipalpis* exposure induces variable levels of IgG directed against sand fly saliva, with 10 out of 15 (66.7%) participants maintaining low anti-SGE IgG levels throughout the study period.

### *Lu. longipalpis* Bites Induce Cellular Interferon Gamma Responses to the Sand Fly Salivary Proteins LJL143 and LJM19

**Figure 3.**
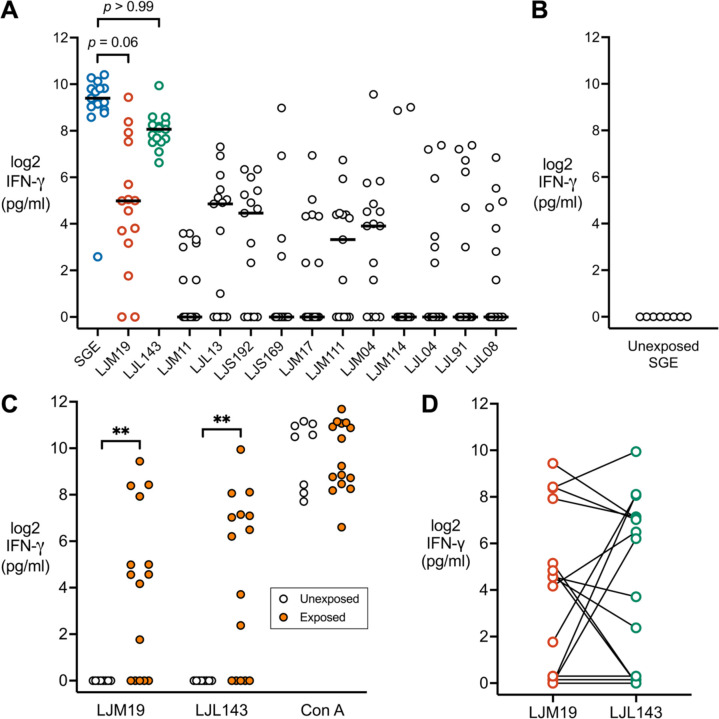
Recombinant salivary proteins stimulate IFN-γ production in PBMCs from Lu. longipalpis-exposed volunteers. (**A**) PBMCs were stimulated with salivary gland extract (SGE) or the indicated recombinant *Lu. longipalpis* salivary protein to measure IFN-γ levels by ELISA. PBMCs were from exposure #2 or #4 with one time point per volunteer (*n* = 15). *P*-values are shown only for comparisons with *p* > 0.05 that showed no significant difference from SGE. (**B**) As a negative control, IFN-γ was measured in SGE-stimulated PBMCs from healthy volunteers not exposed to *Lu. longipalpis* (*n* = 8). (**C**) IFN-γ secretion in PBMCs from individuals exposed (*n* = 15) or unexposed (*n* = 8) to *Lu. longipalpis* after stimulation with LJL143 or LJM19. Concavalin A (2.5 μg/ml) was used as a positive control. PBMCs were from exposures #4 to #9 with one time point per individual. (**D**) Re-plot of the same data from exposed individuals in **C** to show the response profile to LJL143 and LJM19 for each participant (line and its connecting points). *P*-values were calculated by Friedman test with Dunn’s multiple comparisons test in **A** or by Mann-Whitney test in **C**, ** *p* < 0.01.

Prior studies in mouse and non-human primate models have demonstrated that repeated exposure to sand fly bites leads to the development of a strong IFN-γ response to subsequent sand fly challenge and that IFN-γ is a correlate of protection against leishmaniasis (9, 14). To assess whether a similar cellular IFN-γ response develops in humans, PBMCs from sand fly-bitten volunteers were collected after their second or fourth sand fly exposure and stimulated with SGE. PBMCs from all participants produced IFN-γ after SGE stimulation (Figure 3A), whereas no IFN-γ production was elicited by SGE treatment of PBMCs from *Lu. longipalpis*-unexposed individuals obtained from the NIH Blood Bank (Figure 3B). These results suggest that repeated exposure of humans to *Lu. longipalpis* bites leads to the development of a robust cellular IFN-γ response to sand fly saliva.

To identify specific *Lu. longipalpis* salivary proteins that induce a strong cellular IFN-γ response, we screened the thirteen most abundant proteins in *Lu. longipalpis* saliva for their ability to stimulate IFN-γ production from PBMCs collected after the second or fourth sand fly exposure. The proteins screened include homologs from the anti-complement family (LJM19, LJS192, and LJS169), the yellow-related protein family (LJM17, LJM11, and LJM111), the small odorant protein family (LJM04 and LJM114) and single proteins unrelated to the other salivary protein families (LJL143, LJL13, LJL04, LJL91, and LJL08) (Figure 3A). LJL143 and LJM19 were the only salivary proteins that stimulated IFN-γ production at a level comparable to SGE; stimulation with any of the other eleven *Lu. longipalpis* salivary proteins resulted in significantly less IFN-γ production (all *p* < 0.01). In this initial screening experiment, PBMCs from all individuals responded to LJL143, while two individuals did not respond to LJM19.

We then sought to assess the robustness of LJL143 and LJM19 as IFN-γ-inducing salivary proteins by stimulating a different batch of PBMCs collected at later sand fly exposures, ranging from exposure numbers four through nine (Figure 3C). In this follow-up experiment, we observed the maintenance of strong IFN-γ responses to both LJL143 and LJM19 among responders. However, there were 5 non-responders (33%) to each of these two salivary proteins. Further analysis of individual participant IFN-γ response profiles (Figure 3D) showed only 3 non-responders to either protein, with 2 only responding to LJL143, and another 2 to LJM19. These stimulation assays demonstrate that cellular IFN-γ responses to LJL143 or LJM19 developed in the majority of volunteers exposed to multiple *Lu. longipalpis* bites.

### LJM19 and LJL143 Induce a T_H_1-Polarized Cytokine Response

**Figure 4.**
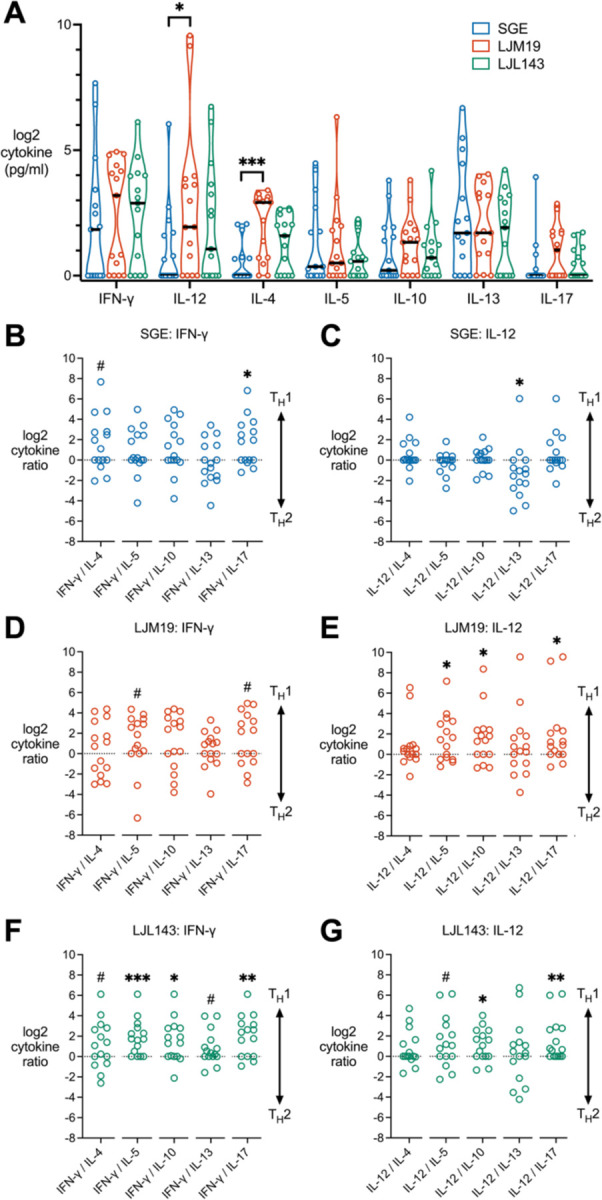
LJM19 and LJL143 induce TH1-polarized cytokine responses in PBMCs from Lu. longipalpis-exposed volunteers. (**A**) PBMCs obtained from sand fly-exposed study participants (*n* = 15) were stimulated with SGE, LJM19, or LJL143. Cell supernatants were collected at 96 hours and cytokine concentrations were measured by multiplex bead array. Black bar indicates the median. For each cytokine, differences between treatment groups were analyzed by Friedman test with Dunn’s test for multiple comparisons. PBMCs were from exposure #4 to #9 with one time point per participant). Ratios of TH1 to TH2/TH17 cytokines were calculated for PBMCs treated with SGE (**B** and **C**), LJM19 (**D** and **E**), or LJL143 (**F** and **G**). Ratios above 0 indicate a TH1-polarized response while ratios below 0 indicate a TH2 or TH17-polarized response, as analyzed by Wilcoxon signed-rank test using a value of zero as the null hypothesis (equal balance of TH1 and TH2/TH17 cytokines), # *p* < 0.1, * *p* < 0.05, ** *p* < 0.01, *** *p* < 0.001.

We next sought to determine how LJM19 and LJL143 stimulation affects the overall T_H_ polarization profile. PBMCs collected after exposure numbers four through nine were stimulated with SGE, LJM19, or LJL143, and cytokines were measured in the supernatants by multiplex bead array. In comparing the three stimulation conditions for each T_H_ cytokine, we found that PBMCs produced a mixed cytokine response, with LJM19 inducing significantly higher levels of IL-12 and IL-4 compared to SGE (Figure 4A). Notably, of the T_H_2 cytokines IL-13 was consistently the highest expressed, at levels comparable to IFN-γ and IL-12 across all three treatments.

Because T_H_ polarization depends on the relative concentration of cytokines from different functional classes, we calculated the ratios of the T_H_1 cytokines (IFN-γ and IL-12) against each of the T_H_2 cytokines (IL-4, IL-5, IL-10, and IL-13), or to the T_H_17 cytokine, IL-17. SGE induced a largely mixed T_H_1/T_H_2 response with only a trend towards T_H_1 seen for the IFN-γ/IL-4 ratio (Figure 4B) and significant T_H_2 polarization for the IL-12/IL-13 ratio (Figure 4C). Treatment with LJM19 induced a comparatively weak IFN-γ response, with a trend towards T_H_1 seen only for IFN-γ/IL-5 (Figure 4D). In contrast, significant T_H_1 polarization was observed for IL-12 relative to the T_H_2 cytokines IL-5 and IL-10 (Figure 4E). LJL143 induced a strong IFN-γ response with significant T_H_1 polarization seen relative to IL-5 and IL-10 and a trend for IL-4 and IL-13 (Figure 4F). IL-12 also showed significant T_H_1 polarization against IL-10 and a trend against IL-5 (Figure 4G). None of the treatments induced a T_H_17-polarized response.

Examining the T_H_ cytokine profile of individual participants, we found the T_H_ cytokine ratios across either IFN-γ or IL-12 were largely consistent for each individual (Supplemental Figure 3). The exception to this was IL-13, which was consistently expressed at a higher level than the other T_H_2 cytokines. Following LJM19 stimulation, the participant-specific IFN-γ/T_H_2 ratio profiles revealed a notable segregation between T_H_1-polarized versus mixed or T_H_2-polarized individuals (Supplemental Figure 3C), producing a more variable response compared to LJL143 (Supplemental Figure 3E). In summary, LJL143, and to a lesser extent LJM19, induce a T_H_1-polarized response in PBMCs from individuals with multiple *Lu. longipalpis* exposures. Of note, this T_H_1 response is observed though the induction of both IFN-γ and IL-12 by LJL143, and primarily by IL-12 for LJM19.

### LJM19 and LJL143 Induce Inflammatory and Antiviral Cytokines

**Figure 5.**
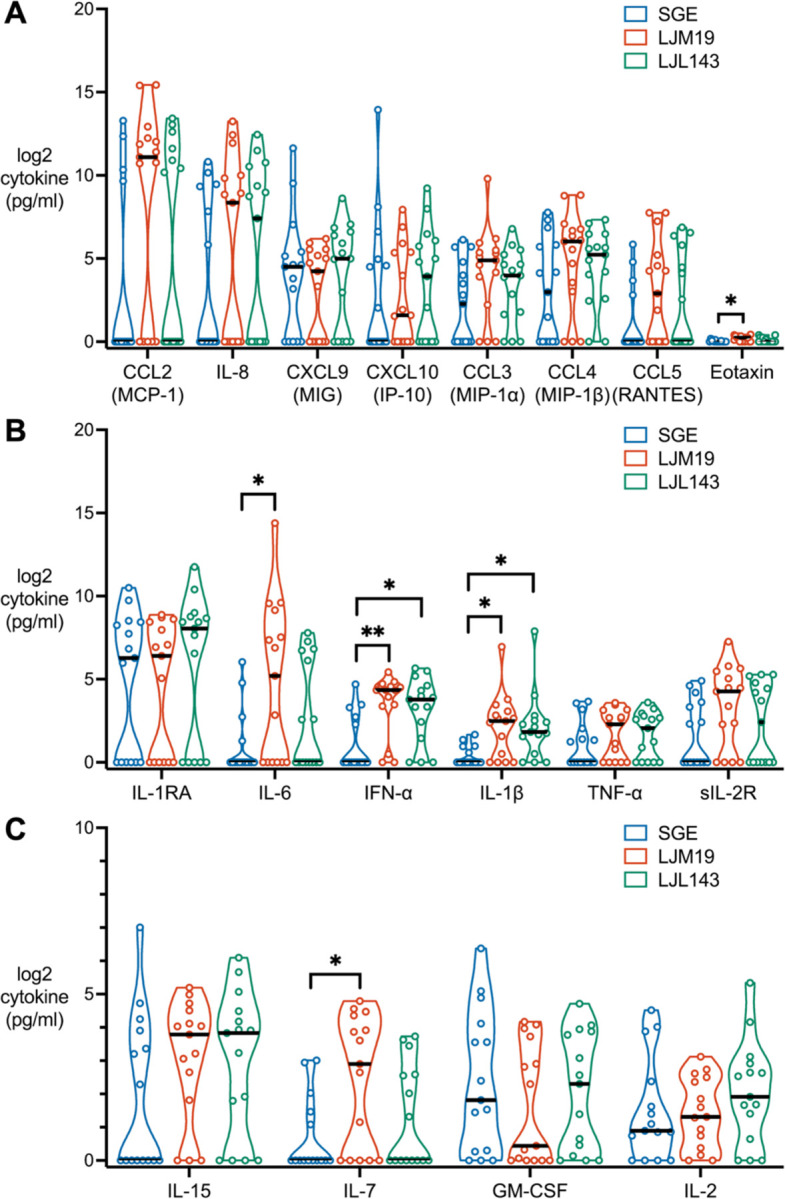
Differences in PBMC cytokine profiles induced by LJM19 or LJL143 compared to SGE. PBMCs obtained from *Lu. longipalpis*-exposed study participants (*n* = 15) were stimulated with SGE, LJM19, or LJL143. Cell supernatants were collected at 96 hours and cytokine concentrations were measured by multiplex bead array for chemokines (**A**), inflammatory cytokines (**B**), and cytokines promoting cell survival, activation, and proliferation (**C**). PBMCs used were from exposure #4 to #9 with one time point per participant). Black bar indicates the median. For each cytokine, differences between treatment groups were analyzed by Friedman test with Dunn’s test for multiple comparisons, * *p* < 0.05, ** *p* < 0.01.

We next investigated the effects of SGE, LJM19, and LJL143 on other functional classes of cytokines. Generally, CCL2, IL-8, and IL-1RA were strongly induced across all treatments (Figure 5, A and B). Among chemokines, LJM19 induced a significantly higher level of eotaxin than SGE, however the concentration produced by all treatment groups was negligible relative to the levels of other chemokines (Figure 5A). Compared to SGE, both LJM19 and LJL143 induced significantly higher levels of interferon alpha (IFN-α), an antiviral and immunomodulatory cytokine, and IL-1β, an inflammatory cytokine (Figure 5B). Additionally, LJM19 induced higher levels of IL-6 (Figure 5B), an inflammatory cytokine, and IL-7 (Figure 5C), which promotes lymphocyte proliferation and maintenance in peripheral tissues (22).

### LJL143 Enhances the Ability of PBMCs to Stimulate Macrophage Killing of Intracellular *Leishmania* Parasites

**Figure 6.**
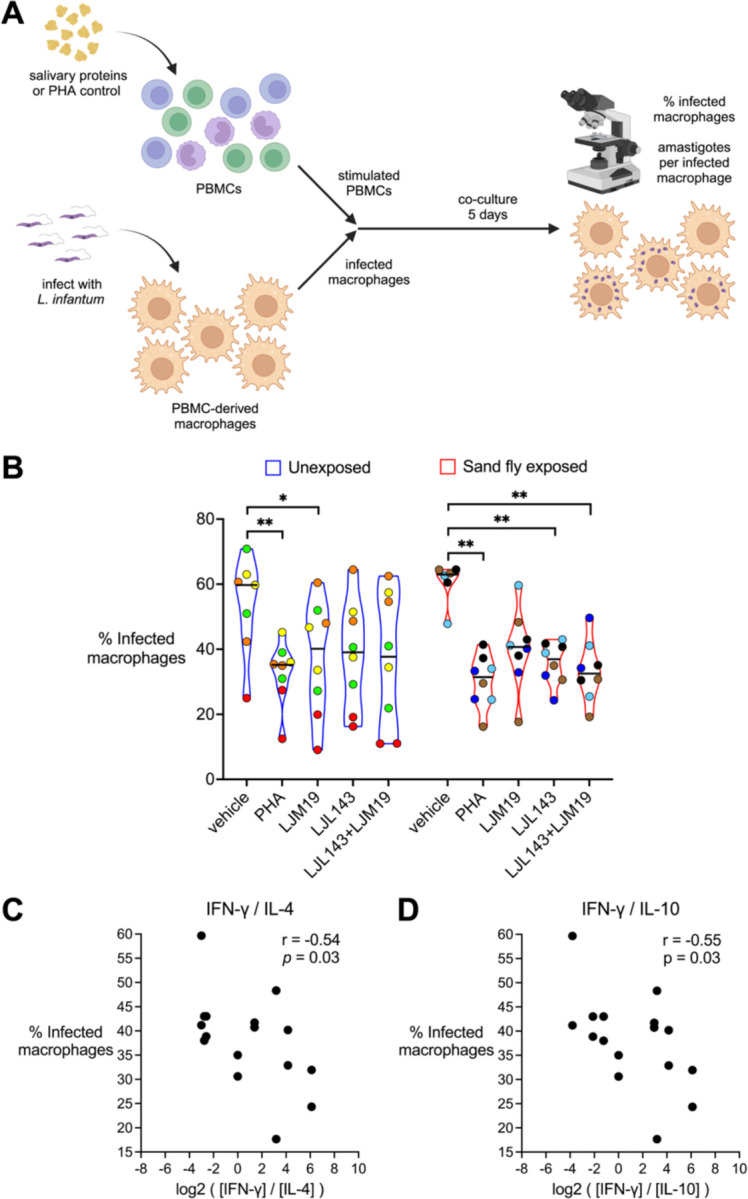
Stimulation of PBMCs with LJL143 enhances killing of Leishmania by macrophages. PBMC-derived macrophages from participants unexposed (*n* = 4, blue) or exposed (*n* = 4, red) to *Lu. longipalpis* were infected with *Leishmania infantum*, then co-cultured in the presence or absence of autologous PBMCs that had been stimulated according to the different conditions shown. After 5 days of co-culture, the percentage of infected macrophages was quantified by manual counting of Giemsa-stained cells by light microscopy. PBMCs were from exposure #7 to #9 with one time point per participant. (**A**) Experimental scheme. (**B**) Percentage of infected macrophages. Each volunteer’s batch of PBMCs was divided and run in two technical replicates; identical colors denote replicates from the same individual. Bar indicates the median. Differences between treatment groups were analyzed via a mixed effects model with Dunnett’s test for multiple comparisons, # *p* < 0.1, * *p* < 0.05, and ** *p* < 0.01. (**C** and **D**) Spearman correlation between the IFN-γ/IL-4 ratio (**C**) or IFN-γ/IL-10 ratio (**D**) as calculated in Figure 4 and the percentage of infected macrophages for LJM19- and LJL143-treated samples. Schematic in **A** created in BioRender.com.

To test the hypothesis that treatment with LJM19 or LJL143 enhances the ability of PBMCs to stimulate the killing of intracellular parasites by *Leishmania*-infected macrophages, we compared PBMCs from *Lu. longipalpis*-naïve individuals from the NIH Blood Bank (Unexposed) to PBMCs from sand fly exposed volunteers. For the latter, we used PBMCs collected after exposures #7 through #9 from a subset of four participants who completed all nine scheduled sand fly exposures. Monocyte-derived macrophages were infected with *L. infantum*, then co-cultured with autologous PBMCs stimulated with the sand fly salivary proteins LJM19 or LJL143. Phytohemagglutinin (PHA), a T cell mitogen, served as a positive control (Figure 6A).

For *Lu. longipalpis*-exposed participants, PBMC stimulation with either LJL143 or a combination of LJL143 and LJM19 led to a significant reduction in the percentage of infected macrophages comparable to that observed with PHA, while a trend towards a decrease was seen with LJM19 alone (Figure 6B). Interestingly, in PBMCs from *Lu. longipalpis* unexposed individuals, stimulation with LJM19 alone induced a significant decrease in the percentage of infected macrophages, while stimulation with LJL143 showed a trend towards a decrease, potentially caused by their innate immune properties. There were no significant differences between media alone and any of the treatment groups in the number of amastigotes per infected macrophage (Supplemental Figure 4).

Cross-referencing these macrophage infection data with our earlier T_H_ cytokine ratio results revealed a significant negative correlation between the percentage of infected macrophages and the ratios IFN-γ/IL-4 and IFN-γ/IL-10 (Figure 6C), while no significant correlation was seen with IFN-γ alone, IFN-γ/IL-5, or IFN-γ/IL-13 (Supplemental Figure 5).

### *Lu. longipalpis* Bites Induce a Delayed Type Hypersensitivity Skin Response After Multiple Sand Fly Exposures

**Figure 7.**
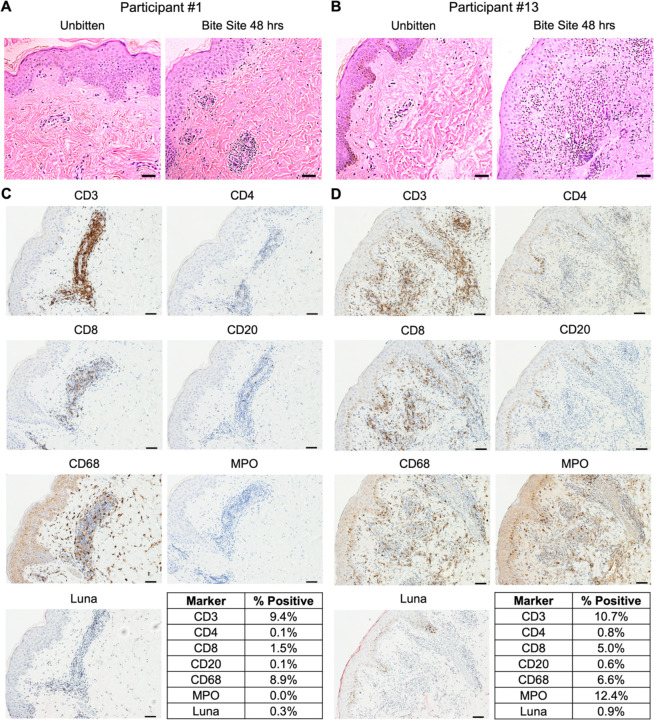
Repeated exposures to uninfected Lu. longipalpis bites induce a delayed-type hypersensitivity response at the bite site. Skin punch biopsies were collected from bite site skin and from the contralateral arm (negative control) from two participants 48 hours after exposure #9. (**A** and **B**) Hematoxylin and eosin stains. (**C** and **D**) Immunohistochemistry (IHC) of the indicated markers at the bite site. Tables show the percentage of cells that stained positive for each marker based on analysis with ImageJ. Scale bar is 50 μm for all images.

Forty-eight hours after the final sand fly exposure, two participants (#1 and #13) who completed all nine exposures volunteered for collection of skin biopsies from a bite site and from the contralateral arm as a negative control. Both participants exhibited multiple erythematous papules at their exposure site, characteristic of a DTH response (Figure 2D). Histology from Participant #1 showed spongiosis and a perivascular mononuclear (lymphocytic and histiocytic) infiltrate with occasional eosinophils (Figure 7A). Participant #13 exhibited a more diffuse, mixed inflammatory infiltrate with both neutrophils and mononuclear cells present in the upper dermis and distributed throughout the perivascular, interstitial, and perieccrine areas. (Figure 7B). Immunohistochemistry (IHC) reveals the infiltrate in both participants is largely composed of T cells (CD3) and macrophages (CD68), while B cells (CD20) and eosinophils (Luna) were rare or absent, respectively (Figure 7, C and D). Interestingly, CD4 signal is minimal (Participant #13) to absent (Participant #1), while the CD8 signal overlaps with a minority of the CD3 staining pattern. Taken together, the CD4 and CD8 signals fail to fully account for the strong CD3 signal observed in both individuals. While Participant #1 had no detectable neutrophils by myeloperoxidase (MPO) stain (Figure 7C), Participant #13 showed MPO staining throughout the dermis (Figure 7D). These data highlight macrophages and T cells as the predominant immune cells driving the DTH response to *Lu. longipalpis* bites in these two individuals. The IHC data also raise the possibility that CD4^–^CD8^–^ T cells (also termed double negative or DN T cells) play a role in the DTH response to sand fly bites.

**Figure 8.**
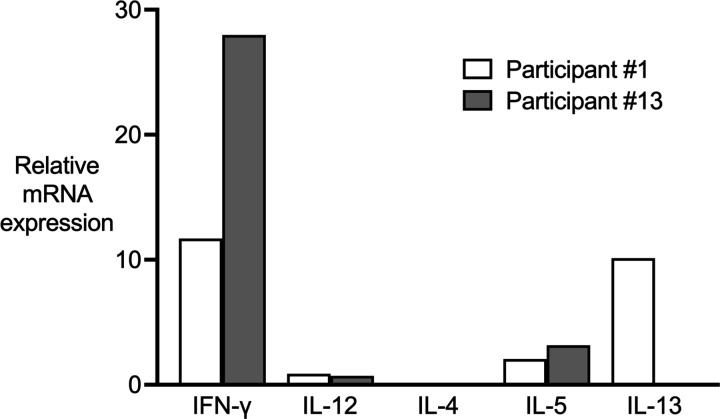
Skin cytokine profiles of the delayed type hypersensitivity response to Lu. longipalpis bites. Skin punch biopsies were collected as described in Figure 7 for measurement of cytokine mRNA expression by quantitative RT-PCR. For each participant, gene expression at the bite site was normalized to expression in normal appearing skin from the contralateral arm.

Quantitative RT-PCR of the skin biopsies elucidated the cutaneous T_H_ cytokine profile at the bite site (Figure 8). Participant #1 exhibited a mixed T_H_1/T_H_2 response with similar levels of IFN-γ and IL-13 and only minor contributions from IL-12 and IL-5. In contrast, Participant #13 showed a dominant T_H_1 response driven by high expression of IFN-γ, no detectable IL-13, and comparatively low expression of IL-12 and IL-5.

## DISCUSSION

This study, together with a related human sand fly challenge study with the Eastern hemisphere sand fly *Phlebotomus duboscqi* (19), provides the most comprehensive characterization to date of how human skin responses to sand fly saliva evolve with cumulative bite exposure over nearly a year. While acknowledging the small size of the cohort (*n* = 15) and the ensuing study dropout rate, we observed the development of stable DTH reactions to *Lu. longipalpis* bites that were maintained throughout the study, similar to the durable DTH responses seen in a cohort in Mali following decades of exposure to *Phlebotomus duboscqi* (6). A saliva-specific T_H_1-polarized DTH response has been closely correlated to immune protection against *Leishmania* (6, 9, 12, 14).

In over half of participants, *Lu. longipalpis* bites triggered the reappearance of a rash on the contralateral arm at a site that had been bitten several weeks prior. This phenomenon of distal bite site reactivation was similarly reported following uninfected *Phlebotomus duboscqi* bites (19) and during the early characterization of human skin reactions to the bites of *Phlebotomus papatasi* (23). We speculate that this phenomenon reflects the activation of salivary protein-specific, skin resident memory T cells that have seeded a prior bite site and respond to circulating salivary antigens derived from the new sand fly bite (24, 25). The ability of vector bites to impact systemic immunity was demonstrated in humanized mice exposed to uninfected *Aedes aegypti* bites (26). Bitten mice exhibited significant changes in blood cytokine levels and the composition of immune cells in both the blood and skin up to 7 days after mosquito exposure. An intriguing possibility is that the local cutaneous response to a vector bite triggers systemic signals that orchestrate a pathogen-resistant state throughout the skin compartment, similar to the organism-wide coordination of antiviral immunity (27, 28).

Our screen identified LJM19 and LJL143 as the two *Lu. longipalpis* salivary proteins that stimulate the highest IFN-γ production in PBMCs from sand fly exposed participants, confirming their immunogenicity in humans. LJM19 was previously identified as a salivary protein vaccine candidate that protected against both cutaneous and visceral leishmaniasis in preclinical models (15, 16, 29, 30). LJM19 (also named SALO) is an 11 kDa protein that inhibits the classical complement pathway and has no structural similarity to any human proteins (31, 32). LJL143 (also named Lufaxin) is an inhibitor of coagulation factor Xa and the alternative pathway of complement (33, 34), and was identified in a salivary protein screen in dogs as eliciting strong DTH and IFN-γ responses in the skin and blood following uninfected sand fly challenge (13). Adjuvant-like activity of LJL143 was demonstrated in a mouse model testing the immunogenicity of a virus-like particle (VLP) vaccine composed of LJL143 and two *Leishmania* antigens (15). Priming with unadjuvanted LJL143 before boosting with the VLP vaccine induced higher CD4+ T cell proliferative responses to *Leishmania* antigens. Despite its immunogenicity, LJL143 has shown only partial or no protection in mice challenged with *L. major* co-inoculated with saliva (35) or infected sand fly bite (36). In contrast, our data from human PBMCs shows that LJL143 induces strong T_H_1 polarization and promotes killing of *L. infantum* by infected macrophages *ex vivo*. This contrast highlights the importance of assessing sand fly salivary protein vaccine candidates in a human immune context, where the immune profile and protection elicited by these proteins may differ from what is observed in preclinical models. Of note, LJL143 and its homologs show strong sequence conservation between Eastern and Western hemisphere sand flies, highlighting LJL143 as a candidate component of a pan-*Leishmania* vaccine (36).

In PBMCs from participants exposed to *Lu. longipalpis*, SGE induced a mixed T_H_1/T_H_2 response, while LJM19 and LJL143 both induced T_H_1 responses; this was characterized by high expression of IL-12 in LJM19, compared to high expression of both IFN-γ and IL-12 in LJL143. Classically, IL-12 production by antigen presenting cells leads to IFN-γ production by T cells (37), however as seen for LJM19, strong induction of IL-4 can suppress IFN-γ despite high levels of IL-12 (38). High co-expression of IL-12 and IL-4 mirrors the cytokine profile seen in the draining lymph nodes and spleen of LJM19-immunized hamsters protected against *L. donovani* challenge (16), reflecting a potentially conserved cytokine response to LJM19.

Of the T_H_2 cytokines, IL-13 was often expressed at levels equal to or higher than IFN-γ. In both mice and humans, IL-13 enhances *Leishmania* infection and exacerbates immunopathology (39–41), in part by inhibiting the expression of IL12Rβ2, which transduces critical signals for T_H_1 differentiation (42, 43). In support of this, polymorphisms at the *IL13* locus in humans are associated with resistance or susceptibility to CL caused by *L. guyanensis* (44). Of note, in a naturally exposed population in Mali, IL-13 was expressed upon stimulation of PBMCs with saliva of *P. duboscqi* but not at the bite site after challenge with uninfected bites (6). Additional studies are needed to determine whether the high IL-13 expression induced by LJM19 and LJL143 reflects an intrinsic property of these proteins or is a counterregulatory response to T_H_1 polarization.

One enduring question has been the mechanism by which sand fly salivary proteins polarize towards a T_H_1 or T_H_2 response in the absence of adjuvant (45). One hypothesis is that the T_H_-polarizing capacity of these proteins is determined by the properties of the MHC class II-binding peptides derived from them (46, 47). Conformational stability has also been linked to the ability of proteins to polarize T_H_ effector functions (48). Distinguishing between these mechanisms experimentally will help optimize the antigenic features of LJM19 and LJL143 for incorporation into vaccines against leishmaniasis. Furthermore, sand fly salivary proteins can be used as a model system to dissect the mechanisms by which natural adjuvants drive T_H_ polarization.

We discovered that LJM19 induces IL-6 and IL-7, while both LJM19 and LJL143 induce IL-1β and the antiviral cytokine IFN-α, a type I interferon. Induction of these innate immune mediators contrasts with the reported downregulation of IL-6 and IL-1β expression in immune cells by PpSP32, another sand fly salivary protein (49). Additionally, to our knowledge the current study is the first to report altered expression of IL-7 and IFN-α in response to specific sand fly salivary proteins. IL-6 exerts pleiotropic effects by promoting the acute phase response, granulopoiesis, B cell proliferation, and CD8+ T effector cell development (50). In mice, IL-6 has been shown to facilitate resistance to *L. major* (51) and *L. donovani* (52), in the latter case by inhibiting the proliferation of IL-10-expressing CD4+ T cells. IL-7 promotes the survival, proliferation, and maintenance of T cells in peripheral tissues (22), including skin resident memory T cells (53) which are critical for sustained protection against *Leishmania* (54), and is thus an attractive feature of a vaccine candidate. While IL-1β has been reported to protect against *L. amazonensis* (55), the majority of studies implicate IL-1β in exacerbating parasite dissemination and immunopathology (56–60). Similarly, IFN-α likely benefits the parasite by acting on dendritic cells to antagonize IFN-γ responses and suppress the development of *Leishmania*-specific T cells (61, 62), though the timing of IFN-α signaling relative to *Leishmania* infection can shift the balance between protection and susceptibility (63).

The discovery that stimulation of human PBMCs with LJM19 or LJL143 decreased the percentage of infected macrophages in co-culture, even in cells from unexposed individuals who tested negative for anti-saliva IgG, is consistent with the hypothesis that LJM19 and LJL143 have intrinsic adjuvant properties in addition to their induction of robust adaptive immune responses. Whether these salivary proteins bind yet-unidentified innate immune receptors and the identity of the cell subsets which mediate their *Leishmania*-protective effects remain to be explored. Interestingly, the number of amastigotes per infected macrophage were similar when comparing samples treated with media versus those treated with LJM19 or LJL143. This suggests that these salivary proteins act at an early step of macrophage infection, either blocking parasite invasion or promoting killing of *Leishmania* shortly after invasion, before the parasite establishes in the phagolysosome.

Skin biopsies of sand fly bite sites from two participants who completed all nine sand fly exposures exhibited a DTH response composed of T cells, macrophages, and in one participant, neutrophils. Strikingly, both sites were dominated by CD3+ CD4– CD8– T cells, also called “double negative” (DN) T cells. A similar IHC pattern suggestive of DN T cells was seen by histology in DTH reactions to *P. duboscqi* bites in humans from a CL-endemic region of Mali (6), but not in humans from a non-endemic area (19). While the role of DN T cells in response to sand fly bites remains unclear, expanded DN T cell populations have been demonstrated in human CL lesions due to *L. braziliensis* and exhibit a highly activated phenotype (64, 65). In these lesions, 75% of DN T cells express an αβ T cell receptor (TCR) and show high expression of IFN-γ and CD69, a tissue residence marker (64). DN T cells also compose the largest proportion of cytotoxic CD107a+ cells in human *L. braziliensis* skin lesions (65). DN T cells are necessary for primary and secondary immunity to *L. major* in mice, where these cells predominantly express TCR αβ and the central memory markers CD62L and CD44, are MHC class II-restricted, and produce IFN-γ (66). Further studies are needed to investigate whether DN T cells are a consistent and prominent feature of the DTH response to sand fly bites and how sand fly saliva-primed DN T cells influence immunity to *Leishmania* parasites.

Our study has several limitations. The study cohort is small and relatively homogenous demographically, thereby limiting our ability to generalize these results to CL endemic populations. Due to the limited recovery of PBMCs from some blood collections, each assay used PBMCs from a range of different time points, though within each assay, only one time point was used per volunteer. This heterogeneity prevented us from identifying temporal trends. Additionally, our macrophage parasite killing assay could only be performed on a minority of the cohort (4 of 15 total, and 4 of 6 who completed all nine sand fly exposures). While we cannot exclude the possibility that participants were previously exposed to local *Lutzomyia* species at low prevalence (67), all participants and blood bank samples were screened and found to be negative for IgG against *Lu. longipalpis* SGE. We had significant study attrition with 9 of 15 participants failing to complete all scheduled sand fly exposures. However, only 3 of the dropouts were study related, and all participants completed at least 5 exposures over 14 weeks, which should be sufficient to develop adaptive immunity to sand fly salivary antigens. Finally, most of our findings are based on PBMC responses, and need to be reinforced by investigating the responses of immune cells in the skin.

In summary, we report that humans exposed to *Lu. longipalpis* generate robust innate and adaptive cellular immune responses to the sand fly salivary proteins LJM19 and LJL143. These proteins exhibit adjuvant-like activity, induce T_H_1 polarization, and enhance the protection of macrophages against *Leishmania* infection, highlighting the potential of co-opting anti-saliva immunity to protect humans against vector borne pathogens. This work illustrates the power of using vector human challenge studies to elucidate the effects of chronic exposure to vector salivary antigens on human immunity, a daily occurrence in regions where these vectors are prevalent.

## Supplementary Material

Supplement 1

## Figures and Tables

**Table 1.** Study participant characteristics.

Participant ID	Completed Sand Fly Exposure #	Age	Sex	Race	Allergy History	Baseline Plasma IgE (kU/L)	Reason for Early Withdrawal or Skipped Feedings
1	9	26	M	White	None	17.9	-
2	5	26	M	White	None	9.9	Military transfer orders
3	6	26	M	Asian	Seasonal allergies Asthma	114.0	Discharged from military
4	8	40	M	White	Asthma	6.7	Military transfer orders
5	7	29	M	White	None	3.3	Military transfer orders
6	6	34	M	White	None	19.9	Participant request, adverse event
7	6	45	M	Black	Seasonal allergies Allergic to shellfish Eczema Asthma	20.7	Participant request, adverse event
8	9	22	M	White	None	96.6	-
9	5	26	M	Asian	Seasonal allergies	6.4	Medically advised to stop due to adverse event
10	5	24	M	White	Anaphylaxis to raisins	15.3	Military transfer orders
11	7	46	M	White	Seasonal allergies	<2.0	Two feedings skipped (medical hold, schedule conflict)
12	9	27	F	White	Urticaria to acetaminophen-hydrocodone Bee allergy Asthma	26.0	-
13	9	26	F	Multiracial	Seasonal allergies Angioedema to shellfish	61.1	-
14	9	41	M	White	Seasonal allergies Asthma	77.2	-
15	9	28	F	White	Urticaria to amoxicillin-clavulanic acid	6.7	-

## References

[1] WHO. Leishmaniasis: WHO Fact Sheet. https://www.who.int/news-room/fact-sheets/detail/leishmaniasis. Accessed 11 Dec, 2024.

[2] Serafim TD, Coutinho-Abreu IV, Dey R, Kissinger R, Valenzuela JG, Oliveira F, and Kamhawi S. Leishmaniasis: the act of transmission. Trends Parasitol. 2021;37(11):976–87. 34389215 10.1016/j.pt.2021.07.003

[3] Lakhal-Naouar I, Mukbel R, DeFraites RF, Mody RM, Massoud LN, Shaw D, The human immune response to saliva of Phlebotomus alexandri, the vector of visceral leishmaniasis in Iraq, and its relationship to sand fly exposure and infection. PLoS Negl Trop Dis. 2021;15(6):e0009378. 34081700 10.1371/journal.pntd.0009378 PMC8174707

[4] Vinhas V, Andrade BB, Paes F, Bomura A, Clarencio J, Miranda JC, Human anti-saliva immune response following experimental exposure to the visceral leishmaniasis vector, Lutzomyia longipalpis. Eur J Immunol. 2007;37(11):3111–21. 17935072 10.1002/eji.200737431

[5] Kammoun-Rebai W, Bahi-Jaber N, Naouar I, Toumi A, Ben Salah A, Louzir H, and Meddeb-Garnaoui A. Human cellular and humoral immune responses to Phlebotomus papatasi salivary gland antigens in endemic areas differing in prevalence of Leishmania major infection. PLoS Negl Trop Dis. 2017;11(10):e0005905. 29023574 10.1371/journal.pntd.0005905 PMC5638224

[6] Oliveira F, Traore B, Gomes R, Faye O, Gilmore DC, Keita S, Delayed-type hypersensitivity to sand fly saliva in humans from a leishmaniasis-endemic area of Mali is Th1-mediated and persists to midlife. J Invest Dermatol. 2013;133(2):452–9. 22992802 10.1038/jid.2012.315 PMC3529997

[7] Carvalho AM, Viana SM, Andrade BB, Oliveira F, Valenzuela JG, Carvalho EM, and de Oliveira CI. Immune Response to LinB13, a Lutzomyia Intermedia Salivary Protein Correlates With Disease Severity in Tegumentary Leishmaniasis. Clin Infect Dis. 2022;75(10):1754–62. 35385578 10.1093/cid/ciac258 PMC9662176

[8] Mondragon-Shem K, Al-Salem WS, Kelly-Hope L, Abdeladhim M, Al-Zahrani MH, Valenzuela JG, and Acosta-Serrano A. Severity of old world cutaneous leishmaniasis is influenced by previous exposure to sandfly bites in Saudi Arabia. PLoS Negl Trop Dis. 2015;9(2):e0003449. 25646796 10.1371/journal.pntd.0003449 PMC4315490

[9] Kamhawi S, Belkaid Y, Modi G, Rowton E, and Sacks D. Protection against cutaneous leishmaniasis resulting from bites of uninfected sand flies. Science. 2000;290(5495):1351–4. 11082061 10.1126/science.290.5495.1351

[10] Kima PE, and Soong L. Interferon gamma in leishmaniasis. Front Immunol. 2013;4:156. 23801993 10.3389/fimmu.2013.00156 PMC3685816

[11] Aoki V, Abdeladhim M, Li N, Cecilio P, Prisayanh P, Diaz LA, and Valenzuela JG. Some Good and Some Bad: Sand Fly Salivary Proteins in the Control of Leishmaniasis and in Autoimmunity. Front Cell Infect Microbiol. 2022;12:839932. 35281450 10.3389/fcimb.2022.839932 PMC8913536

[12] Fayaz S, Bahrami F, Parvizi P, Fard-Esfahani P, and Ajdary S. An overview of the sand fly salivary proteins in vaccine development against leishmaniases. Iran J Microbiol. 2022;14(6):792–801. 36721440 10.18502/ijm.v14i6.11253 PMC9867623

[13] Collin N, Gomes R, Teixeira C, Cheng L, Laughinghouse A, Ward JM, Sand fly salivary proteins induce strong cellular immunity in a natural reservoir of visceral leishmaniasis with adverse consequences for Leishmania. PLoS Pathog. 2009;5(5):e1000441. 19461875 10.1371/journal.ppat.1000441 PMC2677456

[14] Oliveira F, Rowton E, Aslan H, Gomes R, Castrovinci PA, Alvarenga PH, A sand fly salivary protein vaccine shows efficacy against vector-transmitted cutaneous leishmaniasis in nonhuman primates. Sci Transl Med. 2015;7(290):290ra90. 10.1126/scitranslmed.aaa3043 26041707

[15] Cecilio P, Perez-Cabezas B, Fernandez L, Moreno J, Carrillo E, Requena JM, Pre-clinical antigenicity studies of an innovative multivalent vaccine for human visceral leishmaniasis. PLoS Negl Trop Dis. 2017;11(11):e0005951. 29176865 10.1371/journal.pntd.0005951 PMC5720812

[16] Fiuza JA, Dey R, Davenport D, Abdeladhim M, Meneses C, Oliveira F, Intradermal Immunization of Leishmania donovani Centrin Knock-Out Parasites in Combination with Salivary Protein LJM19 from Sand Fly Vector Induces a Durable Protective Immune Response in Hamsters. PLoS Negl Trop Dis. 2016;10(1):e0004322. 26752686 10.1371/journal.pntd.0004322 PMC4708988

[17] Diaz-Dinamarca DA, Salazar ML, Castillo BN, Manubens A, Vasquez AE, Salazar F, and Becker MI. Protein-Based Adjuvants for Vaccines as Immunomodulators of the Innate and Adaptive Immune Response: Current Knowledge, Challenges, and Future Opportunities. Pharmaceutics. 2022;14(8). 10.3390/pharmaceutics14081671 PMC9414397 36015297

[18] Sangare M, Coulibaly YI, Huda N, Vidal S, Tariq S, Coulibaly ME, Individuals co-exposed to sand fly saliva and filarial parasites exhibit altered monocyte function. PLoS Negl Trop Dis. 2021;15(6):e0009448. 34106920 10.1371/journal.pntd.0009448 PMC8189443

[19] de Araujo FF, Abdeladhim M, Teixeira C, Hummer K, Wilkerson MD, Ressner R, Immune response profiles from humans experimentally exposed to Phlebotomus duboscqi bites. Front Immunol. 2024;15:1335307. 38633260 10.3389/fimmu.2024.1335307 PMC11021656

[20] Oliveira F, Kamhawi S, Seitz AE, Pham VM, Guigal PM, Fischer L, From transcriptome to immunome: identification of DTH inducing proteins from a Phlebotomus ariasi salivary gland cDNA library. Vaccine. 2006;24(3):374–90. 16154670 10.1016/j.vaccine.2005.07.085

[21] Lerner EA, Ribeiro JM, Nelson RJ, and Lerner MR. Isolation of maxadilan, a potent vasodilatory peptide from the salivary glands of the sand fly Lutzomyia longipalpis. J Biol Chem. 1991;266(17):11234–6. 2040631

[22] Winer H, Rodrigues GOL, Hixon JA, Aiello FB, Hsu TC, Wachter BT, IL-7: Comprehensive review. Cytokine. 2022;160:156049. 36201890 10.1016/j.cyto.2022.156049

[23] Theodor O. A study of the reaction to phlebotomus bites with some remarks on “Harara”. Trans R Soc Trop Med Hyg. 1935;29(3):273–84.

[24] Alexander JO. Papular urticaria and immune complexes. J Am Acad Dermatol. 1985;12(2 Pt 1):374–5. 3973134 10.1016/s0190-9622(85)80065-7

[25] Strobl J, and Haniffa M. Functional heterogeneity of human skin-resident memory T cells in health and disease. Immunol Rev. 2023;316(1):104–19. 37144705 10.1111/imr.13213 PMC10952320

[26] Vogt MB, Lahon A, Arya RP, Kneubehl AR, Spencer Clinton JL, Paust S, and Rico-Hesse R. Mosquito saliva alone has profound effects on the human immune system. PLoS Negl Trop Dis. 2018;12(5):e0006439. 29771921 10.1371/journal.pntd.0006439 PMC5957326

[27] Ariotti S, Hogenbirk MA, Dijkgraaf FE, Visser LL, Hoekstra ME, Song JY, T cell memory. Skin-resident memory CD8(+) T cells trigger a state of tissue-wide pathogen alert. Science. 2014;346(6205):101–5. 25278612 10.1126/science.1254803

[28] Kadoki M, Patil A, Thaiss CC, Brooks DJ, Pandey S, Deep D, Organism-Level Analysis of Vaccination Reveals Networks of Protection across Tissues. Cell. 2017;171(2):398–413 e21. 28942919 10.1016/j.cell.2017.08.024 PMC7895295

[29] Gomes R, Teixeira C, Teixeira MJ, Oliveira F, Menezes MJ, Silva C, Immunity to a salivary protein of a sand fly vector protects against the fatal outcome of visceral leishmaniasis in a hamster model. Proc Natl Acad Sci U S A. 2008;105(22):7845–50. 18509051 10.1073/pnas.0712153105 PMC2397325

[30] Tavares NM, Silva RA, Costa DJ, Pitombo MA, Fukutani KF, Miranda JC, Lutzomyia longipalpis saliva or salivary protein LJM19 protects against Leishmania braziliensis and the saliva of its vector, Lutzomyia intermedia. PLoS Negl Trop Dis. 2011;5(5):e1169. 21655303 10.1371/journal.pntd.0001169 PMC3104964

[31] Asojo OA, Kelleher A, Liu Z, Pollet J, Hudspeth EM, Rezende WC, Structure of SALO, a leishmaniasis vaccine candidate from the sand fly Lutzomyia longipalpis. PLoS Negl Trop Dis. 2017;11(3):e0005374. 28278244 10.1371/journal.pntd.0005374 PMC5344329

[32] Ferreira VP, Fazito Vale V, Pangburn MK, Abdeladhim M, Mendes-Sousa AF, Coutinho-Abreu IV, SALO, a novel classical pathway complement inhibitor from saliva of the sand fly Lutzomyia longipalpis. Sci Rep. 2016;6:19300. 26758086 10.1038/srep19300 PMC4725370

[33] Collin N, Assumpcao TC, Mizurini DM, Gilmore DC, Dutra-Oliveira A, Kotsyfakis M, Lufaxin, a novel factor Xa inhibitor from the salivary gland of the sand fly Lutzomyia longipalpis blocks protease-activated receptor 2 activation and inhibits inflammation and thrombosis in vivo. Arterioscler Thromb Vasc Biol. 2012;32(9):2185–98. 22796577 10.1161/ATVBAHA.112.253906 PMC3421056

[34] Mendes-Sousa AF, do Vale VF, Silva NCS, Guimaraes-Costa AB, Pereira MH, Sant’Anna MRV, The Sand Fly Salivary Protein Lufaxin Inhibits the Early Steps of the Alternative Pathway of Complement by Direct Binding to the Proconvertase C3b-B. Front Immunol. 2017;8:1065. 28912782 10.3389/fimmu.2017.01065 PMC5583147

[35] Xu X, Oliveira F, Chang BW, Collin N, Gomes R, Teixeira C, Structure and function of a “yellow” protein from saliva of the sand fly Lutzomyia longipalpis that confers protective immunity against Leishmania major infection. J Biol Chem. 2011;286(37):32383–93. 21795673 10.1074/jbc.M111.268904 PMC3173228

[36] Cecilio P, Oristian J, Meneses C, Serafim TD, Valenzuela JG, Cordeiro da Silva A, and Oliveira F. Engineering a vector-based pan-Leishmania vaccine for humans: proof of principle. Sci Rep. 2020;10(1):18653. 33122717 10.1038/s41598-020-75410-0 PMC7596519

[37] Zundler S, and Neurath MF. Interleukin-12: Functional activities and implications for disease. Cytokine Growth Factor Rev. 2015;26(5):559–68. 26182974 10.1016/j.cytogfr.2015.07.003

[38] Schmitt E, Rude E, and Germann T. The immunostimulatory function of IL-12 in T-helper cell development and its regulation by TGF-beta, IFN-gamma and IL-4. Chem Immunol. 1997;68:70–85. 9329217 10.1159/000058695

[39] Castilho TM, Goldsmith-Pestana K, Lozano C, Valderrama L, Saravia NG, and McMahon-Pratt D. Murine model of chronic L. (Viannia) panamensis infection: role of IL-13 in disease. Eur J Immunol. 2010;40(10):2816–29. 20827674 10.1002/eji.201040384 PMC3289133

[40] Matthews DJ, Emson CL, McKenzie GJ, Jolin HE, Blackwell JM, and McKenzie AN. IL-13 is a susceptibility factor for Leishmania major infection. J Immunol. 2000;164(3):1458–62. 10640762 10.4049/jimmunol.164.3.1458

[41] Zaatar MT, Simaan Y, and Karam MC. Exogenous IL-13 exacerbates Leishmania major infection and abrogates acquired immunity to re-infection. Parasitol Res. 2022;121(7):2009–17. 35536514 10.1007/s00436-022-07539-y

[42] Alexander J, Brombacher F, McGachy HA, McKenzie AN, Walker W, and Carter KC. An essential role for IL-13 in maintaining a non-healing response following Leishmania mexicana infection. Eur J Immunol. 2002;32(10):2923–33. 12355446 10.1002/1521-4141(2002010)32:10<2923::AID-IMMU2923>3.0.CO;2-E

[43] Bourreau E, Prevot G, Pradinaud R, and Launois P. Interleukin (IL)-13 is the predominant Th2 cytokine in localized cutaneous leishmaniasis lesions and renders specific CD4+ T cells unresponsive to IL-12. J Infect Dis. 2001;183(6):953–9. 11237813 10.1086/319249

[44] Junior J, de Souza JL, da Silva LS, da Silva CC, do Nascimento TA, de Souza MLG, A fine mapping of single nucleotide variants and haplotype analysis of IL13 gene in patients with Leishmania guyanensis-cutaneous leishmaniasis and plasma cytokines IL-4, IL-5, and IL-13. Front Immunol. 2023;14:1232488. 37908348 10.3389/fimmu.2023.1232488 PMC10613733

[45] Oliveira F, Lawyer PG, Kamhawi S, and Valenzuela JG. Immunity to distinct sand fly salivary proteins primes the anti-Leishmania immune response towards protection or exacerbation of disease. PLoS Negl Trop Dis. 2008;2(4):e226. 18414648 10.1371/journal.pntd.0000226 PMC2291569

[46] Bretscher P. What Determines the Class of Immunity an Antigen Induces? A Foundational Question Whose Rational Consideration Has Been Undermined by the Information Overload. Biology (Basel). 2023;12(9). 10.3390/biology12091253 PMC10525557 37759652

[47] Murray JS. How the MHC selects Th1/Th2 immunity. Immunol Today. 1998;19(4):157–63. 9577091 10.1016/s0167-5699(97)01237-1

[48] Scheiblhofer S, Laimer J, Machado Y, Weiss R, and Thalhamer J. Influence of protein fold stability on immunogenicity and its implications for vaccine design. Expert Rev Vaccines. 2017;16(5):479–89. 28290225 10.1080/14760584.2017.1306441 PMC5490637

[49] Souissi C, Marzouki S, Elbini-Dhouib I, Jebali J, Oliveira F, Valenzuela JG, PpSP32, the Phlebotomus papatasi immunodominant salivary protein, exerts immunomodulatory effects on human monocytes, macrophages, and lymphocytes. Parasit Vectors. 2023;16(1):1. 36593519 10.1186/s13071-022-05627-7 PMC9806891

[50] Tanaka T, Narazaki M, and Kishimoto T. IL-6 in inflammation, immunity, and disease. Cold Spring Harb Perspect Biol. 2014;6(10):a016295. 25190079 10.1101/cshperspect.a016295 PMC4176007

[51] Ehrchen JM, Roebrock K, Foell D, Nippe N, von Stebut E, Weiss JM, Keratinocytes determine Th1 immunity during early experimental leishmaniasis. PLoS Pathog. 2010;6(4):e1000871. 20442861 10.1371/journal.ppat.1000871 PMC2861693

[52] Stager S, Maroof A, Zubairi S, Sanos SL, Kopf M, and Kaye PM. Distinct roles for IL-6 and IL-12p40 in mediating protection against Leishmania donovani and the expansion of IL-10+ CD4+ T cells. Eur J Immunol. 2006;36(7):1764–71. 16791879 10.1002/eji.200635937 PMC2659577

[53] Adachi T, Kobayashi T, Sugihara E, Yamada T, Ikuta K, Pittaluga S, Hair follicle-derived IL-7 and IL-15 mediate skin-resident memory T cell homeostasis and lymphoma. Nat Med. 2015;21(11):1272–9. 26479922 10.1038/nm.3962 PMC4636445

[54] Scott P. Long-Lived Skin-Resident Memory T Cells Contribute to Concomitant Immunity in Cutaneous Leishmaniasis. Cold Spring Harb Perspect Biol. 2020;12(10). 10.1101/cshperspect.a038059 PMC7528853 32839202

[55] Lima-Junior DS, Costa DL, Carregaro V, Cunha LD, Silva AL, Mineo TW, Inflammasome-derived IL-1beta production induces nitric oxide-mediated resistance to Leishmania. Nat Med. 2013;19(7):909–15. 23749230 10.1038/nm.3221

[56] Charmoy M, Hurrell BP, Romano A, Lee SH, Ribeiro-Gomes F, Riteau N, The Nlrp3 inflammasome, IL-1beta, and neutrophil recruitment are required for susceptibility to a nonhealing strain of Leishmania major in C57BL/6 mice. Eur J Immunol. 2016;46(4):897–911. 26689285 10.1002/eji.201546015 PMC4828310

[57] Dey R, Joshi AB, Oliveira F, Pereira L, Guimaraes-Costa AB, Serafim TD, Gut Microbes Egested during Bites of Infected Sand Flies Augment Severity of Leishmaniasis via Inflammasome-Derived IL-1beta. Cell Host Microbe. 2018;23(1):134–43 e6. 29290574 10.1016/j.chom.2017.12.002 PMC5832060

[58] Fernandez-Figueroa EA, Rangel-Escareno C, Espinosa-Mateos V, Carrillo-Sanchez K, Salaiza-Suazo N, Carrada-Figueroa G, Disease severity in patients infected with Leishmania mexicana relates to IL-1beta. PLoS Negl Trop Dis. 2012;6(5):e1533. 22629474 10.1371/journal.pntd.0001533 PMC3358333

[59] Novais FO, Carvalho AM, Clark ML, Carvalho LP, Beiting DP, Brodsky IE, CD8+ T cell cytotoxicity mediates pathology in the skin by inflammasome activation and IL-1beta production. PLoS Pathog. 2017;13(2):e1006196. 28192528 10.1371/journal.ppat.1006196 PMC5325592

[60] Santos D, Campos TM, Saldanha M, Oliveira SC, Nascimento M, Zamboni DS, IL-1beta Production by Intermediate Monocytes Is Associated with Immunopathology in Cutaneous Leishmaniasis. J Invest Dermatol. 2018;138(5):1107–15. 29246797 10.1016/j.jid.2017.11.029 PMC5912958

[61] Kumar R, Bunn PT, Singh SS, Ng SS, Montes de Oca M, De Labastida Rivera F, Type I Interferons Suppress Anti-parasitic Immunity and Can Be Targeted to Improve Treatment of Visceral Leishmaniasis. Cell Rep. 2020;30(8):2512–25 e9. 32101732 10.1016/j.celrep.2020.01.099 PMC7981274

[62] Xin L, Vargas-Inchaustegui DA, Raimer SS, Kelly BC, Hu J, Zhu L, Type I IFN receptor regulates neutrophil functions and innate immunity to Leishmania parasites. J Immunol. 2010;184(12):7047–56. 20483775 10.4049/jimmunol.0903273 PMC4159077

[63] Mattner J, Schindler H, Diefenbach A, Rollinghoff M, Gresser I, and Bogdan C. Regulation of type 2 nitric oxide synthase by type 1 interferons in macrophages infected with Leishmania major. Eur J Immunol. 2000;30(8):2257–67. 10940917 10.1002/1521-4141(2000)30:8<2257::AID-IMMU2257>3.0.CO;2-U

[64] Antonelli LR, Dutra WO, Oliveira RR, Torres KC, Guimaraes LH, Bacellar O, and Gollob KJ. Disparate immunoregulatory potentials for double-negative (CD4- CD8-) alpha beta and gamma delta T cells from human patients with cutaneous leishmaniasis. Infect Immun. 2006;74(11):6317–23. 16923794 10.1128/IAI.00890-06 PMC1695524

[65] Ferraz R, Cunha CF, Pimentel MIF, Lyra MR, Pereira-Da-Silva T, Schubach AO, CD3(+)CD4(neg)CD8(neg) (double negative) T lymphocytes and NKT cells as the main cytotoxic-related-CD107a(+) cells in lesions of cutaneous leishmaniasis caused by Leishmania (Viannia) braziliensis. Parasit Vectors. 2017;10(1):219. 28468680 10.1186/s13071-017-2152-2 PMC5415843

[66] Mou Z, Liu D, Okwor I, Jia P, Orihara K, and Uzonna JE. MHC class II restricted innate-like double negative T cells contribute to optimal primary and secondary immunity to Leishmania major. PLoS Pathog. 2014;10(9):e1004396. 25233487 10.1371/journal.ppat.1004396 PMC4169504

[67] Haddow AD, Curler G, and Moulton JK. New records of Lutzomyia shannoni and Lutzomyia vexator (Diptera: Psychodidae) in eastern Tennessee. J Vector Ecol. 2008;33(2):393–6. 19263861 10.3376/1081-1710-33.2.393

