# Human sand fly challenge elicits saliva-specific innate and T_H_1-polarized immunity that promotes *Leishmania* killing

**DOI:** 10.1101/2025.02.25.640210

**Published:** 2025-04-15

**Authors:** Maha Abdeladhim, Clarissa Teixeira, Roseanne Ressner, Kelly Hummer, Ranadhir Dey, Regis Gomes, Waldionê de Castro, Fernanda Fortes de Araujo, George W. Turiansky, Eva Iniguez, Claudio Meneses, Fabiano Oliveira, Naomi Aronson, Joshua R. Lacsina, Jesus G. Valenzuela, Shaden Kamhawi

**Affiliations:** 1 Vector Molecular Biology Section, Laboratory of Malaria and Vector Research, National Institute of Allergy and Infectious Diseases, National Institutes of Health, Rockville, Maryland, United States.; 2 Laboratory of Immunoparasitology, Department of Biotechnology, Oswaldo Cruz Foundation, Eusébio, Ceará, Brazil.; 3 Walter Reed National Military Medical Center, Bethesda, Maryland, United States.; 4 Center for Infectious Disease Research, Walter Reed Army Institute of Research, Silver Spring, Maryland, United States.; 5 Infectious Diseases Division, Department of Medicine, Uniformed Services University of the Health Sciences, Bethesda, Maryland, United States.; 6 Henry M Jackson Foundation for the Advancement of Military Medicine, Bethesda, Maryland, United States.; 7 Division of Emerging and Transfusion Transmitted Diseases, Office of Blood Research and Review, Center for Biologics Evaluation and Research, Food and Drug Administration, Silver Spring, Maryland, United States.; 8 Department of Dermatology, Uniformed Services University of the Health Sciences, Bethesda, Maryland, United States.

## Abstract

**Background:**

In *Leishmania*-endemic areas, humans are chronically exposed to the bites of vector sand flies, whose immunogenic salivary proteins modulate host immunity. The immune consequences of this chronic antigenic exposure remain poorly understood.

**Objective:**

To characterize bite-induced rash and cytokine responses to sand fly salivary proteins in humans chronically exposed to *Lutzomyia longipalpis*, the main vector of visceral leishmaniasis in the Americas.

**Methods:**

We conducted a controlled human vector challenge study, exposing 15 healthy volunteers to *Lutzomyia longipalpis* bites multiple times over a year. We assessed bite site skin reactions and stimulated peripheral blood mononuclear cells (PBMCs) with salivary gland extract or two immunogenic *Lutzomyia longipalpis* salivary proteins, LJM19 and LJL143, to measure cytokine responses and macrophage killing of *Leishmania*.

**Results:**

Bite sites developed pruritic, erythematous papules consistent with a delayed-type hypersensitivity (DTH) response. PBMCs stimulated with LJM19 or LJL143 elicited T_H_1-polarized cytokine responses and high expression of the T_H_2 cytokine IL-13. LJM19 increased levels of IL-6 and IL-7, while both proteins stimulated IL-1β and IFN-α production. LJM19- and LJL143-mediated parasite killing by macrophages correlated with T_H_1 polarization. LJM19 also enhanced *Leishmania* killing in cells from individuals unexposed to *Lutzomyia longipalpis*. Two participants exhibited prominent bite site infiltration with CD4-CD8- T cells.

**Conclusions:**

Chronic exposure to sand fly bites elicits a DTH response and induces innate and adaptive cytokine responses to salivary proteins that correlate with enhanced *Leishmania* killing. Controlled human vector challenge studies are valuable for investigating the immunologic mechanisms driven by long-term exposure to vector salivary antigens.

## INTRODUCTION

Leishmaniasis is a neglected vector-borne disease caused by protozoan parasites of the genus *Leishmania*, which are transmitted via the bite of phlebotomine sand flies. Each year, over 700,000 new cases of leishmaniasis are reported worldwide.^1^

Sand fly saliva plays a critical role in *Leishmania* transmission. When female sand flies bite their host to take a blood meal, they inject a complex mixture of immunogenic, biologically active salivary proteins into the skin. These foreign salivary antigens trigger a host immune response that favors parasite establishment.^2^ In *Leishmania*-endemic areas, humans are constantly exposed to these salivary antigens, which induce durable cytokine responses from peripheral blood mononuclear cells (PBMCs). These cytokine responses can be recalled up to ten years after the most recent sand fly exposure.^3, 4^ Similarly, in the skin, the ability of sand fly bites to elicit a delayed-type hypersensitivity (DTH) response can persist for decades in humans with ongoing sand fly exposure.^5^ In patients with CL, cellular and antibody responses to certain sand fly salivary proteins strongly correlate with CL lesion size and whether the disease is localized or disseminated.^6, 7^ Thus, clinical studies are needed to characterize more fully the effects of chronic sand fly exposure on human immunity, particularly responses to sand fly saliva and salivary proteins.

Human immunity to sand fly saliva has been investigated through human vector challenge studies, where healthy volunteers are exposed to the bites of uninfected sand flies. Although only a few such human challenge studies have been conducted, they demonstrate that most volunteers develop a skin DTH response to sand fly bites and a mixed cellular T_H_1/T_H_2 cytokine response when their PBMCs are stimulated with salivary gland extract (SGE) *ex vivo*.^4, 5, 8^ A recent human challenge study extended these findings by identifying individual salivary proteins from the Afro-Asian sand fly *Phlebotomus duboscqi* that induce strong IFN-γ or IL-10 responses in PBMCs.^8^

In this human sand fly challenge study, we investigated human cellular immunity to the saliva of the sand fly *Lutzomyia longipalpis* (*Lu. longipalpis*), the principal vector of visceral leishmaniasis in the Americas. We characterized these immune responses by challenging individuals with uninfected *Lu. longipalpis* bites multiple times over nearly a year, simulating the chronic immune exposure to sand fly saliva that occurs in *Leishmania*-endemic populations. We identified two sand fly salivary proteins that elicit robust innate and T_H_1-polarized cytokine responses in human immune cells and enhance killing of *Leishmania* parasites *ex vivo*.

## METHODS

### Study approval

The Institutional Review Boards of Walter Reed Army Medical Center, Walter Reed National Military Medical Center (protocol number WR355023), the National Institute of Allergy and Infectious Diseases (NIAID), and the Uniformed Services University of the Health Sciences (USUHS) approved this study. All human subjects research was conducted in accordance with the principles of the Declaration of Helsinki. Participants provided written informed consent prior to study participation, including consent for the use of their photographs. The study was registered on www.clinicaltrials.gov as NCT01289977. Details about the study population, inclusion, and exclusion criteria are provided in the Methods section of the Online Repository.

### Additional methods

The Methods section in the Online Repository provides detailed protocols for: controlled human exposure to *Lu. longipalpis* sand flies, skin biopsy histology and immunohistochemistry (IHC), quantitative RT-PCR, preparation of sand fly salivary gland extract (SGE), production of recombinant *Lu. longipalpis* salivary proteins, PBMC culture and stimulation, cytokine measurement, macrophage parasite killing assay, and statistical analysis.

Differences were considered statistically significant for p < 0.05 (*). Due to the exploratory nature of our study, we also indicated where p > 0.05 but < 0.10 (denoted by “#”) to identify potential trends that may be biologically relevant but did not achieve the significance threshold.

## RESULTS

### Characteristics of study participants

**FIG 1.**
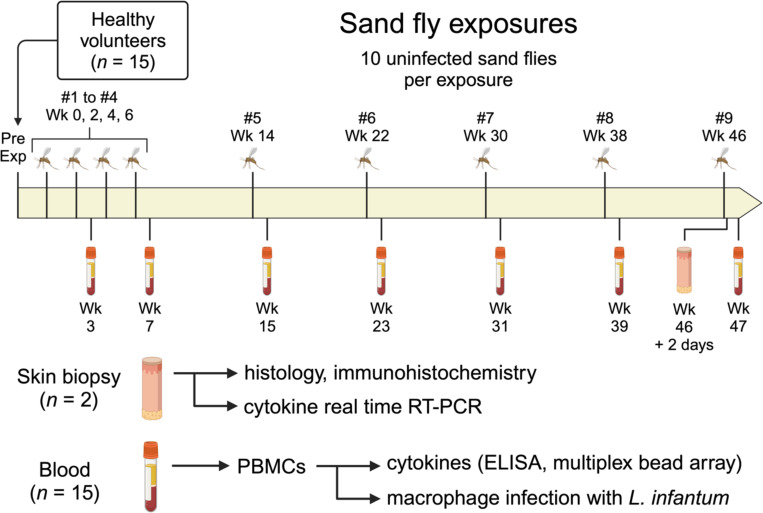
Schematic of clinical study. Healthy volunteers (*n* = 15) were exposed to the bites of uninfected *Lutzomyia longipalpis* sand flies once every two weeks for exposures #1 through #4, then once every eight weeks for exposures #5 to #9. Blood was collected ~1 week after exposures #2, #4, and #5 to #9, followed by PBMC isolation to perform the indicated assays. Skin punch biopsies were obtained from two participants 48 hours after exposure #9, with one biopsy from a sand fly bite site and one biopsy from the contralateral arm for each participant. *Pre Exp*, pre-exposure study visit. Created in BioRender.com.

Table I presents the demographics, allergy history, and baseline plasma IgE levels of the study participants. Fifteen healthy volunteers were enrolled in the study. The median age was 27 years (range 22 to 46), and the majority were male (12 of 15, 80%). Figure 1 provides a schematic of the study design. Loss to follow-up began at exposure #6 (Fig E1), primarily due to active duty military participants being transferred out of the study area (*n* = 4) and one participant being discharged from the military (Participant #3). Three participants halted their participation due to study-related reactions to sand fly bites, which included large areas of urticaria, pruritus, and erythema, raising concerns about potentially worse symptoms with future sand fly exposures. The number of fed sand flies was consistent across all feedings, with an average of 8 to 9 fully fed and 0 to 1 partially fed sand flies out of 10 total per participant at each exposure.

### Progressive exposures to *Lu. longipalpis* alter the bite site rash phenotype

**FIG 2.**
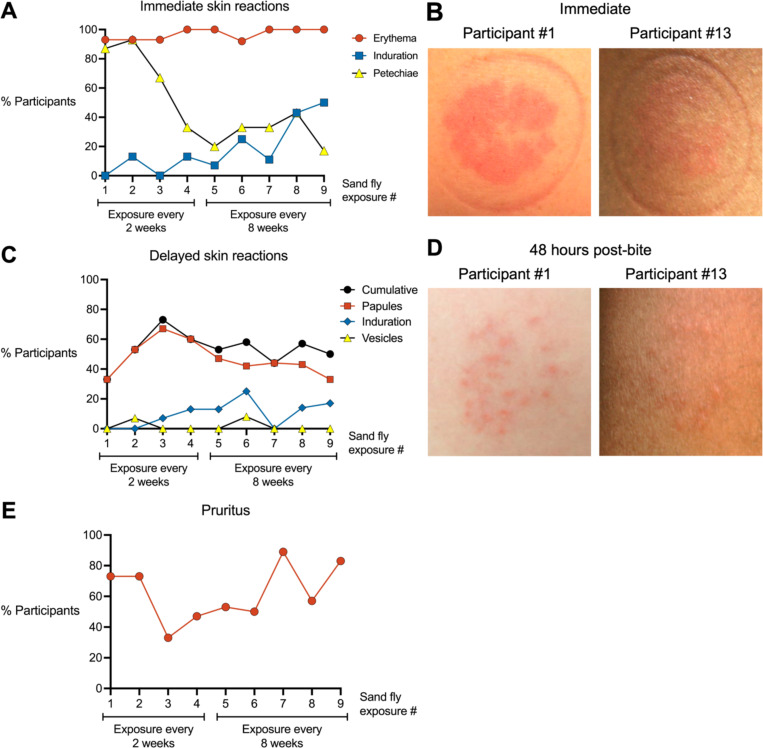
Characteristics of rashes at the sites of uninfected *Lutzomyia longipalpis* bites. (**A**) Skin reactions observed within the first 10 minutes after completion of each sand fly exposure. (**B**) Representative photographs from two participants immediately after Exposure #9. (**C**) Delayed skin reactions at the bite site that developed one or more days after completion of each sand fly exposure. (**D**) Representative photographs from two participants 48 hours after Exposure #9. (**E**) Pruritus at the bite site.

We characterized the phenotype and symptoms of rashes induced by uninfected *Lu. longipalpis* bites across multiple sand fly exposures. For immediate bite site reactions, 85% to 100% of participants exhibited erythema across all exposures (Fig 2, A and B), consistent with the potent vasodilatory activity of the *Lu. longipalpis* salivary protein maxadilan.^9^ Petechiae decreased with successive exposures, with only one of six participants exhibiting petechiae at the final exposure. Immediate induration was initially absent but increased to 50% of participants by the final exposure.

We next evaluated delayed bite site reactions that developed hours to one week after each sand fly exposure (Fig 2, C and D). Papules were the most frequent delayed rash phenotype (Fig 2, C), increasing from 33% to 67% of participants over the first three exposures before declining to 33% by the final exposure. Induration and vesicles appeared inconsistently in a maximum of 23% and 8% of participants, respectively. Localized pruritus at the bite site (either delayed or immediate) was initially reported by 73% of participants and increased to 83% at the final exposure (Fig 2, E). Across all exposures, the median pruritus duration was 2 days (range 30 minutes to 3 months).

Interestingly, for 8 of 15 participants, sand fly exposure triggered the appearance of a rash at a previous bite site on the contralateral arm, which had been exposed weeks before (median 6.5 weeks prior, range 2 to 11 weeks). This phenomenon, termed distal bite site reactivation, manifested approximately one week after the most recent sand fly exposure (median 4.5 exposures, range 2 to 9).

### *Lu. longipalpis* bites induce a delayed-type hypersensitivity skin response after multiple sand fly exposures

Forty-eight hours after the final sand fly exposure, two participants (#1 and #13) who completed all nine exposures volunteered for skin biopsies from a bite site and from the contralateral arm as a negative control. Both participants exhibited multiple erythematous papules at their exposure site, characteristic of a DTH response (Fig 2, D).

**FIG 3.**
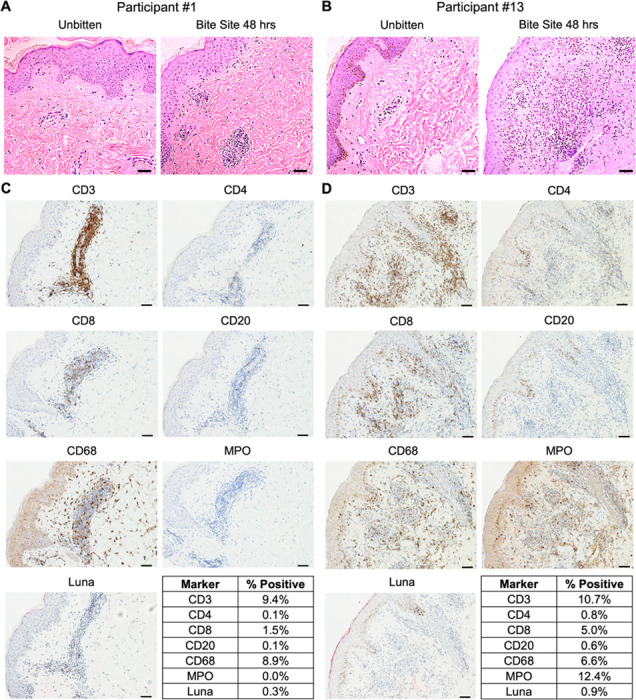
Repeated exposures to uninfected *Lu. longipalpis* bites induce a delayed-type hypersensitivity response at the bite site. Skin punch biopsies were collected from bite site skin and from normal appearing skin on the contralateral arm from two participants 48 hours after exposure #9. (**A** and **B**) Hematoxylin and eosin stains. (**C** and **D**) Immunohistochemistry (IHC) of the indicated markers at the bite site. Tables show the percentage of cells that stained positive for each marker based on analysis with ImageJ. Scale bar is 50 μm for all images.

Histology from Participant #1 showed spongiosis and a perivascular mononuclear (lymphocytic and histiocytic) infiltrate with occasional eosinophils (Fig 3, A). Participant #13 exhibited a diffuse, mixed inflammatory infiltrate with both neutrophils and mononuclear cells present in the upper dermis, distributed throughout the perivascular, interstitial, and perieccrine areas (Fig 3, B).

Immunohistochemistry (IHC) revealed that the infiltrate in both participants was largely composed of T cells (CD3) and macrophages (CD68), while B cells (CD20) and eosinophils (Luna) were rare or absent, respectively (Fig 3, C and D). Interestingly, the CD4 signal was minimal (Participant #13) to absent (Participant #1), while the CD8 signal overlapped with a proportion of the CD3 staining pattern. Thus, the CD4 and CD8 signals do not fully account for the strong CD3 signal observed in both individuals. While Participant #1 had no detectable neutrophils by myeloperoxidase (MPO) stain (Fig 3, C), Participant #13 showed MPO staining throughout the dermis (Fig 3, D). These data highlight macrophages and T cells as the predominant immune cells driving the DTH response to *Lu. longipalpis* bites in these two individuals. The IHC data also suggest that CD4^−^CD8^−^ T cells (also termed double negative or DN T cells) may play a role in the DTH response to sand fly bites.

Quantitative RT-PCR of the skin biopsies elucidated the cutaneous T_H_ cytokine profile at the bite site (Fig E2). Participant #1 exhibited a mixed T_H_1/T_H_2 response with similar levels of IFN-γ and IL-13, and only minor contributions from IL-12 and IL-5. In contrast, Participant #13 showed a dominant T_H_1 response driven by high expression of IFN-γ, no detectable IL-13, and comparatively low expression of IL-12 and IL-5.

### *Lu. longipalpis* bites induce cellular interferon gamma responses to the sand fly salivary proteins LJL143 and LJM19

**FIG 4.**
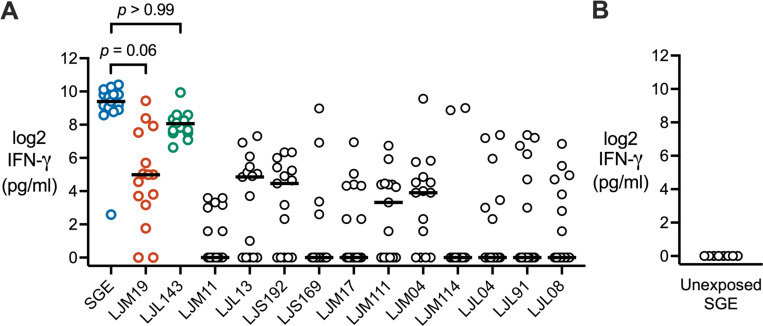
Recombinant salivary proteins stimulate IFN-γ production in PBMCs from *Lu. longipalpis*-exposed volunteers. (**A**) PBMCs were stimulated with salivary gland extract (SGE) or the indicated recombinant *Lu. longipalpis* salivary protein to measure IFN-γ levels by ELISA. PBMCs were from exposure #2 or #4 with one time point per volunteer (*n* = 15). *P*-values are shown only for comparisons with *p* > 0.05 that showed no significant difference from SGE. (**B**) As a negative control, IFN-γ was measured in SGE-stimulated PBMCs from healthy volunteers unexposed to *Lu. longipalpis* (*n* = 8). *P*-values were calculated by Friedman test with Dunn’s multiple comparisons test.

In preclinical models, repeated exposure to sand fly bites leads to a strong IFN-γ response to subsequent sand fly challenge, with IFN-γ being a correlate of protection against leishmaniasis.^10, 11^ To identify specific *Lu. longipalpis* salivary proteins that induce a strong cellular IFN-γ response, we screened the thirteen most abundant proteins in *Lu. longipalpis* saliva for their ability to stimulate IFN-γ production from PBMCs collected after the second or fourth sand fly exposure (Fig 4, A). LJL143 and LJM19 were the only salivary proteins that stimulated IFN-γ production at a level comparable to salivary gland extract (SGE), a standard for immunogenicity. Stimulation with any of the other eleven *Lu. longipalpis* salivary proteins resulted in significantly less IFN-γ production (all *p* < 0.01). As a negative control, no IFN-γ production was elicited by SGE treatment of PBMCs from *Lu. longipalpis*-unexposed individuals obtained from the NIH Blood Bank (Fig 4, B). These results suggest that repeated exposure of humans to *Lu. longipalpis* bites leads to the development of a robust cellular IFN-γ response to sand fly saliva and to the salivary proteins LJM19 and LJL143.

### LJM19 and LJL143 induce a T_H_1-polarized cytokine response

**FIG 5.**
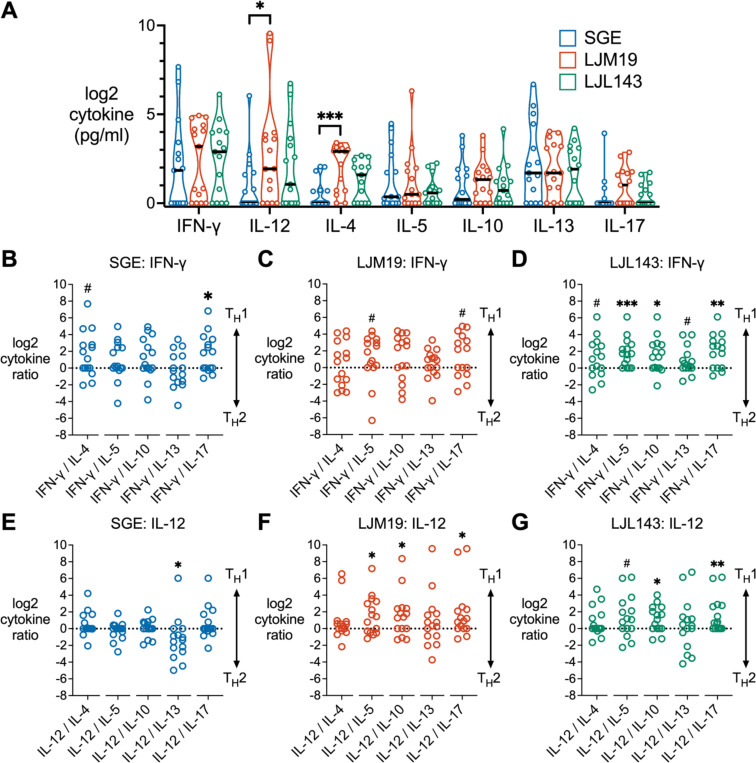
LJM19 and LJL143 induce T_H_1-polarized cytokine responses in PBMCs from *Lu. longipalpis*-exposed volunteers. (**A**) PBMCs obtained from sand fly-exposed study participants (*n* = 15) were stimulated with SGE, LJM19, or LJL143. Cell supernatants were collected at 96 hours and cytokine concentrations were measured by multiplex bead array. Black bar indicates the median. For each cytokine, differences between treatment groups were analyzed by Friedman test with Dunn’s test for multiple comparisons. PBMCs were from exposure #4 to #9 with one time point per participant. Ratios of T_H_1 to T_H_2 or T_H_17 cytokines were calculated for PBMCs treated with SGE (**B** and **E**), LJM19 (**C** and **F**), or LJL143 (**D** and **G**). Ratios above 0 indicate a T_H_1-polarized response while ratios below 0 indicate a T_H_2- or T_H_17-polarized response, as analyzed by Wilcoxon signed-rank test using a value of zero as the null hypothesis (equal balance of T_H_1 and T_H_2/T_H_17 cytokines), # *p* < 0.10, * *p* < 0.05, ** *p* < 0.01, *** *p* < 0.001.

We sought to determine how LJM19 and LJL143 stimulation affects the overall T_H_ polarization profile. PBMCs collected after exposures four through nine were stimulated with SGE, LJM19, or LJL143, and cytokines were measured in the supernatants by multiplex bead array. Comparing the three stimulation conditions for each T_H_ cytokine, we found that LJM19 induced significantly higher levels of IL-12 and IL-4 compared to SGE (Fig 5, A). Notably the T_H_2 cytokine IL-13 was consistently highly expressed across all three treatments.

To quantify T_H_ polarization, we calculated the ratios of the T_H_1 cytokines (IFN-γ and IL-12) against each T_H_2 cytokine (IL-4, IL-5, IL-10, and IL-13) and the T_H_17 cytokine, IL-17. SGE induced a mixed T_H_1/T_H_2 response with only a trend towards T_H_1 seen for the IFN-γ/IL-4 ratio (*p* = 0.05) (Fig 5, B). LJM19 treatment also induced a comparatively weak IFN-γ response, with a trend towards T_H_1 seen only for IFN-γ/IL-5 (Fig 5, C). LJL143 induced significant T_H_1 polarization via IFN-γ relative to IL-5 and IL-10, with a trend towards T_H_1 for IFN-γ versus IL-4 or IL-13 (Fig 5, D). For IL-12, SGE induced significant T_H_2 polarization relative to IL-13 (Fig 5, E). In contrast, LJM19-treated PBMCs exhibited significant T_H_1 polarization with IL-12 relative to the T_H_2 cytokines IL-5 and IL-10 (Fig 5, F). For LJL143, IL-12 also showed significant T_H_1 polarization versus IL-10 and a trend versus IL-5 (Fig 5, G). None of the treatments induced a T_H_17-polarized response. For each individual, the ratios were largely consistent across all T_H_1/T_H_2 cytokine pairs, except for IL-13, which was consistently expressed at a higher level than the other T_H_2 cytokines (Fig E3). In summary, LJL143 and LJM19 induce a T_H_1-polarized response in PBMCs from individuals with multiple *Lu. longipalpis* exposures. Notably, this T_H_1 response is observed primarily through IFN-γ for LJL143 and through IL-12 for LJM19 and is accompanied by high IL-13 expression.

### LJM19 and LJL143 induce innate inflammatory and antiviral cytokines

**FIG 6.**
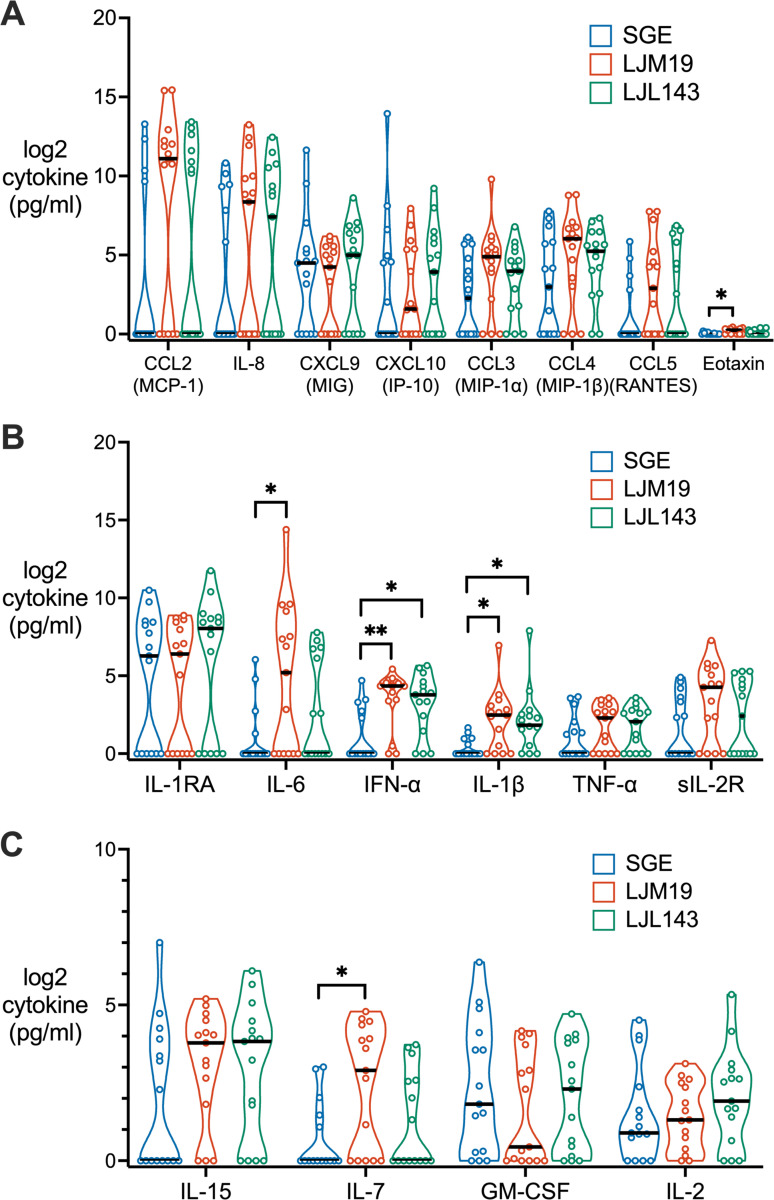
Differences in PBMC cytokine profiles induced by LJM19 or LJL143 compared to SGE. PBMCs obtained from *Lu. longipalpis*-exposed study participants (*n* = 15) were stimulated with SGE, LJM19, or LJL143. Cell supernatants were collected at 96 hours and cytokine concentrations were measured by multiplex bead array for chemokines (**A**), inflammatory cytokines (**B**), and cytokines promoting cell survival, activation, and proliferation (**C**). PBMCs used were from exposure #4 to #9 with one time point per participant. Black bar indicates the median. For each cytokine, differences between treatment groups were analyzed by Friedman test with Dunn’s test for multiple comparisons, * *p* < 0.05, ** *p* < 0.01.

We investigated the effects of SGE, LJM19, and LJL143 on other functional classes of cytokines. Among chemokines, LJM19 induced a significantly higher level of eotaxin than SGE; however, the eotaxin concentration produced by all treatment groups was negligible relative to the levels of other chemokines (Fig 6, A). Compared to SGE, both LJM19 and LJL143 induced significantly higher levels of interferon alpha (IFN-α), an antiviral and immunomodulatory cytokine, and IL-1β, an inflammatory cytokine (Fig 6, B). Additionally, LJM19 induced higher levels of IL-6 (Fig 6, B), an inflammatory cytokine, and IL-7 (Fig 6, C), which promotes lymphocyte proliferation and maintenance in peripheral tissues.^12^

### LJM19 and LJL143 enhance the ability of PBMCs to stimulate macrophage killing of intracellular *Leishmania* parasites

**FIG 7.**
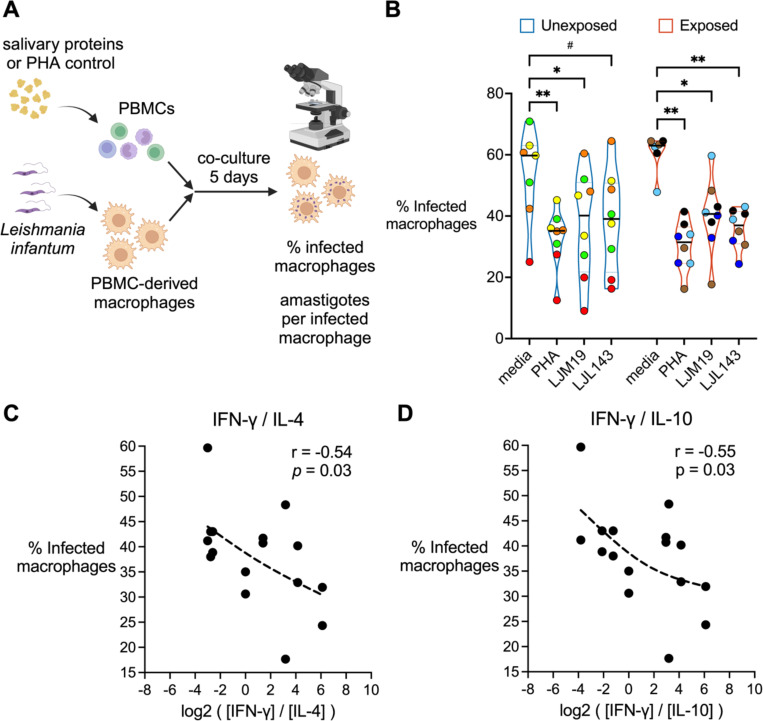
Stimulation of PBMCs with LJM19 or LJL143 enhances killing of *Leishmania* by macrophages. PBMC-derived macrophages from participants unexposed (*n* = 4, blue border) or exposed (*n* = 4, red border) to *Lu. longipalpis* were infected with *Leishmania infantum*, then co-cultured with autologous PBMCs that had been stimulated according to the conditions shown. After 5 days of co-culture, the percentage of infected macrophages was quantified by manual counting of Giemsa-stained cells by light microscopy. PBMCs were from exposure #7 to #9 with one time point per participant. (**A**) Experimental scheme. (**B**) Percentage of infected macrophages. Each volunteer’s batch of PBMCs was divided and run in two technical replicates; identical colors denote replicates from the same individual. Bar indicates the median. Differences between treatment groups were analyzed via a mixed effects model with Dunnett’s test for multiple comparisons, # *p* < 0.10, * *p* < 0.05, and ** *p* < 0.01. (**C** and **D**) Spearman correlation between the IFN-γ/IL-4 ratio (**C**) or IFN-γ/IL-10 ratio (**D**) as calculated in Figure 5 and the percentage of infected macrophages for LJM19- and LJL143-treated samples. Dashed line in **C** and **D** is the trend line (smoothing spline). Schematic in **A** created in BioRender.com.

We tested the hypothesis that treatment with LJM19 or LJL143 enhances the ability of PBMCs to stimulate the killing of intracellular parasites by *Leishmania*-infected macrophages. We compared PBMCs from *Lu. longipalpis*-naïve individuals from the NIH Blood Bank (Unexposed) to PBMCs from sand fly-exposed volunteers. For the latter group, we used PBMCs collected after exposures #7 through #9 from a subset of four participants who completed all nine scheduled sand fly exposures. Monocyte-derived macrophages were infected with *Leishmania infantum* and then co-cultured with autologous PBMCs stimulated with the sand fly salivary proteins LJM19 or LJL143. Phytohemagglutinin (PHA), a T cell mitogen, served as a positive control (Fig 7, A).

For *Lu. longipalpis*-exposed participants, PBMC stimulation with either LJM19 or LJL143 led to a significant reduction in the percentage of infected macrophages (Fig 7, B). This reduction was comparable in magnitude to that observed with PHA stimulation. Surprisingly, in PBMCs from *Lu. longipalpis*-unexposed individuals, stimulation with LJM19 alone also induced a significant decrease in the percentage of infected macrophages, while stimulation with LJL143 showed a trend towards a decrease. There were no significant differences between media alone and any of the treatment groups in the number of amastigotes per infected macrophage (Fig E4). Cross-referencing these macrophage infection data with our earlier T_H_ cytokine ratio results revealed a significant negative correlation between the percentage of infected macrophages and the ratios of IFN-γ/IL-4 and IFN-γ/IL-10 (Fig 7, C and D). No significant correlation was seen with IFN-γ alone, IFN-γ/IL-5, or IFN-γ/IL-13 (Fig E5).

## DISCUSSION

This study provides the most comprehensive characterization to date of how human skin and systemic immune responses evolve with cumulative exposure to the bites of *Lutzomyia longipalpis* sand flies. Stable DTH reactions to *Lu. longipalpis* bites developed and were maintained throughout the study, similar to a related human sand fly challenge study using the sand fly *Phlebotomus duboscqi*,^8^ and comparable to the durable DTH responses seen in a cohort in Mali after decades of exposure to *Phlebotomus duboscqi*.^5^

In preclinical models, saliva-specific T_H_1-polarized DTH responses are closely correlated with immune protection against *Leishmania*.^11, 13^ In our study, skin biopsies of the bite site from two participants who completed all nine sand fly exposures exhibited variable DTH reactions that were either T_H_1-polarized or exhibited a mixed T_H_1/T_H_2 response. For both volunteers, the site of the DTH response was composed of T cells, macrophages, and in one participant, neutrophils. Both biopsy sites were dominated by CD3+ CD4– CD8– T cells, also known as double negative (DN) T cells. A similar immunohistochemical pattern suggestive of DN T cells was observed in DTH reactions to *P. duboscqi* bites in individuals from a CL-endemic region in Mali,^5^ but not in individuals from a non-endemic area.^8^ While the role of DN T cells in the response to sand fly bites remains unclear, expanded DN T cell populations have been demonstrated in human CL lesions due to *L. braziliensis* and exhibit a highly activated phenotype.^14, 15^ These DN T cells express an αβ T cell receptor (TCR) and show high expression of IFN-γ and CD69, a tissue residence marker.^14^ In mice, DN T cells are necessary for primary and secondary immunity to *L. major*.^16^ Further studies are needed to investigate the dominant T_H_ polarization profile at the sand fly bite site, whether DN T cells are a consistent and prominent feature of the DTH response, and how sand fly saliva-primed T_H_ and DN T cells influence immunity to *Leishmania* parasites.

In over half of participants, *Lu. longipalpis* bites triggered the reappearance of a rash at a previously bitten site on the contralateral arm. This phenomenon, known as distal bite site reactivation, was also reported after uninfected *Phlebotomus duboscqi* bites^8^ and during early studies of human skin reactions to the bites of *Phlebotomus papatasi*.^17^ We speculate that this reactivation reflects the activation of salivary protein-specific, skin resident memory T cells that have seeded a prior bite site and respond to circulating salivary antigens from new sand fly bites.^18, 19^ The ability of vector bites to impact systemic immunity was demonstrated in humanized mice exposed to uninfected *Aedes aegypti* bites.^20^ These mice exhibited significant changes in blood cytokine levels and immune cell composition in both blood and skin up to seven days after mosquito exposure. This suggests the intriguing possibility that local cutaneous responses to vector bites may trigger systemic signals that promote a pathogen-resistant state throughout the skin, similar to the organism-wide coordination of antiviral immunity.^21, 22^

Using PBMCs, our screen identified LJM19 and LJL143 as the two *Lu. longipalpis* salivary proteins that stimulate the highest IFN-γ production in PBMCs from sand fly exposed participants, confirming their immunogenicity in humans. LJM19 (also named SALO) is an 11 kDa protein that inhibits the classical complement pathway and has no structural similarity to human proteins.^23, 24^ LJM19 was previously identified as a salivary protein vaccine candidate that protects against both cutaneous and visceral leishmaniasis in preclinical models.^25–28^ LJL143 (also named Lufaxin) is an inhibitor of coagulation factor Xa and the alternative pathway of complement.^29, 30^ In dogs, LJL143 elicits strong DTH and IFN-γ responses in the skin and blood following uninfected sand fly challenge.^31^ LJL143 exhibits adjuvant-like activity in mice, where priming immunized animals with unadjuvanted LJL143 induced higher CD4+ T cell proliferative responses to *Leishmania* antigens in a virus-like particle (VLP) vaccine compared to mice that did not receive LJL143.^25^ Despite this immunogenicity, LJL143 has shown only partial or no protection in mice challenged with *L. major* co-inoculated with saliva^32^ or infected sand fly bite.^33^ However, our data from human PBMCs show that LJL143 promotes the killing of *L. infantum* by infected macrophages *ex vivo*. This contrast highlights the importance of assessing these salivary proteins in a human immune context, where their activity may differ markedly from what is observed in preclinical models.

In participants exposed to *Lu. longipalpis*, treatment of PBMCs with SGE induced a mixed T_H_1/T_H_2 response, while LJM19 and LJL143 both induced T_H_1 responses. LJM19 was characterized by high IL-12 expression, whereas LJL143 showed high expression of both IFN-γ and IL-12. Classically, IL-12 production by antigen presenting cells leads to IFN-γ production by T cells.^34^ However, as seen with LJM19, strong induction of IL-4 can suppress IFN-γ despite high levels of IL-12.^35^ This co-expression of IL-12 and IL-4 mirrors the cytokine profile seen in LJM19-immunized hamsters protected against *L. donovani* challenge,^26^ suggesting that protection from leishmaniasis is not solely dependent on IFN-γ production but rather on the balance of T_H_ cytokines.

Of the T_H_2 cytokines, IL-13 was often expressed at levels equal to or higher than IFN-γ. IL-13 promotes *Leishmania* infection and exacerbates immunopathology,^36–38^ in part by inhibiting expression of IL12Rβ2 which transduces critical signals for T_H_1 differentiation.^39, 40^ Polymorphisms at the *IL13* locus in humans have also been associated with susceptibility to CL caused by *Leishmania guyanensis*.^41^ In a naturally exposed population in Mali, IL-13 was highly expressed in PBMCs stimulated with SGE from *P. duboscqi*, but only at low levels in the skin after uninfected bite challenge.^5^ Further studies are needed to determine whether the high IL-13 expression induced by LJM19 and LJL143 reflects an intrinsic property of these proteins or a counterregulatory response to T_H_1 polarization.

We found that LJM19 induces IL-6 and IL-7, while both LJM19 and LJL143 induce IL-1β and the type I interferon, IFN-α. This contrasts with the reported downregulation of IL-6 and IL-1β by PpSP32, another sand fly salivary protein.^42^ To our knowledge, this is the first study to report altered expression of IL-7 and IFN-α in response to specific sand fly salivary proteins. IL-6 exerts pleiotropic immune effects, including activation of the acute phase response, granulopoiesis, B cell proliferation, and CD8+ T effector cell development.^43^ In mice, IL-6 has been shown to facilitate resistance to *L. major*^44^ and *L. donovani*^45^, in the latter case by inhibiting the proliferation of IL-10-expressing CD4+ T cells. IL-7 supports the survival, proliferation, and maintenance of T cells in peripheral tissues,^12^ including skin resident memory T cells,^46^ which are critical for sustained protection against *Leishmania*.^47^ While IL-1β has been reported to protect against *L. amazonensis*,^48^ most studies suggest IL-1β exacerbates parasite dissemination and immunopathology.^49–53^ Similarly, IFN-α likely benefits the parasite by antagonizing IFN-γ responses and suppressing the development of *Leishmania*-specific T cells,^54, 55^ though the timing of IFN-α signaling relative to *Leishmania* infection can shift the balance between protection and susceptibility.^56^

Unexpectedly, even in individuals unexposed to *Lu. longipalpis*, treatment of PBMCs with LJM19 or LJL143 decreased the percentage of infected macrophages in co-culture. This suggests that LJM19 and LJL143 have intrinsic adjuvant-like properties in addition to their induction of robust adaptive immune responses. Identifying the innate immune receptors these proteins bind and the cell subsets mediating their adjuvant-like effects remains an area for future research. The similar number of amastigotes per infected macrophage between media-treated versus LJM19 or LJL143-treated samples suggests that these salivary proteins act early in macrophage infection, either by blocking parasite invasion or by enhancing killing of *Leishmania* shortly after invasion, before the parasite establishes in the phagolysosome.

Our study has several limitations. The cohort is small and demographically homogenous (80% male, 73% White), limiting generalizability to CL-endemic populations. Due to limited PBMC recovery at some blood collections, each assay used PBMCs from an early or late range of time points, though within each assay, only one time point was used per participant. The macrophage parasite killing assay could only be performed on a minority of the cohort (4 of 15 total, and 4 of 6 who completed all nine sand fly exposures). While we cannot exclude the possibility that participants were previously exposed to local *Lutzomyia* species at low prevalence,^57^ all participants and blood bank samples were screened and found to be negative for IgG against *Lu. longipalpis* SGE. Significant study attrition occurred, with 9 of 15 participants failing to complete all scheduled sand fly exposures. However, only 3 of the dropouts were study related, and all participants completed at least 5 exposures over 14 weeks, which is likely sufficient to develop adaptive immunity to sand fly salivary antigens. Finally, most of our findings are based on PBMC responses and need reinforcement through studies of immune cells in the skin.

In summary, we report that humans exposed to *Lu. longipalpis* generate robust innate and adaptive cellular immune responses to the sand fly salivary proteins LJM19 and LJL143. These proteins exhibit adjuvant-like activity, induce T_H_1 polarization, and enhance the protection of macrophages against *Leishmania* infection. This highlights the potential of leveraging anti-saliva immunity in humans to protect against vector borne pathogens. Furthermore, our work underscores the power of vector human challenge studies to elucidate how chronic exposure to vector salivary antigens impacts human immunity, a daily occurrence for the approximately 350 million people who live in regions where sand flies and other disease vectors are prevalent.

## Supplementary Material

Supplement 1

## Figures and Tables

**TABLE I.** Study participant characteristics.

Participant ID	Completed Sand Fly Exposure #	Age	Sex	Race	Allergy History	Baseline Plasma IgE (kU/L)	Reason for Early Withdrawal or Skipped Feedings
1	9	26	M	White	None	17.9	-
2	5	26	M	White	None	9.9	Military transfer orders
3	6	26	M	Asian	Seasonal allergies Asthma	114.0	Discharged from military
4	8	40	M	White	Asthma	6.7	Military transfer orders
5	7	29	M	White	None	3.3	Military transfer orders
6	6	34	M	White	None	19.9	Participant request, adverse event
7	6	45	M	Black	Seasonal allergies Allergic to shellfish Eczema Asthma	20.7	Participant request, adverse event
8	9	22	M	White	None	96.6	-
9	5	26	M	Asian	Seasonal allergies	6.4	Medically advised to stop due to adverse event
10	5	24	M	White	Anaphylaxis to raisins	15.3	Military transfer orders
11	7	46	M	White	Seasonal allergies	<2.0	Two feedings skipped (medical hold, schedule conflict)
12	9	27	F	White	Urticaria to acetaminophen-hydrocodone Bee allergy Asthma	26.0	-
13	9	26	F	Multiracial	Seasonal allergies Angioedema to shellfish	61.1	-
14	9	41	M	White	Seasonal allergies Asthma	77.2	-
15	9	28	F	White	Urticaria to amoxicillin-clavulanic acid	6.7	-

## References

[1] BDCN. Global Burden of Disease Study 2021 (GBD 2021). In: IHME, ed. Seattle, WA, United States, 2024.

[2] Serafim TD, Coutinho-Abreu IV, Dey R, Kissinger R, Valenzuela JG, Oliveira F, Kamhawi S. Leishmaniasis: the act of transmission. Trends Parasitol 2021; 37:976–87. 34389215 10.1016/j.pt.2021.07.003

[3] Lakhal-Naouar I, Mukbel R, DeFraites RF, Mody RM, Massoud LN, Shaw D, The human immune response to saliva of Phlebotomus alexandri, the vector of visceral leishmaniasis in Iraq, and its relationship to sand fly exposure and infection. PLoS Negl Trop Dis 2021; 15:e0009378. 34081700 10.1371/journal.pntd.0009378 PMC8174707

[4] Vinhas V, Andrade BB, Paes F, Bomura A, Clarencio J, Miranda JC, Human anti-saliva immune response following experimental exposure to the visceral leishmaniasis vector, Lutzomyia longipalpis. Eur J Immunol 2007; 37:3111–21. 17935072 10.1002/eji.200737431

[5] Oliveira F, Traore B, Gomes R, Faye O, Gilmore DC, Keita S, Delayed-type hypersensitivity to sand fly saliva in humans from a leishmaniasis-endemic area of Mali is Th1-mediated and persists to midlife. J Invest Dermatol 2013; 133:452–9. 22992802 10.1038/jid.2012.315 PMC3529997

[6] Carvalho AM, Viana SM, Andrade BB, Oliveira F, Valenzuela JG, Carvalho EM, de Oliveira CI. Immune Response to LinB13, a Lutzomyia Intermedia Salivary Protein Correlates With Disease Severity in Tegumentary Leishmaniasis. Clin Infect Dis 2022; 75:1754–62. 35385578 10.1093/cid/ciac258 PMC9662176

[7] Mondragon-Shem K, Al-Salem WS, Kelly-Hope L, Abdeladhim M, Al-Zahrani MH, Valenzuela JG, Acosta-Serrano A. Severity of old world cutaneous leishmaniasis is influenced by previous exposure to sandfly bites in Saudi Arabia. PLoS Negl Trop Dis 2015; 9:e0003449. 25646796 10.1371/journal.pntd.0003449 PMC4315490

[8] de Araujo FF, Abdeladhim M, Teixeira C, Hummer K, Wilkerson MD, Ressner R, Immune response profiles from humans experimentally exposed to Phlebotomus duboscqi bites. Front Immunol 2024; 15:1335307. 38633260 10.3389/fimmu.2024.1335307 PMC11021656

[9] Lerner EA, Ribeiro JM, Nelson RJ, Lerner MR. Isolation of maxadilan, a potent vasodilatory peptide from the salivary glands of the sand fly Lutzomyia longipalpis. J Biol Chem 1991; 266:11234–6. 2040631

[10] Kamhawi S, Belkaid Y, Modi G, Rowton E, Sacks D. Protection against cutaneous leishmaniasis resulting from bites of uninfected sand flies. Science 2000; 290:1351–4. 11082061 10.1126/science.290.5495.1351

[11] Oliveira F, Rowton E, Aslan H, Gomes R, Castrovinci PA, Alvarenga PH, A sand fly salivary protein vaccine shows efficacy against vector-transmitted cutaneous leishmaniasis in nonhuman primates. Sci Transl Med 2015; 7:290ra90. 10.1126/scitranslmed.aaa3043 26041707

[12] Winer H, Rodrigues GOL, Hixon JA, Aiello FB, Hsu TC, Wachter BT, IL-7: Comprehensive review. Cytokine 2022; 160:156049. 36201890 10.1016/j.cyto.2022.156049

[13] Oliveira F, Lawyer PG, Kamhawi S, Valenzuela JG. Immunity to distinct sand fly salivary proteins primes the anti-Leishmania immune response towards protection or exacerbation of disease. PLoS Negl Trop Dis 2008; 2:e226. 18414648 10.1371/journal.pntd.0000226 PMC2291569

[14] Antonelli LR, Dutra WO, Oliveira RR, Torres KC, Guimaraes LH, Bacellar O, Gollob KJ. Disparate immunoregulatory potentials for double-negative (CD4- CD8-) alpha beta and gamma delta T cells from human patients with cutaneous leishmaniasis. Infect Immun 2006; 74:6317–23. 16923794 10.1128/IAI.00890-06 PMC1695524

[15] Ferraz R, Cunha CF, Pimentel MIF, Lyra MR, Pereira-Da-Silva T, Schubach AO, CD3(+)CD4(neg)CD8(neg) (double negative) T lymphocytes and NKT cells as the main cytotoxic-related-CD107a(+) cells in lesions of cutaneous leishmaniasis caused by Leishmania (Viannia) braziliensis. Parasit Vectors 2017; 10:219. 28468680 10.1186/s13071-017-2152-2 PMC5415843

[16] Mou Z, Liu D, Okwor I, Jia P, Orihara K, Uzonna JE. MHC class II restricted innate-like double negative T cells contribute to optimal primary and secondary immunity to Leishmania major. PLoS Pathog 2014; 10:e1004396. 25233487 10.1371/journal.ppat.1004396 PMC4169504

[17] Theodor O. A study of the reaction to phlebotomus bites with some remarks on “Harara”. Trans R Soc Trop Med Hyg 1935; 29:273–84.

[18] Alexander JO. Papular urticaria and immune complexes. J Am Acad Dermatol 1985; 12:374–5. 3973134 10.1016/s0190-9622(85)80065-7

[19] Strobl J, Haniffa M. Functional heterogeneity of human skin-resident memory T cells in health and disease. Immunol Rev 2023; 316:104–19. 37144705 10.1111/imr.13213 PMC10952320

[20] Vogt MB, Lahon A, Arya RP, Kneubehl AR, Spencer Clinton JL, Paust S, Rico-Hesse R. Mosquito saliva alone has profound effects on the human immune system. PLoS Negl Trop Dis 2018; 12:e0006439. 29771921 10.1371/journal.pntd.0006439 PMC5957326

[21] Ariotti S, Hogenbirk MA, Dijkgraaf FE, Visser LL, Hoekstra ME, Song JY, T cell memory. Skin-resident memory CD8(+) T cells trigger a state of tissue-wide pathogen alert. Science 2014; 346:101–5. 25278612 10.1126/science.1254803

[22] Kadoki M, Patil A, Thaiss CC, Brooks DJ, Pandey S, Deep D, Organism-Level Analysis of Vaccination Reveals Networks of Protection across Tissues. Cell 2017; 171:398–413 e21. 28942919 10.1016/j.cell.2017.08.024 PMC7895295

[23] Asojo OA, Kelleher A, Liu Z, Pollet J, Hudspeth EM, Rezende WC, Structure of SALO, a leishmaniasis vaccine candidate from the sand fly Lutzomyia longipalpis. PLoS Negl Trop Dis 2017; 11:e0005374. 28278244 10.1371/journal.pntd.0005374 PMC5344329

[24] Ferreira VP, Fazito Vale V, Pangburn MK, Abdeladhim M, Mendes-Sousa AF, Coutinho-Abreu IV, SALO, a novel classical pathway complement inhibitor from saliva of the sand fly Lutzomyia longipalpis. Sci Rep 2016; 6:19300. 26758086 10.1038/srep19300 PMC4725370

[25] Cecilio P, Perez-Cabezas B, Fernandez L, Moreno J, Carrillo E, Requena JM, Preclinical antigenicity studies of an innovative multivalent vaccine for human visceral leishmaniasis. PLoS Negl Trop Dis 2017; 11:e0005951. 29176865 10.1371/journal.pntd.0005951 PMC5720812

[26] Fiuza JA, Dey R, Davenport D, Abdeladhim M, Meneses C, Oliveira F, Intradermal Immunization of Leishmania donovani Centrin Knock-Out Parasites in Combination with Salivary Protein LJM19 from Sand Fly Vector Induces a Durable Protective Immune Response in Hamsters. PLoS Negl Trop Dis 2016; 10:e0004322. 26752686 10.1371/journal.pntd.0004322 PMC4708988

[27] Gomes R, Teixeira C, Teixeira MJ, Oliveira F, Menezes MJ, Silva C, Immunity to a salivary protein of a sand fly vector protects against the fatal outcome of visceral leishmaniasis in a hamster model. Proc Natl Acad Sci U S A 2008; 105:7845–50. 18509051 10.1073/pnas.0712153105 PMC2397325

[28] Tavares NM, Silva RA, Costa DJ, Pitombo MA, Fukutani KF, Miranda JC, Lutzomyia longipalpis saliva or salivary protein LJM19 protects against Leishmania braziliensis and the saliva of its vector, Lutzomyia intermedia. PLoS Negl Trop Dis 2011; 5:e1169. 21655303 10.1371/journal.pntd.0001169 PMC3104964

[29] Collin N, Assumpcao TC, Mizurini DM, Gilmore DC, Dutra-Oliveira A, Kotsyfakis M, Lufaxin, a novel factor Xa inhibitor from the salivary gland of the sand fly Lutzomyia longipalpis blocks protease-activated receptor 2 activation and inhibits inflammation and thrombosis in vivo. Arterioscler Thromb Vasc Biol 2012; 32:2185–98. 22796577 10.1161/ATVBAHA.112.253906 PMC3421056

[30] Mendes-Sousa AF, do Vale VF, Silva NCS, Guimaraes-Costa AB, Pereira MH, Sant’Anna MRV, The Sand Fly Salivary Protein Lufaxin Inhibits the Early Steps of the Alternative Pathway of Complement by Direct Binding to the Proconvertase C3b-B. Front Immunol 2017; 8:1065. 28912782 10.3389/fimmu.2017.01065 PMC5583147

[31] Collin N, Gomes R, Teixeira C, Cheng L, Laughinghouse A, Ward JM, Sand fly salivary proteins induce strong cellular immunity in a natural reservoir of visceral leishmaniasis with adverse consequences for Leishmania. PLoS Pathog 2009; 5:e1000441. 19461875 10.1371/journal.ppat.1000441 PMC2677456

[32] Xu X, Oliveira F, Chang BW, Collin N, Gomes R, Teixeira C, Structure and function of a “yellow” protein from saliva of the sand fly Lutzomyia longipalpis that confers protective immunity against Leishmania major infection. J Biol Chem 2011; 286:32383–93. 21795673 10.1074/jbc.M111.268904 PMC3173228

[33] Cecilio P, Oristian J, Meneses C, Serafim TD, Valenzuela JG, Cordeiro da Silva A, Oliveira F. Engineering a vector-based pan-Leishmania vaccine for humans: proof of principle. Sci Rep 2020; 10:18653. 33122717 10.1038/s41598-020-75410-0 PMC7596519

[34] Zundler S, Neurath MF. Interleukin-12: Functional activities and implications for disease. Cytokine Growth Factor Rev 2015; 26:559–68. 26182974 10.1016/j.cytogfr.2015.07.003

[35] Schmitt E, Rude E, Germann T. The immunostimulatory function of IL-12 in T-helper cell development and its regulation by TGF-beta, IFN-gamma and IL-4. Chem Immunol 1997; 68:70–85. 9329217 10.1159/000058695

[36] Castilho TM, Goldsmith-Pestana K, Lozano C, Valderrama L, Saravia NG, McMahon-Pratt D. Murine model of chronic L. (Viannia) panamensis infection: role of IL-13 in disease. Eur J Immunol 2010; 40:2816–29. 20827674 10.1002/eji.201040384 PMC3289133

[37] Matthews DJ, Emson CL, McKenzie GJ, Jolin HE, Blackwell JM, McKenzie AN. IL-13 is a susceptibility factor for Leishmania major infection. J Immunol 2000; 164:1458–62. 10640762 10.4049/jimmunol.164.3.1458

[38] Zaatar MT, Simaan Y, Karam MC. Exogenous IL-13 exacerbates Leishmania major infection and abrogates acquired immunity to re-infection. Parasitol Res 2022; 121:2009–17. 35536514 10.1007/s00436-022-07539-y

[39] Alexander J, Brombacher F, McGachy HA, McKenzie AN, Walker W, Carter KC. An essential role for IL-13 in maintaining a non-healing response following Leishmania mexicana infection. Eur J Immunol 2002; 32:2923–33. 12355446 10.1002/1521-4141(2002010)32:10<2923::AID-IMMU2923>3.0.CO;2-E

[40] Bourreau E, Prevot G, Pradinaud R, Launois P. Interleukin (IL)-13 is the predominant Th2 cytokine in localized cutaneous leishmaniasis lesions and renders specific CD4+ T cells unresponsive to IL-12. J Infect Dis 2001; 183:953–9. 11237813 10.1086/319249

[41] Junior J, de Souza JL, da Silva LS, da Silva CC, do Nascimento TA, de Souza MLG, A fine mapping of single nucleotide variants and haplotype analysis of IL13 gene in patients with Leishmania guyanensis-cutaneous leishmaniasis and plasma cytokines IL-4, IL-5, and IL-13. Front Immunol 2023; 14:1232488. 10.3389/fimmu.2023.1232488 PMC10613733 37908348

[42] Souissi C, Marzouki S, Elbini-Dhouib I, Jebali J, Oliveira F, Valenzuela JG, PpSP32, the Phlebotomus papatasi immunodominant salivary protein, exerts immunomodulatory effects on human monocytes, macrophages, and lymphocytes. Parasit Vectors 2023; 16:1. 36593519 10.1186/s13071-022-05627-7 PMC9806891

[43] Tanaka T, Narazaki M, Kishimoto T. IL-6 in inflammation, immunity, and disease. Cold Spring Harb Perspect Biol 2014; 6:a016295. 25190079 10.1101/cshperspect.a016295 PMC4176007

[44] Ehrchen JM, Roebrock K, Foell D, Nippe N, von Stebut E, Weiss JM, Keratinocytes determine Th1 immunity during early experimental leishmaniasis. PLoS Pathog 2010; 6:e1000871. 20442861 10.1371/journal.ppat.1000871 PMC2861693

[45] Stager S, Maroof A, Zubairi S, Sanos SL, Kopf M, Kaye PM. Distinct roles for IL-6 and IL-12p40 in mediating protection against Leishmania donovani and the expansion of IL-10+ CD4+ T cells. Eur J Immunol 2006; 36:1764–71. 16791879 10.1002/eji.200635937 PMC2659577

[46] Adachi T, Kobayashi T, Sugihara E, Yamada T, Ikuta K, Pittaluga S, Hair follicle-derived IL-7 and IL-15 mediate skin-resident memory T cell homeostasis and lymphoma. Nat Med 2015; 21:1272–9. 26479922 10.1038/nm.3962 PMC4636445

[47] Scott P. Long-Lived Skin-Resident Memory T Cells Contribute to Concomitant Immunity in Cutaneous Leishmaniasis. Cold Spring Harb Perspect Biol 2020; 12. 10.1101/cshperspect.a038059 PMC7528853 32839202

[48] Lima-Junior DS, Costa DL, Carregaro V, Cunha LD, Silva AL, Mineo TW, Inflammasome-derived IL-1beta production induces nitric oxide-mediated resistance to Leishmania. Nat Med 2013; 19:909–15. 23749230 10.1038/nm.3221

[49] Charmoy M, Hurrell BP, Romano A, Lee SH, Ribeiro-Gomes F, Riteau N, The Nlrp3 inflammasome, IL-1beta, and neutrophil recruitment are required for susceptibility to a nonhealing strain of Leishmania major in C57BL/6 mice. Eur J Immunol 2016; 46:897–911. 26689285 10.1002/eji.201546015 PMC4828310

[50] Dey R, Joshi AB, Oliveira F, Pereira L, Guimaraes-Costa AB, Serafim TD, Gut Microbes Egested during Bites of Infected Sand Flies Augment Severity of Leishmaniasis via Inflammasome-Derived IL-1beta. Cell Host Microbe 2018; 23:134–43 e6. 29290574 10.1016/j.chom.2017.12.002 PMC5832060

[51] Fernandez-Figueroa EA, Rangel-Escareno C, Espinosa-Mateos V, Carrillo-Sanchez K, Salaiza-Suazo N, Carrada-Figueroa G, Disease severity in patients infected with Leishmania mexicana relates to IL-1beta. PLoS Negl Trop Dis 2012; 6:e1533. 22629474 10.1371/journal.pntd.0001533 PMC3358333

[52] Novais FO, Carvalho AM, Clark ML, Carvalho LP, Beiting DP, Brodsky IE, CD8+ T cell cytotoxicity mediates pathology in the skin by inflammasome activation and IL-1beta production. PLoS Pathog 2017; 13:e1006196. 28192528 10.1371/journal.ppat.1006196 PMC5325592

[53] Santos D, Campos TM, Saldanha M, Oliveira SC, Nascimento M, Zamboni DS, IL-1beta Production by Intermediate Monocytes Is Associated with Immunopathology in Cutaneous Leishmaniasis. J Invest Dermatol 2018; 138:1107–15. 29246797 10.1016/j.jid.2017.11.029 PMC5912958

[54] Kumar R, Bunn PT, Singh SS, Ng SS, Montes de Oca M, De Labastida Rivera F, Type I Interferons Suppress Anti-parasitic Immunity and Can Be Targeted to Improve Treatment of Visceral Leishmaniasis. Cell Rep 2020; 30:2512–25 e9. 32101732 10.1016/j.celrep.2020.01.099 PMC7981274

[55] Xin L, Vargas-Inchaustegui DA, Raimer SS, Kelly BC, Hu J, Zhu L, Type I IFN receptor regulates neutrophil functions and innate immunity to Leishmania parasites. J Immunol 2010; 184:7047–56. 20483775 10.4049/jimmunol.0903273 PMC4159077

[56] Mattner J, Schindler H, Diefenbach A, Rollinghoff M, Gresser I, Bogdan C. Regulation of type 2 nitric oxide synthase by type 1 interferons in macrophages infected with Leishmania major. Eur J Immunol 2000; 30:2257–67. 10940917 10.1002/1521-4141(2000)30:8<2257::AID-IMMU2257>3.0.CO;2-U

[57] Haddow AD, Curler G, Moulton JK. New records of Lutzomyia shannoni and Lutzomyia vexator (Diptera: Psychodidae) in eastern Tennessee. J Vector Ecol 2008; 33:393–6. 19263861 10.3376/1081-1710-33.2.393

