# Human sand fly challenge elicits saliva-specific innate and T_H_1-polarized immunity that promotes *Leishmania* killing

**DOI:** 10.1101/2025.02.25.640210

**Published:** 2025-09-30

**Authors:** Maha Abdeladhim, Clarissa Teixeira, Roseanne Ressner, Kelly Hummer, Ranadhir Dey, Regis Gomes, Waldionê de Castro, Fernanda Fortes de Araujo, George W. Turiansky, Eva Iniguez, Claudio Meneses, Fabiano Oliveira, Naomi Aronson, Joshua R. Lacsina, Jesus G. Valenzuela, Shaden Kamhawi

**Affiliations:** 1 Vector Molecular Biology Section, Laboratory of Malaria and Vector Research, National Institute of Allergy and Infectious Diseases, National Institutes of Health, Rockville, Maryland, United States.; 2 Laboratory of Immunoparasitology, Department of Biotechnology, Oswaldo Cruz Foundation, Eusébio, Ceará, Brazil.; 3 Walter Reed National Military Medical Center, Bethesda, Maryland, United States.; 4 Center for Infectious Disease Research, Walter Reed Army Institute of Research, Silver Spring, Maryland, United States.; 5 Infectious Diseases Division, Department of Medicine, Uniformed Services University of the Health Sciences, Bethesda, Maryland, United States.; 6 Henry M Jackson Foundation for the Advancement of Military Medicine, Bethesda, Maryland, United States.; 7 Division of Emerging and Transfusion Transmitted Diseases, Office of Blood Research and Review, Center for Biologics Evaluation and Research, Food and Drug Administration, Silver Spring, Maryland, United States.; 8 Department of Dermatology, Uniformed Services University of the Health Sciences, Bethesda, Maryland, United States.

## Abstract

In *Leishmania*-endemic areas, humans are constantly exposed to sand fly bites. To explore the immune consequences of this chronic vector exposure, we performed a human challenge study with the sand fly *Lutzomyia longipalpis*. Peripheral blood mononuclear cells were collected from fifteen healthy volunteers who underwent multiple controlled exposures to sand fly bites. We identified two *Lu. longipalpis* salivary proteins, LJM19 and LJL143, which elicited T_H_1-polarized cytokine responses in cells from exposed individuals and which correlated with enhanced killing of *Leishmania* parasites in co-cultured macrophages. Interestingly, LJM19 also exerted this parasite-killing effect in cells from unexposed individuals, consistent with innate immune activation. In support of this, both LJM19 and LJL143 stimulated the production of the innate cytokines IL-1β and IFN-α. Our results demonstrate that repeated exposure to sand fly bites induces innate and adaptive cytokine responses to vector salivary proteins that can be co-opted to protect humans against *Leishmania* infection.

## INTRODUCTION

Leishmaniasis is a neglected vector borne disease caused by protozoan parasites of the genus *Leishmania*, which are transmitted via the bite of phlebotomine sand flies. Each year, over 700,000 new cases of leishmaniasis are reported worldwide.^1^ Sand fly saliva plays a critical role in *Leishmania* transmission. When female sand flies bite their host to take a blood meal, they inject a complex mixture of immunogenic and biologically active salivary proteins into the skin. Collectively, these foreign salivary antigens trigger a host immune response that favors parasite establishment.^2^

In *Leishmania*-endemic areas, humans are constantly exposed to these salivary antigens, resulting in an adaptive immune response which induces durable cytokine responses from peripheral blood mononuclear cells (PBMCs). These cytokine responses can be recalled up to ten years after the most recent sand fly exposure.^3,4^ Similarly in the skin, the ability of sand fly bites to elicit a delayed-type hypersensitivity (DTH) response can persist for decades in humans with ongoing sand fly exposure.^5^ For patients with cutaneous leishmaniasis (CL), their cellular and antibody responses to certain sand fly salivary proteins strongly correlate with CL lesion size and whether the disease is localized or disseminated.^6,7^ This suggests that human immunity to sand fly saliva modulates disease severity and susceptibility to leishmaniasis. Furthermore, while there have been numerous clinical studies on how factors like chronic malnutrition^8^ and helminth infection^9^ affect the immune system, there is only a handful of studies that have investigated how repeated vector bites and exposure to salivary antigens – many of which are immunomodulatory – impact immune responses in humans.^4,5,10,11^

Human immunity to sand fly saliva has been investigated through human vector challenge studies, where healthy volunteers are exposed to the bites of uninfected sand flies. Although only a few such human challenge studies have been conducted, they demonstrate that most volunteers develop a skin DTH response to sand fly bites and a mixed cellular T_H_1/T_H_2 cytokine response when their PBMCs are stimulated with salivary gland extract (SGE) *ex vivo*.^4,5,10^ One of the recent human challenge studies extended these findings by identifying individual salivary proteins from the Afro-Asian sand fly *Phlebotomus duboscqi* that induce strong IFN-γ or IL-10 responses in PBMCs.^10^

In this human sand fly challenge study, we investigated cellular immunity to saliva of the sand fly *Lutzomyia longipalpis* (*Lu. longipalpis*), the principal vector of visceral leishmaniasis in the Americas, in individuals exposed to uninfected *Lu. longipalpis* bites multiple times over several months. Our objective was to simulate the repeated exposure to sand fly saliva that occurs in *Leishmania*-endemic populations. We identified two sand fly salivary proteins that elicit robust innate and T_H_1-polarized cytokine responses in human immune cells and enhance killing of *Leishmania* parasites *ex vivo*.

## RESULTS

### Characteristics of study participants

**Fig 1.**
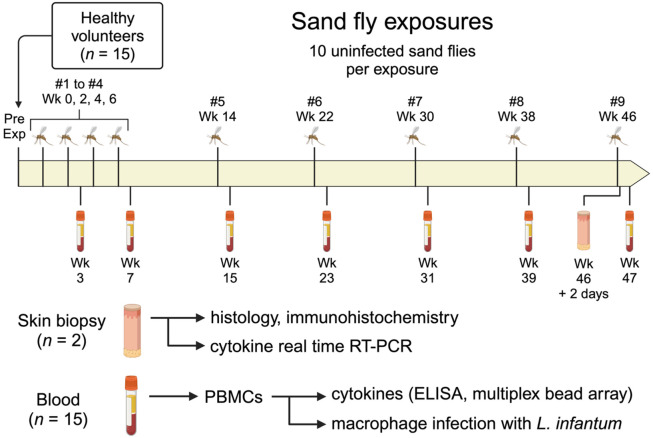
Schematic of clinical study. Healthy volunteers (*n* = 15) were exposed to the bites of uninfected *Lutzomyia longipalpis* sand flies once every two weeks for exposures #1 through #4, then once every eight weeks for exposures #5 to #9. Blood was collected ~1 week after exposures #2, #4, and #5 to #9, followed by PBMC isolation to perform the indicated assays. Skin punch biopsies were obtained from two participants 48 hours after exposure #9, with one biopsy from a sand fly bite site and one biopsy from the contralateral arm for each participant. *Pre Exp*, pre-exposure study visit. Created in BioRender.com.

Table 1 presents the demographics, allergy history, and baseline plasma IgE levels of the study participants. Fifteen healthy volunteers were enrolled in the study. The median age was 27 years (range 22 to 46), and the majority were male (12 of 15, 80%). Figure 1 provides a schematic of the study design. Loss to follow-up began at exposure #6, primarily due to active duty military participants being transferred out of the study area (*n* = 4) and one participant being discharged from the military. Three participants halted their participation due to study-related reactions to sand fly bites, which included large areas of urticaria, pruritus, and erythema, raising concerns about potentially worsening symptoms with future sand fly exposures. The number of fed sand flies was high and consistent across all feedings, with an average of 8 to 9 fully fed and 0 to 1 partially fed sand flies out of 10 total per participant at each exposure.

### Progressive exposures to *Lu. longipalpis* alter the bite site rash phenotype

**Fig 2.**
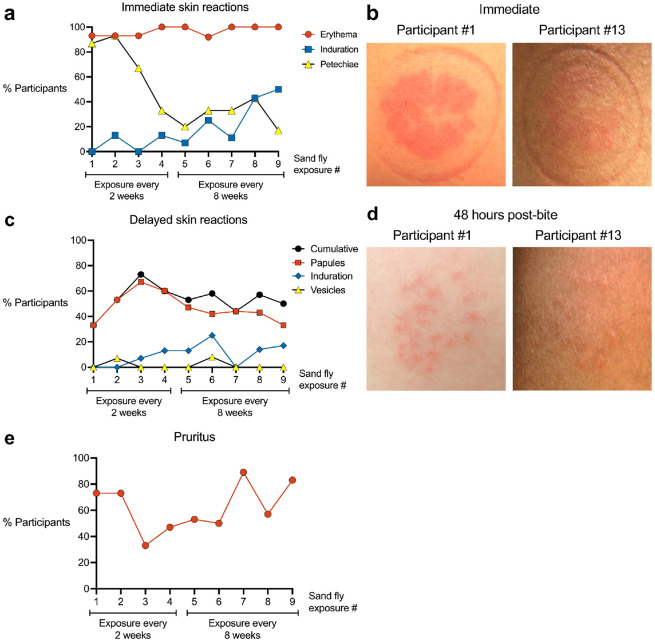
Characteristics of rashes at the sites of uninfected *Lutzomyia longipalpis* bites. **a** Skin reactions observed within the first 10 minutes after completion of each sand fly exposure. **b** Representative photographs from two participants immediately after Exposure #9. **c** Delayed skin reactions at the bite site that developed one or more days after completion of each sand fly exposure. **d** Representative photographs from two participants 48 hours after Exposure #9. **e** Pruritus at the bite site.

We characterized the phenotype and symptoms of rashes induced by uninfected *Lu. longipalpis* bites across multiple sand fly exposures. For immediate bite site reactions, 85% to 100% of participants exhibited erythema across all exposures (Fig. 2a, b), consistent with the potent vasodilatory activity of the *Lu. longipalpis* salivary protein maxadilan.^12^ Petechiae decreased with successive exposures, with only one of six participants exhibiting petechiae at the final exposure. Immediate induration was initially absent but increased to 50% of participants by the final exposure.

We next evaluated delayed bite site reactions that developed hours to one week after each sand fly exposure (Fig. 2c, d). Papules were the most frequent delayed rash phenotype (Fig. 2c), increasing from 33% to 67% of participants over the first three exposures before declining to 33% by the final exposure. Induration and vesicles appeared inconsistently in a maximum of 23% and 8% of participants, respectively. Localized pruritus at the bite site (either delayed or immediate) was initially reported by 73% of participants and increased to 83% at the final exposure (Fig. 2e). Across all exposures, the median pruritus duration was 2 days (range 30 minutes to 3 months).

Interestingly, for 8 of 15 participants, sand fly exposure triggered the appearance of a rash at a previous bite site on the contralateral arm, which had been exposed weeks before (median 6.5 weeks prior, range 2 to 11 weeks). This phenomenon, termed distal bite site reactivation, manifested approximately one week after the most recent sand fly exposure (median 4.5 exposures, range 2 to 9).

### *Lu. longipalpis* bites induce a delayed-type hypersensitivity skin response after multiple sand fly exposures

Forty-eight hours after the final sand fly exposure, two participants (#1 and #13) who completed all nine exposures volunteered for skin biopsies from a bite site and from the contralateral arm as a negative control. Both participants exhibited multiple erythematous papules at their exposure site, characteristic of a DTH response (Fig. 2d).

**Fig 3.**
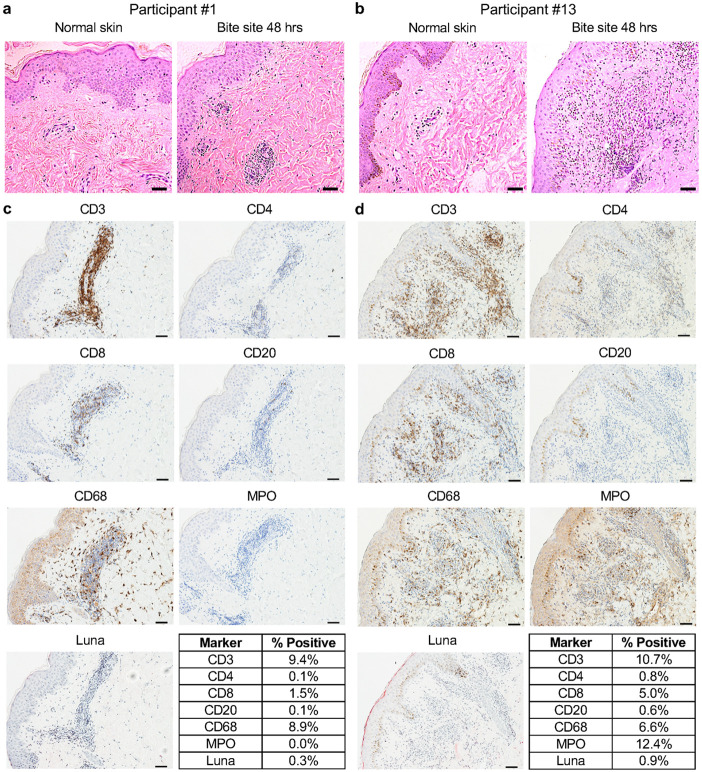
Repeated exposures to uninfected Lu. longipalpis bites induce a delayed-type hypersensitivity response at the bite site. Skin punch biopsies were collected from bite site skin and from normal appearing skin on the contralateral arm from two participants 48 hours after exposure #9. **a, b** Hematoxylin and eosin stains. **c, d** Immunohistochemistry (IHC) of the indicated markers at the bite site. Tables show the percentage of cells that stained positive for each marker based on analysis with ImageJ. Scale bar is 50 μm for all images.

Histology from Participant #1 showed spongiosis and a perivascular mononuclear (lymphocytic and histiocytic) infiltrate with occasional eosinophils (Fig. 3a). Participant #13 exhibited a diffuse, mixed inflammatory infiltrate with both neutrophils and mononuclear cells present in the upper dermis, distributed throughout the perivascular, interstitial, and perieccrine areas (Fig. 3b).

Immunohistochemistry (IHC) revealed that the infiltrate in both participants was largely composed of T cells (CD3) and macrophages (CD68), while B cells (CD20) and eosinophils (Luna) were rare or absent, respectively (Fig. 3c, d). Interestingly, the CD4 signal was minimal (Participant #13) to absent (Participant #1), while the CD8 signal overlapped with only a fraction of the CD3 staining pattern. Thus, the CD4 and CD8 signals do not fully account for the strong CD3 signal observed in both individuals. While Participant #1 had no detectable neutrophils by myeloperoxidase (MPO) stain (Fig. 3c), Participant #13 showed MPO staining throughout the dermis (Fig. 3d). These data highlight macrophages and T cells as the predominant immune cells driving the DTH response to *Lu. longipalpis* bites in these two individuals. The IHC data also suggest that CD4^−^CD8^−^ T cells (termed double negative or DN T cells) may play a role in the DTH response to sand fly bites.

Quantitative RT-PCR of the skin biopsies elucidated the cutaneous T_H_ cytokine profile at the bite site (Fig. S1). Participant #1 exhibited a mixed T_H_1/T_H_2 response with similar levels of IFN-γ and IL-13, and only minor contributions from IL-12 and IL-5. In contrast, Participant #13 showed a dominant T_H_1 response driven by high expression of IFN-γ, no detectable IL-13, and comparatively low expression of IL-12 and IL-5.

### *Lu. longipalpis* bites induce cellular interferon gamma responses to the sand fly salivary proteins LJL143 and LJM19

**Fig 4.**
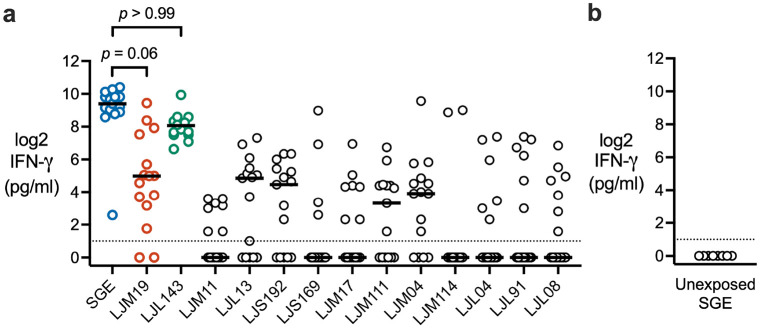
Recombinant salivary proteins stimulate IFN-γ production in PBMCs from *Lu. longipalpis*-exposed volunteers. **a** PBMCs were stimulated with salivary gland extract (SGE) or the indicated recombinant *Lu. longipalpis* salivary protein to measure IFN-γ levels by ELISA. PBMCs were from exposure #2 or #4 with one time point per volunteer (*n* = 15). *P*-values are shown only for comparisons with *p* > 0.05 that showed no significant difference from SGE. **b** As a negative control, IFN-γ was measured in SGE-stimulated PBMCs from healthy volunteers unexposed to *Lu. longipalpis* (*n* = 8). Dashed lines indicate limit of detection. *P*-values were calculated by Friedman test with Dunn’s multiple comparisons test.

In preclinical models, distinct sand fly salivary proteins were identified as immunogenic, inducing a strong IFN-γ response which correlated with protection against leishmaniasis.^13-17^ After the second or fourth sand fly exposure, we collected peripheral blood mononuclear cells (PBMCs) from our study participants to screen the thirteen most abundant proteins in *Lu. longipalpis* saliva for their ability to stimulate IFN-γ production (Fig. 4a, Table S1). All recombinant proteins were verified to be endotoxin-free (<20 EU/ml). LJL143 and LJM19 were the only salivary proteins that stimulated IFN-γ production at a level comparable to salivary gland extract (SGE), a standard for immunogenicity. Stimulation with any of the other eleven *Lu. longipalpis* salivary proteins resulted in significantly less IFN-γ production (all *p* < 0.01). As a negative control, no IFN-γ production was elicited by SGE treatment of PBMCs from *Lu. longipalpis*-unexposed individuals obtained from the NIH Blood Bank (Fig. 4b). These results suggest that repeated exposure of humans to *Lu. longipalpis* bites leads to the development of a robust cellular IFN-γ response to the sand fly salivary proteins LJM19 and LJL143.

### LJM19 and LJL143 induce a T_H_1-polarized cytokine response

**Fig 5.**
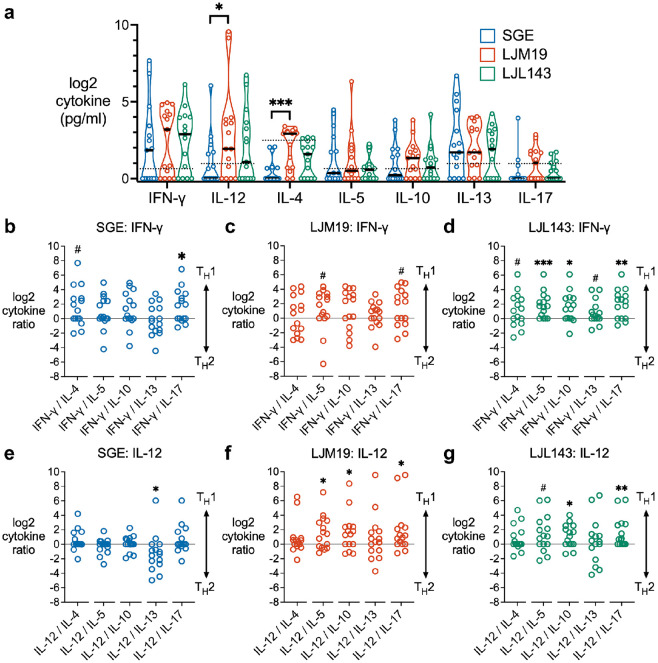
LJM19 and LJL143 induce T_H_1-polarized cytokine responses in PBMCs from *Lu. longipalpis*-exposed volunteers. **a** PBMCs obtained from sand fly-exposed study participants (*n* = 15) were stimulated with SGE, LJM19, or LJL143. Cell supernatants were collected at 96 hours and cytokine concentrations were measured by multiplex bead array. Black bar indicates the median. Dashed lines indicate limit of detection. For each cytokine, differences between treatment groups were analyzed by Friedman test with Dunn’s test for multiple comparisons. PBMCs were from exposure #4 to #9 with one time point per participant. Ratios of T_H_1 to T_H_2 or T_H_17 cytokines were calculated for PBMCs treated with SGE (**b, e**), LJM19 (**c, f**), or LJL143 (**d, g**). Ratios above 0 (solid line) indicate a T_H_1-polarized response while ratios below 0 indicate a T_H_2- or T_H_17-polarized response, as analyzed by Wilcoxon signed-rank test using a value of zero as the null hypothesis (equal balance of T_H_1 and T_H_2/T_H_17 cytokines), # *p* < 0.10, * *p* < 0.05, ** *p* < 0.01, *** *p* < 0.001.

We next sought to elucidate the broader spectrum of T_H_ cytokines induced by LJM19 and LJL143 compared to whole saliva (SGE). To assess these cytokine responses, we used PBMCs collected after later exposures four through nine (Table S1). PBMCs were stimulated with SGE, LJM19, or LJL143, and cytokines were measured in the supernatants by multiplex bead array. Comparing the three stimulation conditions for each T_H_ cytokine, we found that LJM19 induced significantly higher levels of IL-12 and IL-4 compared to SGE (Fig. 5a). Notably the T_H_2 cytokine IL-13 was consistently highly expressed across all three treatments.

To quantify T_H_ polarization, we calculated the ratios of the T_H_1 cytokines, IFN-γ and IL-12, against each T_H_2 cytokine (IL-4, IL-5, IL-10, and IL-13) and the T_H_17 cytokine, IL-17. We calculated cytokine ratios with either IFN-γ or IL-12 to assess both T_H_1 effector function and upstream regulation of T_H_1 lineage commitment, respectively.^18^ SGE induced a mixed T_H_1/T_H_2 response with a trend towards T_H_1 seen for the IFN-γ/IL-4 ratio (*p* = 0.05) (Fig. 5b). LJM19 treatment also induced a comparatively weak IFN-γ response, with a trend towards T_H_1 seen only for IFN-γ/IL-5 (Fig. 5c). LJL143 induced significant T_H_1 polarization via IFN-γ relative to IL-5 and IL-10, with a trend towards T_H_1 for IFN-γ versus IL-4 or IL-13 (Fig. 5d). For IL-12, SGE induced significant T_H_2 polarization relative to IL-13 (Fig. 5e). In contrast, LJM19-treated PBMCs exhibited significant T_H_1 polarization with IL-12 relative to the T_H_2 cytokines IL-5 and IL-10 (Fig. 5f). For LJL143, IL-12 also showed significant T_H_1 polarization versus IL-10 and a trend versus IL-5 (Fig. 5g). None of the treatments induced a T_H_17-polarized response. For each individual, the ratios were largely consistent across all T_H_1/T_H_2 cytokine pairs, except for IL-13, which was consistently expressed at a higher level than the other T_H_2 cytokines (Fig. S2). In summary, LJL143 and LJM19 induce a T_H_1-polarized response in PBMCs from individuals with multiple *Lu. longipalpis* exposures. Notably, this T_H_1 response is observed primarily through IFN-γ for LJL143 and through IL-12 for LJM19 and is accompanied by high IL-13 expression.

### LJM19 and LJL143 induce innate inflammatory and antiviral cytokines

**Fig 6.**
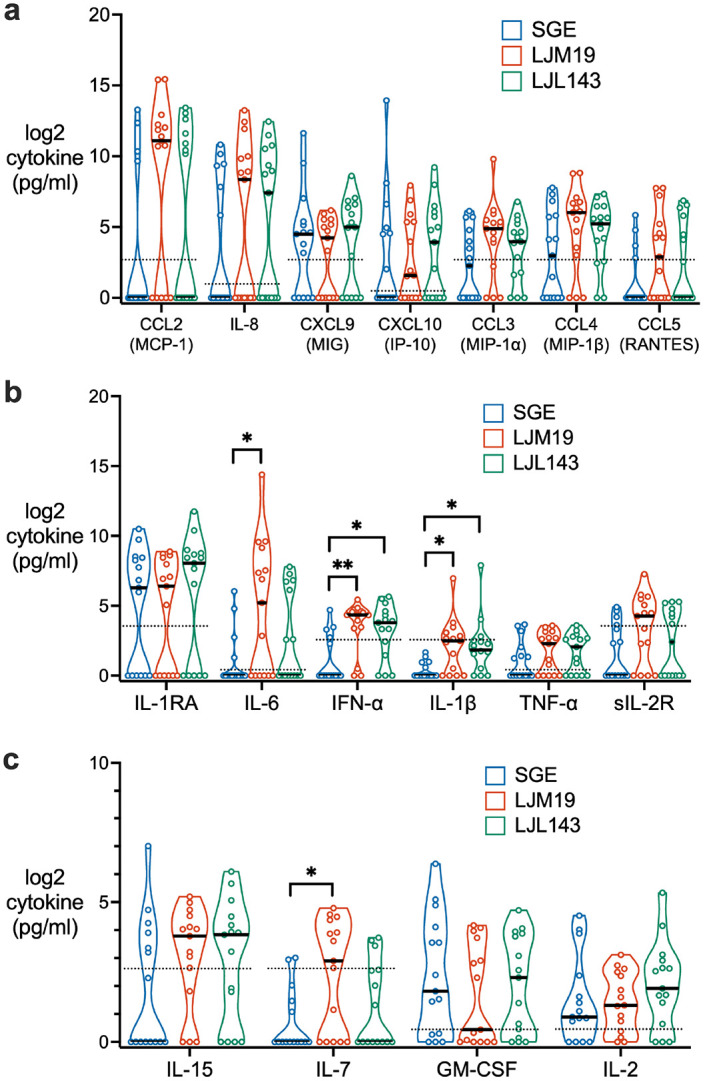
Differences in PBMC cytokine profiles induced by LJM19 or LJL143 compared to SGE. PBMCs obtained from Lu. longipalpis-exposed study participants (n = 15) were stimulated with SGE, LJM19, or LJL143. Cell supernatants were collected at 96 hours and cytokine concentrations were measured by multiplex bead array for chemokines (**a**), inflammatory cytokines (**b**), and cytokines promoting cell survival, activation, and proliferation (**c**). PBMCs used were from exposure #4 to #9 with one time point per participant. Black bar indicates the median. Dashed lines indicate limit of detection. For each cytokine, differences between treatment groups were analyzed by Friedman test with Dunn’s test for multiple comparisons, * p < 0.05, ** p < 0.01.

We investigated the effects of SGE, LJM19, and LJL143 on other functional classes of cytokines. No differences in chemokine concentration were observed between the three treatment groups (Fig. 6a). Compared to SGE, both LJM19 and LJL143 induced significantly higher levels of interferon alpha (IFN-α), an antiviral and immunomodulatory cytokine, and IL-1β, an inflammatory cytokine (Fig. 6b). Additionally, LJM19 induced higher levels of IL-6 (Fig. 6b), an inflammatory cytokine, and IL-7 (Fig. 6c), which promotes lymphocyte proliferation and maintenance in peripheral tissues.^19^

### LJM19 and LJL143 enhance the ability of PBMCs to stimulate macrophage killing of intracellular *Leishmania* parasites

**Fig 7.**
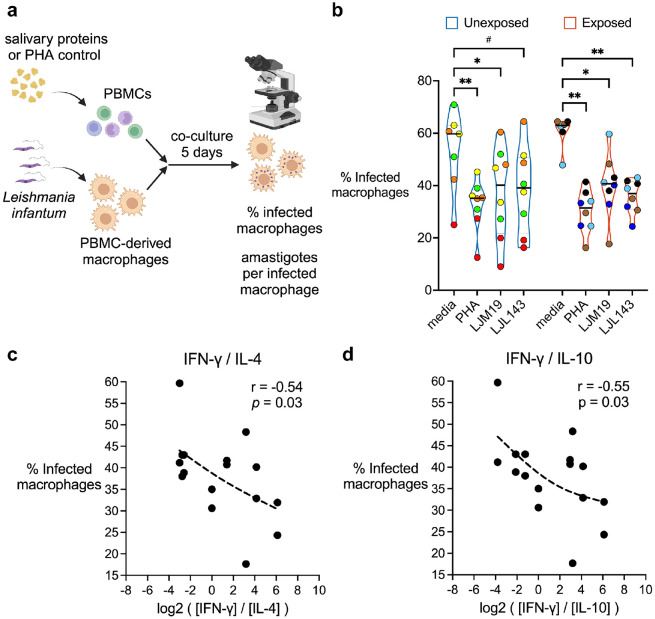
Stimulation of PBMCs with LJM19 or LJL143 enhances killing of *Leishmania* by macrophages. PBMC-derived macrophages from participants unexposed (*n* = 4, blue border) or exposed (*n* = 4, red border) to *Lu. longipalpis* were infected with *Leishmania infantum*, then co-cultured with autologous PBMCs that had been stimulated according to the conditions shown. After 5 days of co-culture, the percentage of infected macrophages was quantified by manual counting of Giemsa-stained cells by light microscopy. PBMCs were from exposure #7 to #9 with one time point per participant. **a** Experimental scheme. **b** Percentage of infected macrophages. Each volunteer’s batch of PBMCs was divided and run in two technical replicates; identical colors denote replicates from the same individual. Bar indicates the median. Differences between treatment groups were analyzed via a mixed effects model with Dunnett’s test for multiple comparisons, # *p* < 0.10, * *p* < 0.05, and ** *p* < 0.01. **c, d** Spearman correlation between the IFN-γ/IL-4 ratio (**c**) or IFN-γ/IL-10 ratio (**d**) as calculated in Figure 5 and the percentage of infected macrophages for LJM19- and LJL143-treated samples. Dashed line in **c, d** is the trend line (smoothing spline). Schematic in **a** created in BioRender.com.

Considering the T_H_1-polarized profiles of LJM19 and LJL143, we tested the hypothesis that treatment with these proteins enhances the ability of PBMCs to stimulate the killing of intracellular parasites by *Leishmania*-infected macrophages. We compared PBMCs from *Lu. longipalpis*-naïve individuals from the NIH Blood Bank to PBMCs from sand fly-exposed volunteers. For the latter group, we used PBMCs collected after exposures seven through nine from a subset of four participants to assess protection against *Leishmania* in heavily sand fly-exposed individuals (Table S1). Monocyte-derived macrophages were infected with *Leishmania infantum* and then co-cultured with autologous PBMCs stimulated with the sand fly salivary proteins LJM19 or LJL143. Phytohemagglutinin (PHA), a T cell mitogen, served as a positive control (Fig. 7a).

For *Lu. longipalpis*-exposed participants, PBMC stimulation with either LJM19 or LJL143 led to a significant reduction in the percentage of infected macrophages (Fig. 7b). This reduction was comparable in magnitude to that observed with PHA stimulation. Surprisingly, in PBMCs from *Lu. longipalpis*-unexposed individuals, stimulation with LJM19 alone also induced a significant decrease in the percentage of infected macrophages, while stimulation with LJL143 showed a trend towards a decrease. Furthermore, the difference in the percentage of infected macrophages between unexposed and exposed individuals when PBMCs were stimulated with either LJM19 (*p* = 0.70) or LJL143 (*p* = 0.68) was not significant. Nevertheless, exposed individuals showed a narrower data spread, particularly for LJL143 (standard deviation decreased 1.45-fold for LJM19 and 2.5-fold for LJL143). There were no significant differences between media alone and any of the treatment groups in the number of amastigotes per infected macrophage (Fig. S3). Cross-referencing these macrophage infection data with our earlier T_H_ cytokine ratio results revealed a significant negative correlation between the percentage of infected macrophages and the ratios of IFN-γ/IL-4 and IFN-γ/IL-10 (Fig. 7c, d), while no correlation was seen with the IFN-γ/IL-5 or IFN-γ/IL-13 ratios (Fig. S4). Similarly, no significant correlation was seen with IFN-γ alone (Fig. S4), indicating that IFN-γ is not the sole determinant of the parasite killing effect mediated by LJM19 and LJL143, but rather depends on the context of the T_H_ cytokine milieu.

## DISCUSSION

This study provides the most comprehensive characterization to date of how human skin and systemic immune responses evolve with longitudinal exposure to the bites of *Lutzomyia longipalpis* sand flies. Our cohort was larger than the only prior study to perform controlled human challenge with *Lutzomyia*,^4^ and had a participant retention rate comparable to a recent human challenge study with *Phlebotomus duboscqi*.^10^ All participants completed at least five exposures, which is sufficient to develop adaptive immunity against sand fly saliva and surpassing the prior benchmark of four exposures from the previous *Lutzomyia* challenge study.^4^ Moreover, several participants in our cohort completed up to nine exposures over the course of a year.

Stable DTH reactions to *Lu. longipalpis* bites developed and were maintained throughout the study, similar to those seen previously against *Phlebotomus duboscqi* bites.^5,10^ In preclinical models, saliva-specific T_H_1-polarized DTH responses are closely correlated with immune protection against *Leishmania*.^15,16^ In our study, skin biopsies of the bite site from two participants who completed all nine sand fly exposures exhibited variable DTH reactions that were either T_H_1-polarized or exhibited a mixed T_H_1/T_H_2 response. For both volunteers, the site of the DTH response was composed of T cells, macrophages, and in one participant, neutrophils. Both biopsy sites were dominated by CD3+ CD4− CD8− T cells, also known as double negative (DN) T cells. This corroborates prior findings in a cohort from Mali where three of six individuals with DTH to sand fly bites showed infiltration with DN T cells at the bite site, while CD4+ and CD8+ T cells were absent.^5^ While the potential role of DN T cells in the response to sand fly bites remains unclear, expanded DN T cell populations have been demonstrated in human CL lesions due to *L. braziliensis* and exhibit a highly activated phenotype.^20,21^ These DN T cells express an αβ T cell receptor (TCR) and show high expression of IFN-γ and CD69, a tissue residence marker.^20^ In mice, DN T cells are necessary for primary and secondary immunity to *L. major*.^22^ Further studies are needed to investigate the dominant T_H_ polarization profile at the sand fly bite site, whether DN T cells are a consistent and prominent feature of the DTH response, and how sand fly saliva-primed T_H_ and DN T cells influence immunity to *Leishmania* parasites.

In over half of participants, *Lu. longipalpis* bites triggered the reappearance of a rash at a previously bitten site on the contralateral arm. This phenomenon, known as distal bite site reactivation, was also reported after uninfected *Phlebotomus duboscqi* bites^10^ and during early studies of human skin reactions to the bites of *Phlebotomus papatasi*.^23^ We speculate that this reactivation reflects the activation of salivary protein-specific, skin resident memory T cells that have seeded a prior bite site and respond to circulating salivary antigens from new sand fly bites.^24,25^ The ability of vector bites to impact systemic immunity was demonstrated in humanized mice exposed to uninfected *Aedes aegypti* bites.^26^ These mice exhibited significant changes in blood cytokine levels and immune cell composition in both blood and skin up to seven days after mosquito exposure. This suggests the intriguing possibility that local cutaneous responses to vector bites may trigger systemic signals that promote a pathogen-resistant state throughout the skin, similar to the organism-wide coordination of antiviral immunity.^27,28^

Using PBMCs, our screen identified LJM19 and LJL143 as the two *Lu. longipalpis* salivary proteins that stimulate the highest IFN-γ production in PBMCs from sand fly exposed participants, confirming their immunogenicity in humans. LJM19 (also named SALO) is an 11 kDa protein that inhibits the classical complement pathway and has no structural similarity to human proteins.^29,30^ LJM19 was previously identified as a salivary protein vaccine candidate that protects against both cutaneous and visceral leishmaniasis in preclinical models.^14,17,31,32^ LJL143 (also named Lufaxin) is an inhibitor of coagulation factor Xa and the alternative pathway of complement.^33,34^ In dogs, LJL143 elicits strong DTH and IFN-γ responses in the skin and blood following uninfected sand fly challenge.^13^ LJL143 exhibits adjuvant-like activity in mice, where priming immunized animals with unadjuvanted LJL143 induced higher CD4+ T cell proliferative responses to *Leishmania* antigens in a virus-like particle (VLP) vaccine compared to mice that did not receive LJL143.^31^ Despite this immunogenicity, LJL143 has shown only partial or no protection in mice challenged with *L. major* co-inoculated with saliva^35^ or infected sand fly bite.^36^ However, our data from human PBMCs show that LJL143 promotes the killing of *L. infantum* by infected macrophages *ex vivo*. This contrast highlights the importance of assessing these salivary proteins in a human immune context, where their activity may differ markedly from what is observed in preclinical models.

In participants exposed to *Lu. longipalpis*, treatment of PBMCs with SGE induced a mixed T_H_1/T_H_2 response, while LJM19 and LJL143 both induced T_H_1 responses. LJM19 was characterized by high expression of IL-12, whereas LJL143 showed high expression of both IFN-γ and IL-12. Classically, IL-12 is secreted by professional antigen presenting cells and regulates T_H_1 lineage commitment, leading to IFN-γ production by T cells.^18^ However, as seen with LJM19, strong induction of IL-4 can suppress IFN-γ despite high levels of IL-12.^37^ This co-expression of IL-12 and IL-4 mirrors the cytokine profile seen in LJM19-immunized hamsters protected against *L. donovani* challenge,^32^ suggesting that protection from leishmaniasis is not solely dependent on IFN-γ production but rather on the balance of T_H_ cytokines.

Of the T_H_2 cytokines, IL-13 was often expressed at levels equal to or higher than IFN-γ. IL-13 promotes *Leishmania* infection and exacerbates immunopathology,^38-40^ in part by inhibiting expression of IL12Rβ2 which transduces critical signals for T_H_1 differentiation.^41,42^ Polymorphisms at the *IL13* locus in humans have also been associated with susceptibility to CL caused by *Leishmania guyanensis*.^43^ In a naturally exposed population in Mali, IL-13 was highly expressed in PBMCs stimulated with SGE from *P. duboscqi*, but only at low levels in the skin after uninfected bite challenge.^5^ Further studies are needed to determine whether the high IL-13 expression induced by LJM19 and LJL143 reflects an intrinsic property of these proteins or a counterregulatory response to T_H_1 polarization.

We found that LJM19 induces IL-6 and IL-7, while both LJM19 and LJL143 induce IL-1β and the type I interferon, IFN-α. This contrasts with the reported downregulation of IL-6 and IL-1β by PpSP32, another sand fly salivary protein.^44^ To our knowledge, this is the first study to report altered expression of IL-7 and IFN-α in response to specific sand fly salivary proteins. IL-6 exerts pleiotropic immune effects, including activation of the acute phase response, granulopoiesis, B cell proliferation, and CD8+ T effector cell development.^45^ In mice, IL-6 has been shown to facilitate resistance to *L. major*^46^ and *L. donovani*^47^, in the latter case by inhibiting the proliferation of IL-10-expressing CD4+ T cells. IL-7 supports the survival, proliferation, and maintenance of T cells in peripheral tissues,^19^ including skin resident memory T cells,^48^ which are critical for sustained protection against *Leishmania*.^49^ While IL-1β has been reported to protect against *L. amazonensis*,^50^ most studies suggest IL-1β exacerbates parasite dissemination and immunopathology.^51-55^ Similarly, IFN-α likely benefits the parasite by antagonizing IFN-γ responses and suppressing the development of *Leishmania*-specific T cells,^56,57^ though the timing of IFN-α signaling relative to *Leishmania* infection can shift the balance between protection and susceptibility.^58^

Unexpectedly, even in individuals unexposed to *Lu. longipalpis*, treatment of PBMCs with LJM19 or LJL143 decreased the percentage of *Leishmania*-infected macrophages in co-culture to a magnitude similar to that observed for exposed individuals. This suggests that LJM19 and LJL143 have intrinsic adjuvant-like properties to activate innate immune pathways, even in the absence of prior sand fly exposure. Considering the T_H_1-polarized profiles induced by LJM19 and LJL143, we propose that adaptive immunity in exposed individuals works in concert with innate immunity to optimize the anti-*Leishmania* response. Identifying the innate immune receptors and cell subsets that mediate the adjuvant-like effects of LJM19 and LJL143 remains an area for future research. The similar number of amastigotes per infected macrophage between media-treated versus LJM19 or LJL143-treated samples suggests that these salivary proteins act early in macrophage infection, either by blocking parasite invasion or by enhancing killing of *Leishmania* shortly after invasion, before the parasite establishes in the phagolysosome.

Our study has several limitations. The cohort is demographically homogenous (80% male, 73% White), limiting generalizability. Due to limited PBMC recovery at some blood collections, each assay used PBMCs from more than one time point. While we cannot exclude the possibility that participants were previously exposed to local *Lutzomyia* species at low prevalence,^59^ all participants and blood bank samples were screened and found to be negative for IgG against *Lu. longipalpis* SGE. Finally, most of our findings are based on PBMC responses and need reinforcement through studies of immune cells in the skin.

In summary, we report that humans exposed to *Lu. longipalpis* generate robust innate and adaptive cellular immune responses to the sand fly salivary proteins LJM19 and LJL143. These proteins exhibit adjuvant-like activity, induce T_H_1 polarization, and enhance the protection of macrophages against *Leishmania* infection. This highlights the potential of leveraging ant-isaliva immunity in humans to protect against vector borne pathogens. Furthermore, we posit that chronic immune stimulation by vector bites is underappreciated as an environmental exposure that profoundly reshapes human immune responses in endemic populations. Our work underscores the importance of human vector challenge studies as an approach to investigate how repeated exposure to vector salivary antigens impacts human immunity, a daily occurrence for the approximately 350 million people who live in regions where sand flies and other disease vectors are prevalent.

## METHODS

### Study approval

The Institutional Review Boards of Walter Reed Army Medical Center, Walter Reed National Military Medical Center (protocol number WR355023), the National Institute of Allergy and Infectious Diseases (NIAID), and the Uniformed Services University of the Health Sciences (USUHS) approved this study. All human subjects research was conducted in accordance with the principles of the Declaration of Helsinki. Participants provided written informed consent prior to study participation, including consent for the use of their photographs. The study was registered on www.clinicaltrials.gov as NCT01289977.

### Study population

The screening cohort and enrollment criteria have been published previously for a related study on human immunity to the bites of *Phlebotomus duboscqi*, a sand fly vector of cutaneous leishmaniasis predominantly found in Africa,^10^ however there was no overlap in the enrollment cohort of the prior study and the current one. In this single site study conducted at Walter Reed Army Medical Center (WRAMC), 68 healthy individuals were screened and 15 were enrolled in the *Lu. longipalpis* study cohort. Study participants provided self-identified demographic information, including race and sex, using options defined by the investigator. A medical history and physical examination were obtained and included a review of allergies and travel history. The inclusion criteria were healthy military healthcare beneficiaries between 18 and 50 years old who were willing to remain in the local area for the next 12 months and participate in all study procedures. Exclusion criteria included a history of travel for more than 30 consecutive days to a geographic area where *Lutzomyia longipalpis* is present, positivity by screening ELISA to IgG that bind *Lu. longipalpis* salivary gland extract (SGE), elevated serum IgE >144 kU/L, pregnancy, history of chronic medical illness, large skin reactions to insect bites, problems with prior phlebotomy, or use of medications that may interfere with immune responses.

### Human controlled exposure to uninfected laboratory-reared *Lutzomyia longipalpis*

The colony of *Lu. longipalpis* sand flies used for this study was originally field collected in 2004 in Jacobina, Brazil. For the clinical study, *Lu. longipalpis* were reared in a pathogen-free insectary at the Laboratory of Malaria and Vector Research (LMVR), NIAID, and were maintained as a closed colony. For each participant, 10 female *Lu. longipalpis* sand flies were starved overnight, loaded into a feeding chamber, then transported to WRAMC on the day of the exposure. Each feeding chamber is composed of a sealed Plexiglass capsule with a fine mesh surface (Precision Plastics, Inc.). The feeding chamber was secured to the upper arm of each participant with the mesh side contacting the skin, allowing the sand flies to feed through the mesh. Each sand fly exposure lasted 20 minutes, during which the feeding chamber was lightly covered with fabric to promote a dark feeding environment. Areas of skin with tattoos were avoided for feeding sites. At the end of each exposure, all sand flies were accounted for and examined by microscopy to assess the number of flies that had taken a blood meal.

Bite site skin reactions were observed by study physicians for 10 minutes immediately following the end of the sand fly exposure. Any participants with skin reactions deemed to be large or potentially allergic were observed for a longer period. Bite site photographs were taken and the physical appearance of the bite site rash and any associated symptoms were recorded. Participants were counseled to refrain from using antihistamines or topical steroids for bite site symptoms until consultation with study physicians. Sand fly exposures were performed once every two weeks for exposures #1 through #4, then once every eight weeks for exposures #5 through #9 (Fig. 1). Feeding sites were alternated between different arms on consecutive visits. Blood was collected from participants 7 ± 3 days following each sand fly exposure, at which time the bite site was reassessed for delayed skin reactions.

### Skin histology and immunohistochemistry

Forty-eight hours after their final exposure (#9) to *Lu. longipalpis*, two study participants (#1 and #13) consented to have a 3 mm skin punch biopsy (Miltex sterile skin punch biopsy tool) taken at a bite site from the most recent exposure and a 2 mm punch biopsy from normal appearing skin on the contralateral arm as a negative control. Each biopsy was bisected, with one half stored in 10% buffered formalin for histology and the other half stored in RNAlater (Ambion) for quantitative (real time) RT-PCR.

Embedding of the biopsies as formalin-fixed paraffin-embedded (FFPE) blocks and histological staining was performed by Histoserv (Germantown, Maryland). Five micron-thick tissue sections were stained with hematoxylin and eosin and evaluated by light microscopy. For immunohistochemistry (IHC), primary antibodies against the following targets were used at the dilutions listed: CD3 at 1:100 (Dako #A0452), CD4 at 1:80 (Dako #M7310), CD8 at 1:75 (Dako #M7103) at 1:75, CD20 at 1:300 (Dako #M0755), CD68 at 1:100 (Dako #M0814), and myeloperoxidase (MPO) at 1:400 (Dako#A0398). For secondary antibodies, biotinylated antimouse IgG (1:500) was used to detect primary antibodies binding CD4, CD8, CD20, CD68, and biotinylated anti-rabbit IgG (1:500) was used to detect anti-CD3. Streptavidin-horseradish peroxidase was used to visualize the protein targets. Slide photography was performed using an Olympus DP73 camera microscope BX51 with Cellsens Dimension Olympus software. The percentage of cells positive for each IHC marker was quantitated with ImageJ software.

### Quantitative RT-PCR of skin cytokines

For each biopsy portion stored in RNAlater, RNA was extracted using the RNeasy Fibrous Tissue Mini Kit (Qiagen) and treated with DNase I to remove contaminating genomic DNA. Total RNA (100 ng) was used for cDNA synthesis using the qScript cDNA Supermix (Quanta Biosciences). Absence of genomic DNA contamination was verified by PCR of total RNA. Relative quantification of IFN-γ, IL-12, IL-4, IL-5, and IL-13 was performed in a LightCycler 480 (Roche Applied Science) using the Universal ProbeLibrary system (Roche). Primers and probes were designed using ProbeFinder software (v 2.45, Roche). Relative quantification of target genes normalized to 18S rRNA was performed using the LightCycler 480 software. Cytokine gene expression from the bite site biopsies was then normalized to the skin biopsy from the contralateral arm.

### Preparation of *Lu. longipalpis* salivary gland extract

Salivary gland extract (SGE) was prepared by dissection of salivary glands from seven day old, laboratory-reared, uninfected adult female *Lu. longipalpis*. Glands were homogenized by ultrasonication with a Branson Sonifier 450 for three 30 second cycles then clarified by centrifugation at 10,000 *xg* for 3 min at 4 °C. Supernatant extracts were collected and stored at −80 °C until use.

### Cloning and expression of *Lu. longipalpis* salivary proteins

DNA of the most abundant salivary molecules from *Lu. longipalpis* was amplified by polymerase chain reaction (PCR) using a forward primer derived from the amino-terminal sequence immediately 3’ to the signal peptide sequence and a reverse primer encoding a hexahistidine tag. The PCR conditions were: one hold for 5 min at 94 °C, two cycles of 30 s at 94 °C, 1 min at 46 °C, 1 min at 72 °C and 23 cycles of 30 s at 94 °C, 1 min at 52 °C, 1 min at 72 °C and one hold of 7 min at 72 °C. The PCR product was cloned into the VR2001-TOPO vector as previously described^60^ then sequenced. The VR-2001 plasmid encoding the His-tagged salivary proteins was sent to the Protein Expression Laboratory at NCI Frederick (Frederick, Maryland) for expression in HEK-293F cells. Supernatant was collected after 72 hours and concentrated from 1 L to 300 ml using a stirred ultrafiltration cell unit (Millipore) with an ultrafiltration membrane (Millipore). The volume was restored to 1 L by the addition of 500 mM NaCl and 10 mM Tris, pH 8.0. The protein was purified by HPLC (Biorad, NGC Chromatography System) using two tandem 5 ml HiTrap Chelating HP columns (GE Healthcare) charged with 0.1 M NiSO4. Protein was detected at 280 nm and eluted by an imidazole gradient. Eluted proteins were collected every minute in a 96-well microtiter plate using a BioFrac fraction collector (Biorad). Fractions corresponding to specific absorbance peaks were selected and run on a NuPage Bis-Tris 4–12% Gel (Novex) with MES running buffer under reducing conditions per the manufacturer’s instructions. Afterwards, the gel was stained with 0.025% Coomassie blue to visualize proteins. Specific fractions were selected based on molecular weight and concentrated to 1 ml using an Amicon Ultra Centrifugal Filter (Millipore). The protein sample was then injected into a G2000SW molecular sieving column (Tosoh Biosciences) via a 1 ml loop connected to the HPLC (DIONEX) with phosphate buffered saline (PBS), pH 7.2 as the buffer for further purification steps. Protein was detected by absorbance at 280 nm and the fractions were of interest were collected and pooled as described above. Protein concentration was measured with a NanoDrop ND-1000 spectrophotometer at 280 nm and calculated using the extinction coefficient of the protein. Endotoxin levels were <20 EU/ml for all proteins.

### Blood collection and storage

Blood was collected from each study participant in heparinized Vacutainer tubes (BD Diagnostics). Collections were performed after exposures #2, #4, and #5 through #9 (Fig. 1). Peripheral blood mononuclear cells (PBMCs) were isolated by density gradient centrifugation using a Ficoll-Paque PLUS solution (GE Healthcare). Plasma supernatants were collected and stored at −80 °C. PBMCs were counted, resuspended in fetal bovine serum (FBS) with 10% dimethyl sulfoxide (DMSO) solution, and transferred to cryovials which were slowly cooled to −80 °C overnight in a Mr. Frosty freezing container (Thermo Fisher Scientific) then transferred to liquid nitrogen.

To obtain pre-exposure negative controls, PBMCs were collected from each study participant by apheresis prior to sand fly exposure. However, initial tests of these apheresed PBMCs demonstrated that they were broadly reactive to the majority of recombinant sand fly salivary proteins tested, indicating that the apheresis procedure had caused non-specific activation of these PBMC batches. Thus, we elected to use PBMCs collected from healthy volunteers at the NIH Blood Bank as *Lu. longipalpis* unexposed negative controls in our stimulation assays (Fig. 4 and Fig. 7). Blood from each donor was screened by ELISA to verify they were negative for IgG against *Lu. longipalpis* SGE. All post-exposure blood was collected via conventional venous phlebotomy.

### Peripheral blood mononuclear cell culture and stimulation

For stimulation assays, cryopreserved PBMCs were thawed quickly at 37 °C, diluted in RPMI 1640 medium, then pelleted by centrifugation for 10 min at 350 *xg* at RT. PBMCs were cultured in RPMI 1640 medium supplemented with 10% AB human serum (Sigma), 1% sodium pyruvate, 1% non-essential amino acids, 1% HEPES buffer, 50 μM beta-mercaptoethanol, and 40 mg/ml penicillin/streptomycin in a 5% CO_2_ humidified atmosphere at 37 °C overnight. Cells were counted and viability was assessed using Trypan blue (Hyclone, Thermo Fisher Scientific). PBMCs were then cultured in 96-well plates in cell culture medium at 1 x 10^6^ cells/ml in a final volume of 200 μl per well and incubated with medium alone, SGE (0.5 pairs/ml), 10 μg/ml recombinant *Lu. longipalpis* salivary proteins, or 2.5 μg/ml concavalin A (ConA). Cell supernatants were collected after 96 hours, clarified by centrifugation for 10 min at 350 *xg* at 4 °C, then stored at −80 °C until further use.

Within each assay, PBMCs from each individual were taken from a single time point, though the time point used varied between individuals, depending on the availability of PBMCs. The specific time points used for each assay for each individual are detailed in Table S1.

### Interferon gamma ELISA

ELISA was performed on thawed supernatants of stimulated PBMCs from exposure #2 or #4 (Table S1) using the anti-human interferon gamma ELISA kit (BD Biosciences) according to the manufacturer’s instructions. The results were interpolated from a standard curve using recombinant IFN-γ and expressed as the concentration of IFN-γ (pg/ml) minus the amount of background IFN-γ secreted from cells treated with media alone.

### Cytokine multiplex bead array

Cytokine concentrations in the supernatants of PBMCs from exposure #4 through #9 (Table S1) were measured using the Cytokine Human Magnetic 25-Plex Panel (Invitrogen) according to the manufacturer’s instructions.

### PBMC-macrophage co-culture *Leishmania* killing assay

Human macrophages were derived from PBMCs of exposures #7 through #9 (Table S1) in a subset of study participants (*n* = 4) who completed all nine *Lu. longipalpis* exposures or from NIH blood bank volunteers (*n* = 4). Two technical replicates were performed on PBMCs from each participant, for a total of 8 samples per sand fly exposure group. For differentiation into macrophages, PBMCs were plated in a 16-well chamber slide at 3 x 10^5^ cells per well in the presence of complete RPMI and 20 ng/ml of GM-CSF for 1 hour at 37 °C with 5% CO_2_. Nonadherent cells were removed, then media was replaced with complete RPMI + 20 ng/ml GM-CSF. On day 5, matched donor PBMCs were thawed for use in the autologous killing assay and cultured in suspension in RPMI. On day 6, the cultured macrophages were infected with stationary phase *L*. *infantum* at a parasite:macrophage ratio of 5:1, then incubated for 8 hours at 37 °C with 5% CO_2_. Non-internalized parasites were removed by washing. Autologous PBMCs were treated with media, phytohemagglutinin (PHA) (6.25 μg/ml), or salivary protein (10 μg/ml) for 4 hours at 37 °C with 5% CO_2_, then added to the *Leishmania*-infected macrophages at 5 x 10^5^ PBMCs per well. After 5 days of co-culture, cells were fixed, stained with Giemsa, and assessed by light microscopy. Approximately 300 macrophages were counted per well and evaluated for the number of intracellular *Leishmania* amastigotes.

### Statistics and reproducibility

Statistical analyses were performed in GraphPad Prism (v 10.2.0). Differences were considered statistically significant for p < 0.05 (*). Due to the exploratory nature of our study, we also indicated where p > 0.05 but < 0.10 (denoted by “#”) to identify potential trends that may be biologically relevant but did not achieve the significance threshold.

For the IFN-γ ELISA (Fig. 4), non-parametric statistical tests were used because the dataset failed both normality and lognormality tests. Friedman test with Dunn’s multiple comparisons test was used for pairwise comparisons of IFN-γ concentration between SGE (comparator) and each of the *Lu. longipalpis* recombinant salivary proteins.

For cytokine measurements by multiplex bead array (Fig. 5 and Fig. 6), the distributions of both raw and log2-transformed cytokine concentrations failed normality testing, so nonparametric tests were used for these analyses. For each cytokine, comparisons between the three treatment groups (SGE, LJM19, and LJL143) were performed with the Friedman test (due to having participant-matched samples) and Dunn’s test for multiple comparisons. To test for significant polarization towards T_H_1 or T_H_2, we calculated the log2 of the ratio of each T_H_1 cytokine (IFN-γ or IL-12) to each T_H_2 cytokine (IL-4, IL-5, IL-10, or IL-13), where a value of zero represents the null hypothesis, an equal balance of T_H_1 and T_H_2 cytokines. To each ratio, we then applied a Wilcoxon signed-rank test versus a value of zero to determine whether the cytokine ratio was significantly skewed towards T_H_1 or T_H_2.

For the *Leishmania* killing assay (Fig. 7), within each of the two exposure groups (*Lu. longipalpis* Unexposed or Exposed) pairwise differences were analyzed between PBMCs stimulated with media alone versus each of the other treatment conditions. For the percentage of infected macrophages, the dataset passed all normality tests with participant-matched samples and three missing samples (*n* = 1 from Unexposed/media and *n* = 2 from *Lu. longipalpis* Exposed/media due to random failure of these macrophages to adhere and grow in culture), thus a mixed effects model was applied followed by Dunnett’s test for multiple comparisons. To compare the percentage of infected macrophages between unexposed and exposed samples for LJM19 or LJL143, an unpaired t-test with Welch’s correction was used. For the number of amastigotes per infected macrophage, the dataset failed normality testing, thus the non-parametric Kruskal-Wallis test was used for analysis. Because the log2 T_H_1/T_H_2 cytokine ratios are non-normally distributed, we applied Spearman’s test to determine the correlation between the log2 ratio of T_H_1/T_H_2 cytokines and the percentage of infected macrophages. Correlation trend lines for non-parametric data were plotted using a smoothing spline with 3 knots.

## Supplementary Material

Supplement 1

## Figures and Tables

**Table 1.** Study participant characteristics.

Participant ID	Completed Sand Fly Exposure #	Age	Sex	Race	Allergy History	Baseline Plasma IgE (kU/L)	Reason for Early Withdrawal or Skipped Feedings
1	9	26	M	White	None	17.9	-
2	5	26	M	White	None	9.9	Military transfer orders
3	6	26	M	Asian	Seasonal allergies Asthma	114.0	Discharged from military
4	8	40	M	White	Asthma	6.7	Military transfer orders
5	7	29	M	White	None	3.3	Military transfer orders
6	6	34	M	White	None	19.9	Participant request, adverse event
7	6	45	M	Black	Seasonal allergies Allergic to shellfish Eczema Asthma	20.7	Participant request, adverse event
8	9	22	M	White	None	96.6	-
9	5	26	M	Asian	Seasonal allergies	6.4	Medically advised to stop due to adverse event
10	5	24	M	White	Anaphylaxis to raisins	15.3	Military transfer orders
11	7	46	M	White	Seasonal allergies	<2.0	Two feedings skipped (medical hold, schedule conflict)
12	9	27	F	White	Urticaria to acetaminophen-hydrocodone Bee allergy Asthma	26.0	-
13	9	26	F	Multiracial	Seasonal allergies Angioedema to shellfish	61.1	-
14	9	41	M	White	Seasonal allergies Asthma	77.2	-
15	9	28	F	White	Urticaria to amoxicillin-clavulanic acid	6.7	-

## References

[1] GBDCN. (ed IHME) (Seattle, WA, United States, 2024).

[2] Serafim T. D. Leishmaniasis: the act of transmission. Trends Parasitol 37, 976–987 (2021). 10.1016/j.pt.2021.07.003 34389215

[3] Lakhal-Naouar I. The human immune response to saliva of Phlebotomus alexandri, the vector of visceral leishmaniasis in Iraq, and its relationship to sand fly exposure and infection. PLoS Negl Trop Dis 15, e0009378 (2021). 10.1371/journal.pntd.0009378 34081700 PMC8174707

[4] Vinhas V. Human anti-saliva immune response following experimental exposure to the visceral leishmaniasis vector, Lutzomyia longipalpis. Eur J Immunol 37, 3111–3121 (2007). 10.1002/eji.200737431 17935072

[5] Oliveira F. Delayed-type hypersensitivity to sand fly saliva in humans from a leishmaniasis-endemic area of Mali is Th1-mediated and persists to midlife. J Invest Dermatol 133, 452–459 (2013). 10.1038/jid.2012.315 22992802 PMC3529997

[6] Carvalho A. M. Immune Response to LinB13, a Lutzomyia Intermedia Salivary Protein Correlates With Disease Severity in Tegumentary Leishmaniasis. Clin Infect Dis 75, 1754–1762 (2022). 10.1093/cid/ciac258 35385578 PMC9662176

[7] Mondragon-Shem K. Severity of old world cutaneous leishmaniasis is influenced by previous exposure to sandfly bites in Saudi Arabia. PLoS Negl Trop Dis 9, e0003449 (2015). 10.1371/journal.pntd.0003449 25646796 PMC4315490

[8] Bourke C. D., Berkley J. A. & Prendergast A. J. Immune Dysfunction as a Cause and Consequence of Malnutrition. Trends Immunol 37, 386–398 (2016). 10.1016/j.it.2016.04.003 27237815 PMC4889773

[9] Natukunda A. The effect of helminth infection on vaccine responses in humans and animal models: A systematic review and meta-analysis. Parasite Immunol 44, e12939 (2022). 10.1111/pim.12939 35712983 PMC9542036

[10] de Araujo F. F. Immune response profiles from humans experimentally exposed to Phlebotomus duboscqi bites. Front Immunol 15, 1335307 (2024). 10.3389/fimmu.2024.1335307 38633260 PMC11021656

[11] Guerrero D. Evaluation of cutaneous immune response in a controlled human in vivo model of mosquito bites. Nat Commun 13, 7036 (2022). 10.1038/s41467-022-34534-9 36396947 PMC9672097

[12] Lerner E. A., Ribeiro J. M., Nelson R. J. & Lerner M. R. Isolation of maxadilan, a potent vasodilatory peptide from the salivary glands of the sand fly Lutzomyia longipalpis. J Biol Chem 266, 11234–11236 (1991). 2040631

[13] Collin N. Sand fly salivary proteins induce strong cellular immunity in a natural reservoir of visceral leishmaniasis with adverse consequences for Leishmania. PLoS Pathog 5, e1000441 (2009). 10.1371/journal.ppat.1000441 19461875 PMC2677456

[14] Gomes R. Immunity to a salivary protein of a sand fly vector protects against the fatal outcome of visceral leishmaniasis in a hamster model. Proc Natl Acad Sci U S A 105, 7845–7850 (2008). 10.1073/pnas.0712153105 18509051 PMC2397325

[15] Oliveira F., Lawyer P. G., Kamhawi S. & Valenzuela J. G. Immunity to distinct sand fly salivary proteins primes the anti-Leishmania immune response towards protection or exacerbation of disease. PLoS Negl Trop Dis 2, e226 (2008). 10.1371/journal.pntd.0000226 18414648 PMC2291569

[16] Oliveira F. A sand fly salivary protein vaccine shows efficacy against vector-transmitted cutaneous leishmaniasis in nonhuman primates. Sci Transl Med 7, 290ra290 (2015). 10.1126/scitranslmed.aaa3043 26041707

[17] Tavares N. M. Lutzomyia longipalpis saliva or salivary protein LJM19 protects against Leishmania braziliensis and the saliva of its vector, Lutzomyia intermedia. PLoS Negl Trop Dis 5, e1169 (2011). 10.1371/journal.pntd.0001169 21655303 PMC3104964

[18] Zundler S. & Neurath M. F. Interleukin-12: Functional activities and implications for disease. Cytokine Growth Factor Rev 26, 559–568 (2015). 10.1016/j.cytogfr.2015.07.003 26182974

[19] Winer H. IL-7: Comprehensive review. Cytokine 160, 156049 (2022). 10.1016/j.cyto.2022.156049 36201890

[20] Antonelli L. R. Disparate immunoregulatory potentials for double-negative (CD4−CD8−) alpha beta and gamma delta T cells from human patients with cutaneous leishmaniasis. Infect Immun 74, 6317–6323 (2006). 10.1128/IAI.00890-06 16923794 PMC1695524

[21] Ferraz R. CD3(+)CD4(neg)CD8(neg) (double negative) T lymphocytes and NKT cells as the main cytotoxic-related-CD107a(+) cells in lesions of cutaneous leishmaniasis caused by Leishmania (Viannia) braziliensis. Parasit Vectors 10, 219 (2017). 10.1186/s13071-017-2152-2 28468680 PMC5415843

[22] Mou Z. MHC class II restricted innate-like double negative T cells contribute to optimal primary and secondary immunity to Leishmania major. PLoS Pathog 10, e1004396 (2014). 10.1371/journal.ppat.1004396 25233487 PMC4169504

[23] Theodor O. A study of the reaction to phlebotomus bites with some remarks on “Harara”. Trans R Soc Trop Med Hyg 29, 273–284 (1935). 10.1016/S0035-9203(35)90090-6

[24] Alexander J. O. Papular urticaria and immune complexes. J Am Acad Dermatol 12, 374–375 (1985). 10.1016/s0190-9622(85)80065-7 3973134

[25] Strobl J. & Haniffa M. Functional heterogeneity of human skin-resident memory T cells in health and disease. Immunol Rev 316, 104–119 (2023). 10.1111/imr.13213 37144705 PMC10952320

[26] Vogt M. B. Mosquito saliva alone has profound effects on the human immune system. PLoS Negl Trop Dis 12, e0006439 (2018). 10.1371/journal.pntd.0006439 29771921 PMC5957326

[27] Ariotti S. T cell memory. Skin-resident memory CD8(+) T cells trigger a state of tissue-wide pathogen alert. Science 346, 101–105 (2014). 10.1126/science.1254803 25278612

[28] Kadoki M. Organism-Level Analysis of Vaccination Reveals Networks of Protection across Tissues. Cell 171, 398–413 e321 (2017). 10.1016/j.cell.2017.08.024 28942919 PMC7895295

[29] Asojo O. A. Structure of SALO, a leishmaniasis vaccine candidate from the sand fly Lutzomyia longipalpis. PLoS Negl Trop Dis 11, e0005374 (2017). 10.1371/journal.pntd.0005374 28278244 PMC5344329

[30] Ferreira V. P. SALO, a novel classical pathway complement inhibitor from saliva of the sand fly Lutzomyia longipalpis. Sci Rep 6, 19300 (2016). 10.1038/srep19300 26758086 PMC4725370

[31] Cecilio P. Pre-clinical antigenicity studies of an innovative multivalent vaccine for human visceral leishmaniasis. PLoS Negl Trop Dis 11, e0005951 (2017). 10.1371/journal.pntd.0005951 29176865 PMC5720812

[32] Fiuza J. A. Intradermal Immunization of Leishmania donovani Centrin Knock-Out Parasites in Combination with Salivary Protein LJM19 from Sand Fly Vector Induces a Durable Protective Immune Response in Hamsters. PLoS Negl Trop Dis 10, e0004322 (2016). 10.1371/journal.pntd.0004322 26752686 PMC4708988

[33] Collin N. Lufaxin, a novel factor Xa inhibitor from the salivary gland of the sand fly Lutzomyia longipalpis blocks protease-activated receptor 2 activation and inhibits inflammation and thrombosis in vivo. Arterioscler Thromb Vasc Biol 32, 2185–2198 (2012). 10.1161/ATVBAHA.112.253906 22796577 PMC3421056

[34] Mendes-Sousa A. F. The Sand Fly Salivary Protein Lufaxin Inhibits the Early Steps of the Alternative Pathway of Complement by Direct Binding to the Proconvertase C3b-B. Front Immunol 8, 1065 (2017). 10.3389/fimmu.2017.01065 28912782 PMC5583147

[35] Xu X. Structure and function of a "yellow" protein from saliva of the sand fly Lutzomyia longipalpis that confers protective immunity against Leishmania major infection. J Biol Chem 286, 32383–32393 (2011). 10.1074/jbc.M111.268904 21795673 PMC3173228

[36] Cecilio P. Engineering a vector-based pan-Leishmania vaccine for humans: proof of principle. Sci Rep 10, 18653 (2020). 10.1038/s41598-020-75410-0 33122717 PMC7596519

[37] Schmitt E., Rude E. & Germann T. The immunostimulatory function of IL-12 in T-helper cell development and its regulation by TGF-beta, IFN-gamma and IL-4. Chem Immunol 68, 70–85 (1997). 10.1159/000058695 9329217

[38] Castilho T. M. Murine model of chronic L. (Viannia) panamensis infection: role of IL-13 in disease. Eur J Immunol 40, 2816–2829 (2010). 10.1002/eji.201040384 20827674 PMC3289133

[39] Matthews D. J. IL-13 is a susceptibility factor for Leishmania major infection. J Immunol 164, 1458–1462 (2000). 10.4049/jimmunol.164.3.1458 10640762

[40] Zaatar M. T., Simaan Y. & Karam M. C. Exogenous IL-13 exacerbates Leishmania major infection and abrogates acquired immunity to re-infection. Parasitol Res 121, 2009–2017 (2022). 10.1007/s00436-022-07539-y 35536514

[41] Alexander J. An essential role for IL-13 in maintaining a non-healing response following Leishmania mexicana infection. Eur J Immunol 32, 2923–2933 (2002). 10.1002/1521-4141(2002010)32:10<2923::AID-IMMU2923>3.0.CO;2-E 12355446

[42] Bourreau E., Prevot G., Pradinaud R. & Launois P. Interleukin (IL)-13 is the predominant Th2 cytokine in localized cutaneous leishmaniasis lesions and renders specific CD4+ T cells unresponsive to IL-12. J Infect Dis 183, 953–959 (2001). 10.1086/319249 11237813

[43] Junior J. A fine mapping of single nucleotide variants and haplotype analysis of IL13 gene in patients with Leishmania guyanensis-cutaneous leishmaniasis and plasma cytokines IL-4, IL-5, and IL-13. Front Immunol 14, 1232488 (2023). 10.3389/fimmu.2023.1232488 37908348 PMC10613733

[44] Souissi C. PpSP32, the Phlebotomus papatasi immunodominant salivary protein, exerts immunomodulatory effects on human monocytes, macrophages, and lymphocytes. Parasit Vectors 16, 1 (2023). 10.1186/s13071-022-05627-7 36593519 PMC9806891

[45] Tanaka T., Narazaki M. & Kishimoto T. IL-6 in inflammation, immunity, and disease. Cold Spring Harb Perspect Biol 6, a016295 (2014). 10.1101/cshperspect.a016295 25190079 PMC4176007

[46] Ehrchen J. M. Keratinocytes determine Th1 immunity during early experimental leishmaniasis. PLoS Pathog 6, e1000871 (2010). 10.1371/journal.ppat.1000871 20442861 PMC2861693

[47] Stager S. Distinct roles for IL-6 and IL-12p40 in mediating protection against Leishmania donovani and the expansion of IL-10+ CD4+ T cells. Eur J Immunol 36, 1764–1771 (2006). 10.1002/eji.200635937 16791879 PMC2659577

[48] Adachi T. Hair follicle-derived IL-7 and IL-15 mediate skin-resident memory T cell homeostasis and lymphoma. Nat Med 21, 1272–1279 (2015). 10.1038/nm.3962 26479922 PMC4636445

[49] Scott P. Long-Lived Skin-Resident Memory T Cells Contribute to Concomitant Immunity in Cutaneous Leishmaniasis. Cold Spring Harb Perspect Biol 12 (2020). 10.1101/cshperspect.a038059 PMC7528853 32839202

[50] Lima-Junior D. S. Inflammasome-derived IL-1beta production induces nitric oxide-mediated resistance to Leishmania. Nat Med 19, 909–915 (2013). 10.1038/nm.3221 23749230

[51] Charmoy M. The Nlrp3 inflammasome, IL-1beta, and neutrophil recruitment are required for susceptibility to a nonhealing strain of Leishmania major in C57BL/6 mice. Eur J Immunol 46, 897–911 (2016). 10.1002/eji.201546015 26689285 PMC4828310

[52] Dey R. Gut Microbes Egested during Bites of Infected Sand Flies Augment Severity of Leishmaniasis via Inflammasome-Derived IL-1beta. Cell Host Microbe 23, 134–143 e136 (2018). 10.1016/j.chom.2017.12.002 29290574 PMC5832060

[53] Fernandez-Figueroa E. A. Disease severity in patients infected with Leishmania mexicana relates to IL-1beta. PLoS Negl Trop Dis 6, e1533 (2012). 10.1371/journal.pntd.0001533 22629474 PMC3358333

[54] Novais F. O. CD8+ T cell cytotoxicity mediates pathology in the skin by inflammasome activation and IL-1beta production. PLoS Pathog 13, e1006196 (2017). 10.1371/journal.ppat.1006196 28192528 PMC5325592

[55] Santos D. IL-1beta Production by Intermediate Monocytes Is Associated with Immunopathology in Cutaneous Leishmaniasis. J Invest Dermatol 138, 1107–1115 (2018). 10.1016/j.jid.2017.11.029 29246797 PMC5912958

[56] Kumar R. Type I Interferons Suppress Anti-parasitic Immunity and Can Be Targeted to Improve Treatment of Visceral Leishmaniasis. Cell Rep 30, 2512–2525 e2519 (2020). 10.1016/j.celrep.2020.01.099 32101732 PMC7981274

[57] Xin L. Type I IFN receptor regulates neutrophil functions and innate immunity to Leishmania parasites. J Immunol 184, 7047–7056 (2010). 10.4049/jimmunol.0903273 20483775 PMC4159077

[58] Mattner J. Regulation of type 2 nitric oxide synthase by type 1 interferons in macrophages infected with Leishmania major. Eur J Immunol 30, 2257–2267 (2000). 10.1002/1521-4141(2000)30:8<2257::AID-IMMU2257>3.0.CO;2-U 10940917

[59] Haddow A. D., Curler G. & Moulton J. K. New records of Lutzomyia shannoni and Lutzomyia vexator (Diptera: Psychodidae) in eastern Tennessee. J Vector Ecol 33, 393–396 (2008). 10.3376/1081-1710-33.2.393 19263861

[60] Oliveira F. From transcriptome to immunome: identification of DTH inducing proteins from a Phlebotomus ariasi salivary gland cDNA library. Vaccine 24, 374–390 (2006). 10.1016/j.vaccine.2005.07.085 16154670

