# Supplementary material for: Human sand fly challenge elicits saliva-specific innate and TH1-polarized immunity that promotes Leishmania killing
Source: bioRxiv. 2025 Sep 30:2025.02.25.640210. Originally published 2025 Mar 4. Preprint. [Version 3] doi: 10.1101/2025.02.25.640210 (PMC11974753; doi:10.1101/2025.02.25.640210)
Supplement: Supplement 1 [file media-1.pdf]

## SUPPLEMENTARY FIGURES AND TABLE

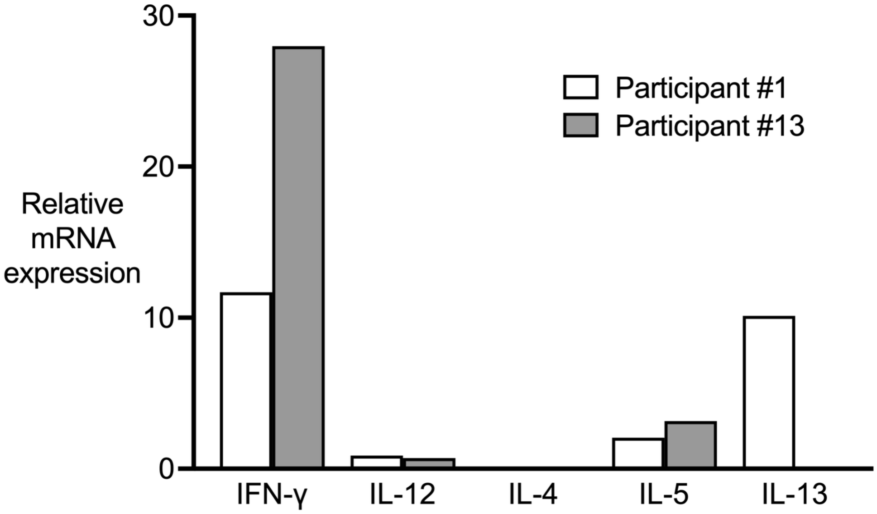


**Fig. S1.** Skin cytokine profiles of the delayed-type hypersensitivity response to *Lu. longipalpis* bites. Skin punch biopsies were collected as described in Fig. 3 for measurement of cytokine mRNA expression by quantitative RT-PCR. For each participant, gene expression at the bite site was normalized to expression in normal appearing skin from the contralateral arm.

**Supplemental Table 1.** PBMC batches by exposure number used for each experiment.

| Participant # | Figure 4 | Figures 5 and 6 | Figure 7 |
|---|---|---|---|
| **1** | 2 | 9 | 7, 8, 9 |
| **2** | 2 | 4 | - |
| **3** | 2 | 9 | - |
| **4** | 2 | 8 | - |
| **5** | 2 | 4 | - |
| **6** | 2 | 6 | - |
| **7** | 4 | 4 | - |
| **8** | 4 | 9 | - |
| **9** | 4 | 5 | - |
| **10** | 4 | 7 | - |
| **11** | 4 | 4 | - |
| **12** | 4 | 5 | 8 |
| **13** | 2 | 6 | 9 |
| **14** | 2 | 5 | 9 |
| **15** | 2 | 5 | - |

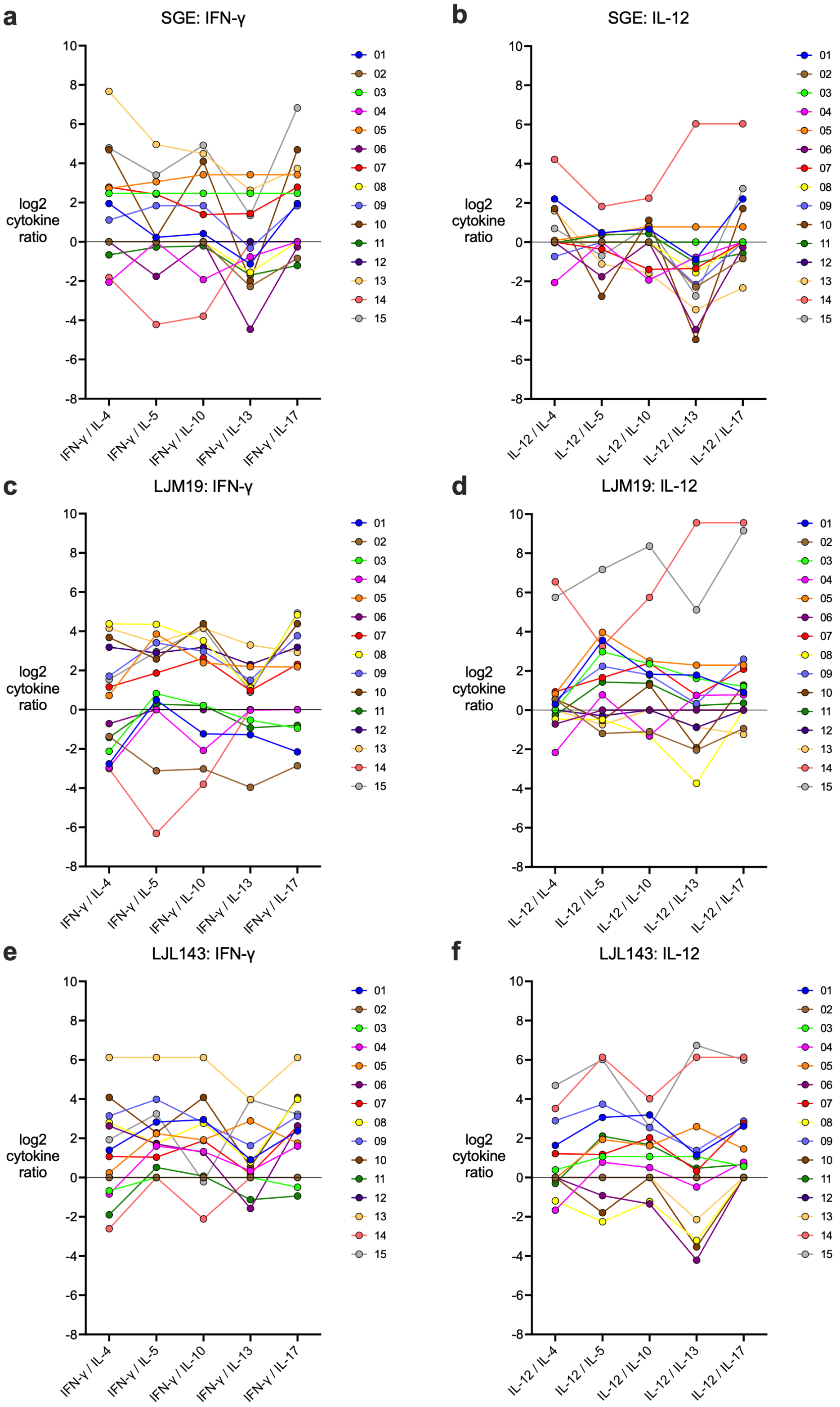


**Fig. S2.** Individual $T_H$ cytokine response profiles to *Lu. longipalpis* SGE and salivary proteins. Re-plot of data from Fig. 5 of cytokines produced by PBMCs from *Lu. longipalpis* exposed participants following stimulation with SGE (**a, b**), LJM19 (**c, d**), or LJL143 (**e, f**). Each color and line and their connecting points represent a single individual.

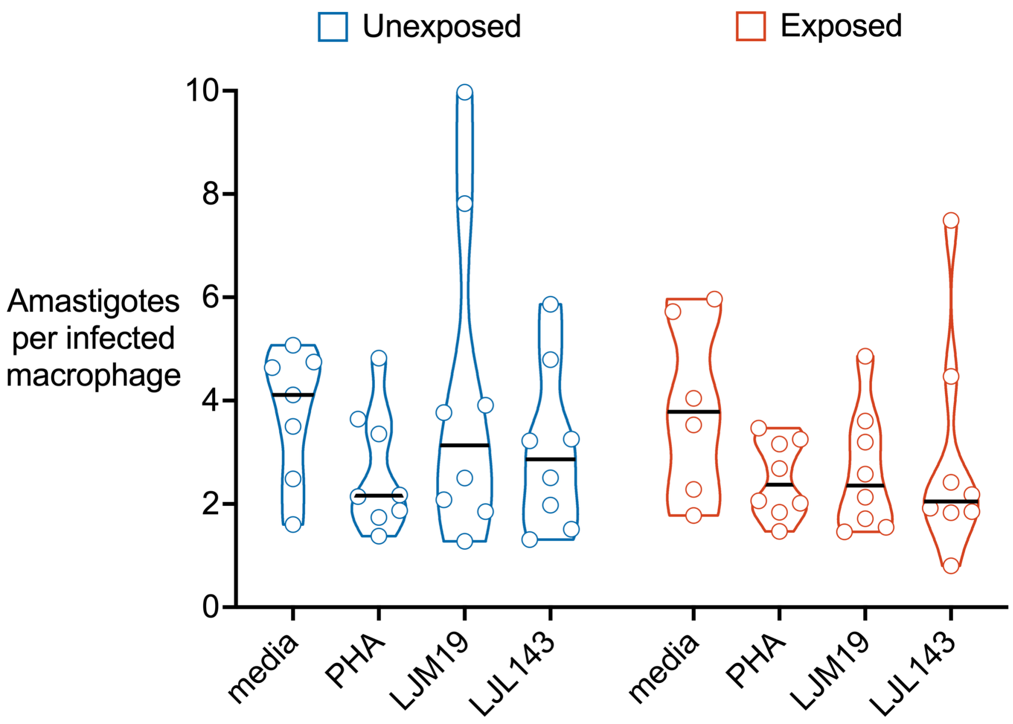


**Fig. S3.** Stimulation with LJM19 or LJL143 has no effect on the number of amastigotes per infected macrophage. Co-culture of PBMC-derived macrophages with stimulated PBMCs was performed as described in Fig. 7. After 5 days of co-culture, the number of amastigotes per infected macrophage was quantified by manual counting of Giemsa-stained cells by light microscopy. Black bar indicates the median. Differences between treatment groups were analyzed via Kruskal-Wallis test. All $p$-values were > 0.05.

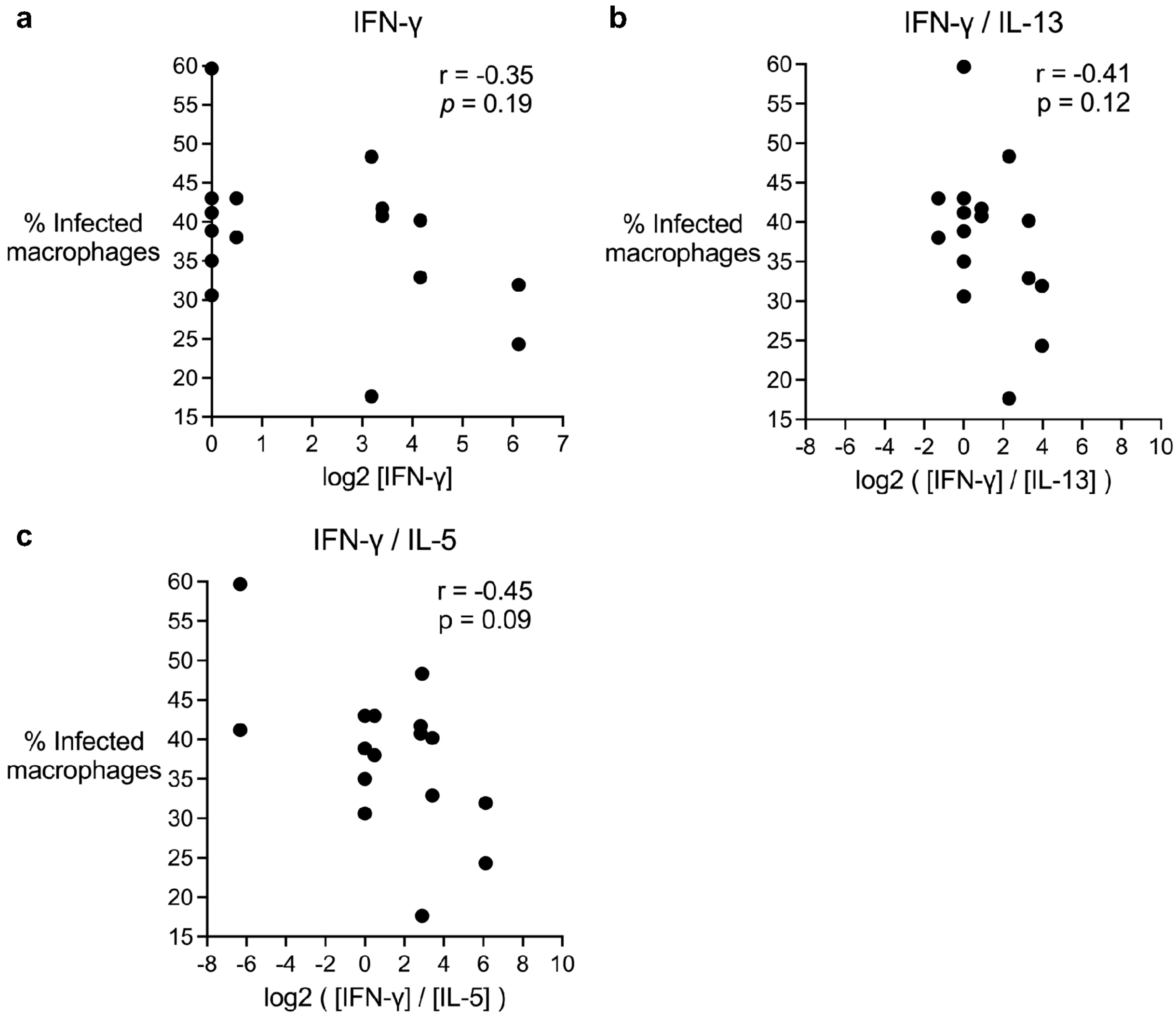


**Fig S4.** Correlation of $T_H$ cytokines with macrophage killing of *Leishmania* parasites. Spearman correlation between IFN-γ alone (**a**), IFN-γ/IL-13 ratio (**b**), or IFN-γ/IL-5 ratio (**c**) as calculated in Fig. 5 and the percentage of infected macrophages for LJM19- and LJL143-treated samples.
