# Supplementary material for: Human sand fly challenge elicits saliva-specific innate and TH1-polarized immunity that promotes Leishmania killing
Source: bioRxiv. 2025 Apr 15:2025.02.25.640210. Originally published 2025 Mar 4. Preprint. [Version 2] doi: 10.1101/2025.02.25.640210 (PMC11974753; doi:10.1101/2025.02.25.640210)
Supplement: Supplementary file 1 [file media-1.pdf]

SUPPLEMENTARY METHODS

Study population

The screening cohort and enrollment criteria have been published previously for a related study on human immunity to the bites of *Phlebotomus duboscqi*, a sand fly vector of cutaneous leishmaniasis predominantly found in Africa,[1] however there was no overlap in the enrollment cohort of the prior study and the current one. In this single site study conducted at Walter Reed Army Medical Center (WRAMC), 68 healthy individuals were screened and 15 were enrolled in the *Lu. longipalpis* study cohort. Study participants provided self-identified demographic information, including race and sex, using options defined by the investigator. A medical history and physical examination were obtained and included a review of allergies and travel history. The inclusion criteria were healthy military healthcare beneficiaries between 18 and 50 years old who were willing to remain in the local area for the next 12 months and participate in all study procedures. Exclusion criteria included a history of travel for more than 30 consecutive days to a geographic area where *Lutzomyia longipalpis* is present, positivity by screening ELISA to IgG that bind *Lu. longipalpis* salivary gland extract (SGE), elevated serum IgE >144 kU/L, pregnancy, history of chronic medical illness, large skin reactions to insect bites, problems with prior phlebotomy, or use of medications that may interfere with immune responses.

Human controlled exposure to uninfected laboratory-reared *Lutzomyia longipalpis*

The colony of *Lu. longipalpis* sand flies used for this study was originally field collected in Jacobina, Brazil. For the clinical study, *Lu. longipalpis* were reared in a pathogen-free insectary at the Laboratory of Malaria and Vector Research (LMVR), NIAID, and were maintained as a closed colony. For each participant, 10 female *Lu. longipalpis* sand flies were starved overnight, loaded into a feeding chamber, then transported to WRAMC on the day of the exposure. Each feeding chamber is composed of a sealed Plexiglass capsule with a fine mesh surface (Precision Plastics, Inc.). The feeding chamber was secured to the upper arm of each participant with the mesh side contacting the skin, allowing the sand flies to feed through the mesh. Each sand fly exposure lasted 20 minutes, during which the feeding chamber was lightly covered with fabric to promote a dark feeding environment. Areas of skin with tattoos were avoided for feeding sites. At the end of each exposure, all sand flies were accounted for and examined by microscopy to assess the number of flies that had taken a blood meal.

Bite site skin reactions were observed by study physicians for 10 minutes immediately following the end of the sand fly exposure. Any participants with skin reactions deemed to be large or potentially allergic were observed for a longer period. Bite site photographs were taken and the physical appearance of the bite site rash and any associated symptoms were recorded. Participants were counseled to refrain from using antihistamines or topical steroids for bite site symptoms until consultation with study physicians. Sand fly exposures were performed once every two weeks for exposures #1 through #4, then once every eight weeks for exposures #5 through #9 (Fig 1). Feeding sites were alternated between different arms on consecutive visits. Blood was collected from participants 7 ± 3 days following each sand fly exposure, at which time the bite site was reassessed for delayed skin reactions.

Skin histology and immunohistochemistry

Forty-eight hours after their final exposure (#9) to *Lu. longipalpis*, two study participants (#1 and #13) consented to have a 3 mm skin punch biopsy (Miltex sterile skin punch biopsy tool) taken at a bite site from the most recent exposure and a 2 mm punch biopsy from normal appearing skin on the contralateral arm as a negative control. Each biopsy was bisected, with one half stored in 10% buffered formalin for histology and the other half stored in RNAlater (Ambion) for quantitative (real time) RT-PCR.

Embedding of the biopsies as formalin-fixed paraffin-embedded (FFPE) blocks and histological staining was performed by Histoserv (Germantown, Maryland). Five micron-thick tissue sections were stained with hematoxylin and eosin and evaluated by light microscopy. For immunohistochemistry (IHC), primary antibodies against the following targets were used at the dilutions listed: CD3 at 1:100 (Dako #A0452), CD4 at 1:80 (Dako #M7310), CD8 at 1:75 (Dako #M7103) at 1:75, CD20 at 1:300 (Dako #M0755), CD68 at 1:100 (Dako #M0814), and myeloperoxidase (MPO) at 1:400 (Dako#A0398). For secondary antibodies, biotinylated anti-mouse IgG (1:500) was used to detect primary antibodies binding CD4, CD8, CD20, CD68, and biotinylated anti-rabbit IgG (1:500) was used to detect anti-CD3. Streptavidin-horseradish peroxidase was used to visualize the protein targets. Slide photography was performed using an Olympus DP73 camera microscope BX51 with Cellsens Dimension Olympus software. The percentage of cells positive for each IHC marker was quantitated with ImageJ software.

Quantitative RT-PCR of skin cytokines

For each biopsy portion stored in RNAlater, RNA was extracted using the RNeasy Fibrous Tissue Mini Kit (Qiagen) and treated with DNase I to remove contaminating genomic

DNA. Total RNA (100 ng) was used for cDNA synthesis using the qScript cDNA Supermix (Quanta Biosciences). Absence of genomic DNA contamination was verified by PCR of total RNA. Relative quantification of IFN-γ, IL-12, IL-4, IL-5, and IL-13 was performed in a LightCycler 480 (Roche Applied Science) using the Universal ProbeLibrary system (Roche). Primers and probes were designed using ProbeFinder software (v 2.45, Roche). Relative quantification of target genes normalized to 18S rRNA was performed using the LightCycler 480 software. Cytokine gene expression from the bite site biopsies was then normalized to the skin biopsy from the contralateral arm.

Preparation of *Lu. longipalpis* salivary gland extract

Salivary gland extract (SGE) was prepared by dissection of salivary glands from seven day old, laboratory-reared, uninfected adult female *Lu. longipalpis*. Glands were homogenized by ultrasonication with a Branson Sonifier 450 for three 30 second cycles then clarified by centrifugation at 10,000 *xg* for 3 min at 4 ^{O}C. Supernatant extracts were collected and stored at -80 ^{O}C until use.

Cloning and expression of *Lu. longipalpis* salivary proteins

DNA of the most abundant salivary molecules from *Lu. longipalpis* was amplified by polymerase chain reaction (PCR) using a forward primer derived from the amino-terminal sequence immediately 3' to the signal peptide sequence and a reverse primer encoding a hexa-histidine tag. The PCR conditions were: one hold for 5 min at 94 ^{O}C, two cycles of 30 s at 94 ^{O}C, 1 min at 46 ^{O}C, 1 min at 72 ^{O}C and 23 cycles of 30 s at 94 ^{O}C, 1 min at 52 ^{O}C, 1 min at 72 ^{O}C and one hold of 7 min at 72 ^{O}C. The PCR product was cloned into the VR2001-TOPO vector as previously described[2] then sequenced. The VR-2001 plasmid encoding the His-tagged salivary proteins was sent to the Protein Expression Laboratory at NCI Frederick (Frederick, Maryland) for expression in HEK-293F cells. Supernatant was collected after 72 hours and concentrated from 1 L to 300 ml using a stirred ultrafiltration cell unit (Millipore) with an ultrafiltration membrane (Millipore). The volume was restored to 1 L by the addition of 500 mM NaCl and 10 mM Tris, pH 8.0. The protein was purified by HPLC (Biorad, NGC Chromatography System) using two tandem 5 ml HiTrap Chelating HP columns (GE Healthcare) charged with 0.1 M $NiSO_4$. Protein was detected at 280 nm and eluted by an imidazole gradient. Eluted proteins were collected every minute in a 96-well microtiter plate using a BioFrac fraction collector (Biorad). Fractions corresponding to specific absorbance peaks were selected and run on a NuPage Bis-Tris 4–12% Gel (Novex) with MES running buffer under reducing conditions per the

manufacturer's instructions. Afterwards, the gel was stained with 0.025% Coomassie blue to visualize proteins. Specific fractions were selected based on molecular weight and concentrated to 1 ml using an Amicon Ultra Centrifugal Filter (Millipore). The protein sample was then injected into a G2000SW molecular sieving column (Tosoh Biosciences) via a 1 ml loop connected to the HPLC (DIONEX) with phosphate buffered saline (PBS), pH 7.2 as the buffer for further purification steps. Protein was detected by absorbance at 280 nm and the fractions were of interest were collected and pooled as described above. Protein concentration was measured with a NanoDrop ND-1000 spectrophotometer at 280 nm and calculated using the extinction coefficient of the protein. Endotoxin levels were <20 EU/ml for all proteins.

Blood collection and storage

Blood was collected from each study participant in heparinized Vacutainer tubes (BD Diagnostics). Collections were performed after exposures #2, #4, and #5 through #9 (Fig 1). Peripheral blood mononuclear cells (PBMCs) were isolated by density gradient centrifugation using a Ficoll-Paque PLUS solution (GE Healthcare). Plasma supernatants were collected and stored at -80 ^{O}C. PBMCs were counted, resuspended in fetal bovine serum (FBS) with 10% dimethyl sulfoxide (DMSO) solution, and transferred to cryovials which were slowly cooled to -80 ^{O}C overnight in a Mr. Frosty freezing container (Thermo Fisher Scientific) then transferred to liquid nitrogen.

To obtain pre-exposure negative controls, PBMCs were collected from each study participant by apheresis prior to sand fly exposure. However, initial tests of these apheresed PBMCs demonstrated that they were broadly reactive to the majority of recombinant sand fly salivary proteins tested, indicating that the apheresis procedure had caused non-specific activation of these PBMC batches. Thus, we elected to use PBMCs collected from healthy volunteers at the NIH Blood Bank as *Lu. longipalpis* unexposed negative controls in our stimulation assays (Fig 4 and Fig 7). Blood from each donor was screened by ELISA to verify they were negative for IgG against *Lu. longipalpis* SGE. All post-exposure blood was collected via conventional venous phlebotomy.

Peripheral blood mononuclear cell culture and stimulation

For stimulation assays, cryopreserved PBMCs were thawed quickly at 37 ^{O}C, diluted in RPMI 1640 medium, then pelleted by centrifugation for 10 min at 350 *xg* at RT. PBMCs were cultured in RPMI 1640 medium supplemented with 10% AB human serum (Sigma), 1% sodium pyruvate, 1% non-essential amino acids, 1% HEPES buffer, 50 μM beta-mercaptoethanol, and

40 mg/ml penicillin/streptomycin in a 5% CO_2 humidified atmosphere at 37 ^{O}C overnight. Cells were counted and viability was assessed using Trypan blue (Hyclone, Thermo Fisher Scientific). PBMCs were then cultured in 96-well plates in cell culture medium at 1 x 10^6 cells/ml in a final volume of 200 µl per well and incubated with medium alone, SGE (0.5 pairs/ml), 10 µg/ml recombinant *Lu. longipalpis* salivary proteins, or 2.5 µg/ml concavalin A (ConA). Cell supernatants were collected after 96 hours, clarified by centrifugation for 10 min at 350 *xg* at 4 ^{O}C, then stored at -80 ^{O}C until further use.

Within each assay, PBMCs from each individual were taken from a single time point, though the time point used varied between individuals, depending on the availability of PBMCs. The range of time points used for each assay are detailed in the sections that follow.

Interferon gamma ELISA

ELISA was performed on thawed supernatants of stimulated PBMCs from exposure #2 or #4 using the anti-human interferon gamma ELISA kit (BD Biosciences) according to the manufacturer's instructions. The results were interpolated from a standard curve using recombinant IFN-γ and expressed as the concentration of IFN-γ (pg/ml) minus the amount of background IFN-γ secreted from cells treated with media alone.

Cytokine multiplex bead array

Cytokine concentrations in the supernatants of PBMCs from exposure #4 through #9 were measured using the Cytokine Human Magnetic 25-Plex Panel (Invitrogen) according to the manufacturer's instructions.

PBMC-macrophage co-culture *Leishmania* killing assay

Human macrophages were derived from PBMCs of exposures #7 through #9 in a subset of study participants (*n* = 4) who completed all nine *Lu. longipalpis* exposures or from NIH blood bank volunteers (*n* = 4). Two technical replicates were performed on PBMCs from each participant, for a total of 8 samples per sand fly exposure group. For differentiation into macrophages, PBMCs were plated in a 16-well chamber slide at 3 x 10^5 cells per well in the presence of complete RPMI and 20 ng/ml of GM-CSF for 1 hour at 37 ^{O}C with 5% CO_2. Non-adherent cells were removed, then media was replaced with complete RPMI + 20 ng/ml GM-CSF. On day 5, matched donor PBMCs were thawed for use in the autologous killing assay and cultured in suspension in RPMI. On day 6, the cultured macrophages were infected with stationary phase *L. infantum* at a parasite:macrophage ratio of 5:1, then incubated for 8 hours at

37 ^{O}C with 5% CO_2. Non-internalized parasites were removed by washing. Autologous PBMCs were treated with media, phytohemagglutinin (PHA) (6.25 µg/ml), or salivary protein (10 µg/ml) for 4 hours at 37 ^{O}C with 5% CO_2, then added to the *Leishmania*-infected macrophages at 5 x 10^5 PBMCs per well. After 5 days of co-culture, cells were fixed, stained with Giemsa, and assessed by light microscopy. Approximately 300 macrophages were counted per well and evaluated for the number of intracellular *Leishmania* amastigotes.

Statistics

Statistical analyses were performed in GraphPad Prism (v 10.2.0). For the IFN-γ ELISA (Fig 4), non-parametric statistical tests were used because the dataset failed both normality and lognormality tests. Friedman test with Dunn's multiple comparisons test was used for pairwise comparisons of IFN-γ concentration between SGE (comparator) and each of the *Lu. longipalpis* recombinant salivary proteins.

For cytokine measurements by multiplex bead array (Fig 5 and Fig 6), the distributions of both raw and log2-transformed cytokine concentrations failed normality testing, so nonparametric tests were used for these analyses. For each cytokine, comparisons between the three treatment groups (SGE, LJM19, and LJL143) were performed with the Friedman test (due to having participant-matched samples) and Dunn's test for multiple comparisons. To test for significant polarization towards T_H1 or T_H2, we calculated the log2 of the ratio of each T_H1 cytokine (IFN-γ or IL-12) to each T_H2 cytokine (IL-4, IL-5, IL-10, or IL-13), where a value of zero represents the null hypothesis, an equal balance of T_H1 and T_H2 cytokines. To each ratio, we then applied a Wilcoxon signed-rank test versus a value of zero to determine whether the cytokine ratio was significantly skewed towards T_H1 or T_H2.

For the *Leishmania* killing assay (Fig 7), within each of the two exposure groups (*Lu. longipalpis* Unexposed or Exposed) pairwise differences were analyzed between PBMCs stimulated with media alone versus each of the other treatment conditions. For the percentage of infected macrophages, the dataset passed all normality tests with participant-matched samples and three missing samples (*n* = 1 from Unexposed/media and *n* = 2 from *Lu. longipalpis* Exposed/media due to random failure of these macrophages to adhere and grow in culture), thus a mixed effects model was applied followed by Dunnett's test for multiple comparisons. For the number of amastigotes per infected macrophage, the dataset failed normality testing, thus the non-parametric Kruskal-Wallis test was used for analysis. Because the log2 T_H1/T_H2 cytokine ratios are non-normally distributed, we applied Spearman's test to determine the correlation between the log2 ratio of T_H1/T_H2 cytokines and the percentage of

infected macrophages. Correlation trend lines for non-parametric data were plotted using a smoothing spline with 3 knots.

SUPPLEMENTARY FIGURES

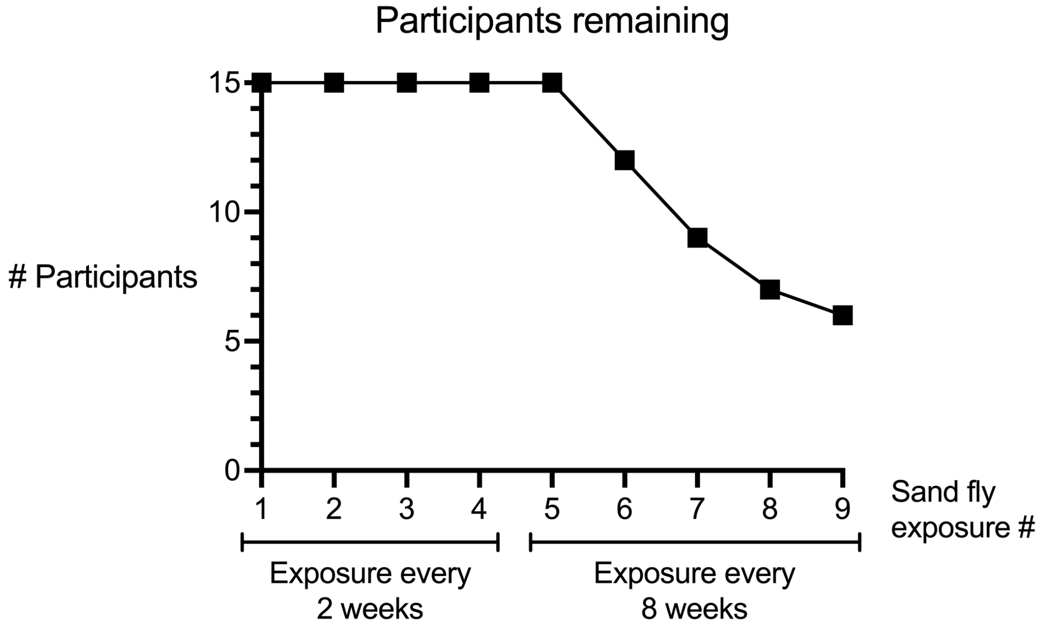

FIG E1. Number of participants remaining at each sand fly exposure.

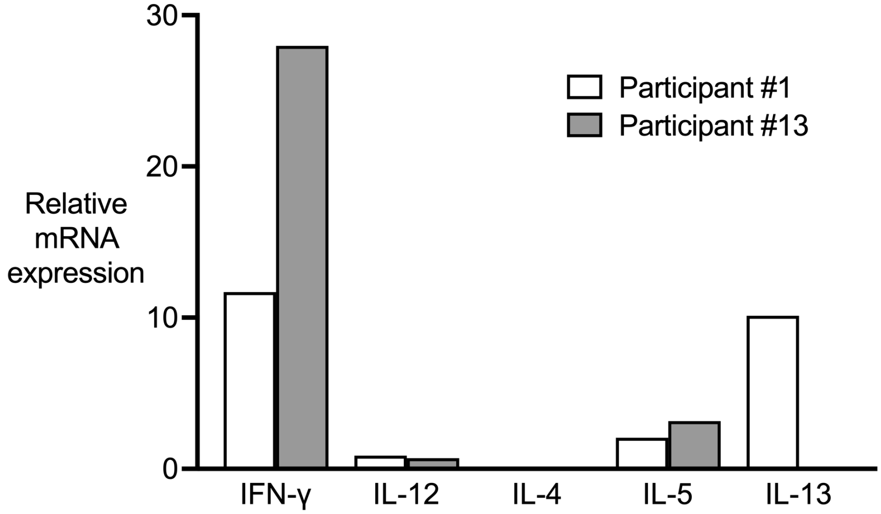

FIG E2. Skin cytokine profiles of the delayed-type hypersensitivity response to *Lu. longipalpis* bites. Skin punch biopsies were collected as described in Fig 3 for measurement of cytokine mRNA expression by quantitative RT-PCR. For each participant, gene expression at the bite site was normalized to expression in normal appearing skin from the contralateral arm.

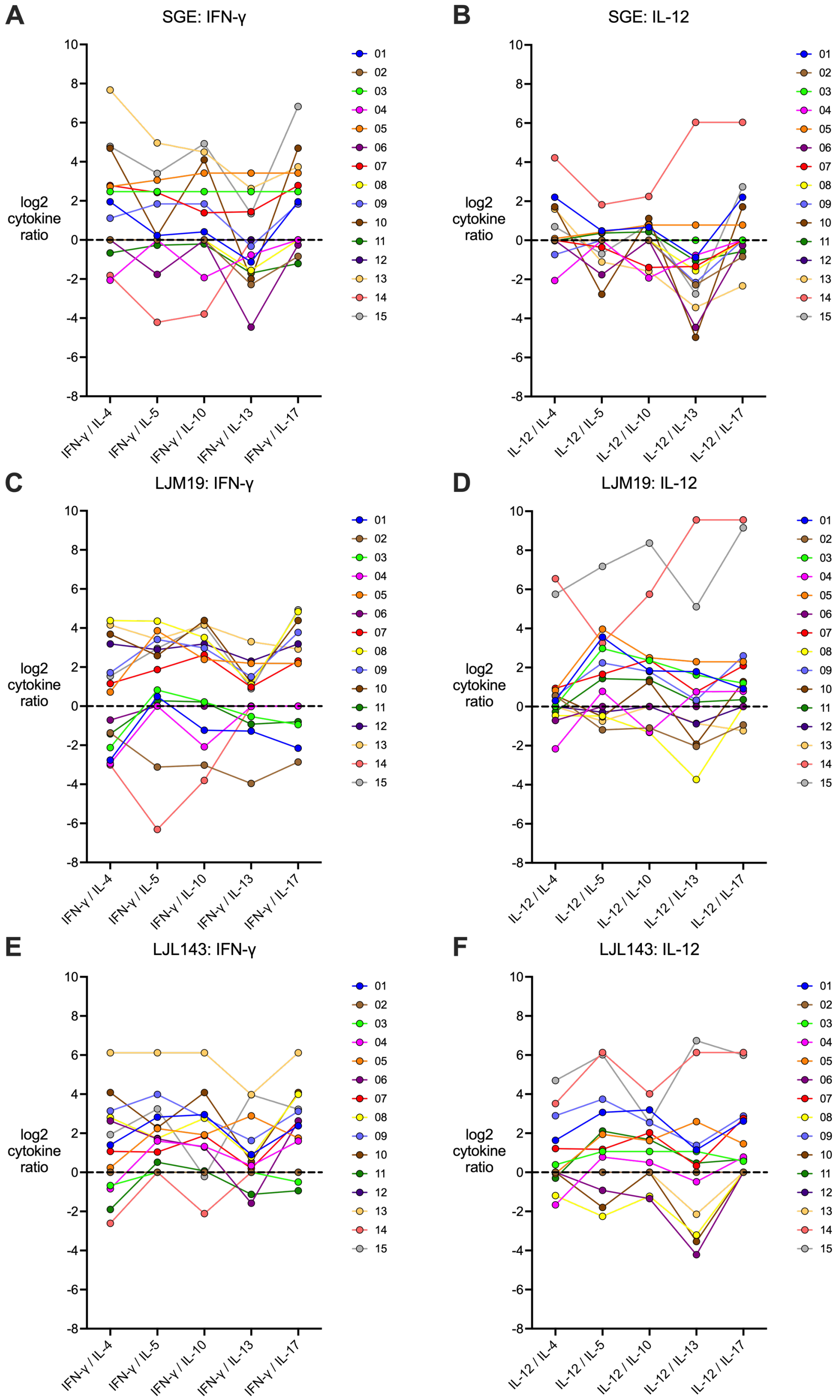

FIG E3. Individual T_H cytokine response profiles to *Lu. longipalpis* SGE and salivary proteins. Replot of data from Fig 5 of cytokines produced by PBMCs from *Lu. longipalpis* exposed participants following stimulation with SGE (**A** and **B**), LJM19 (**C** and **D**), or LJL143 (**E** and **F**). Each color and line and their connecting points represent a single individual.

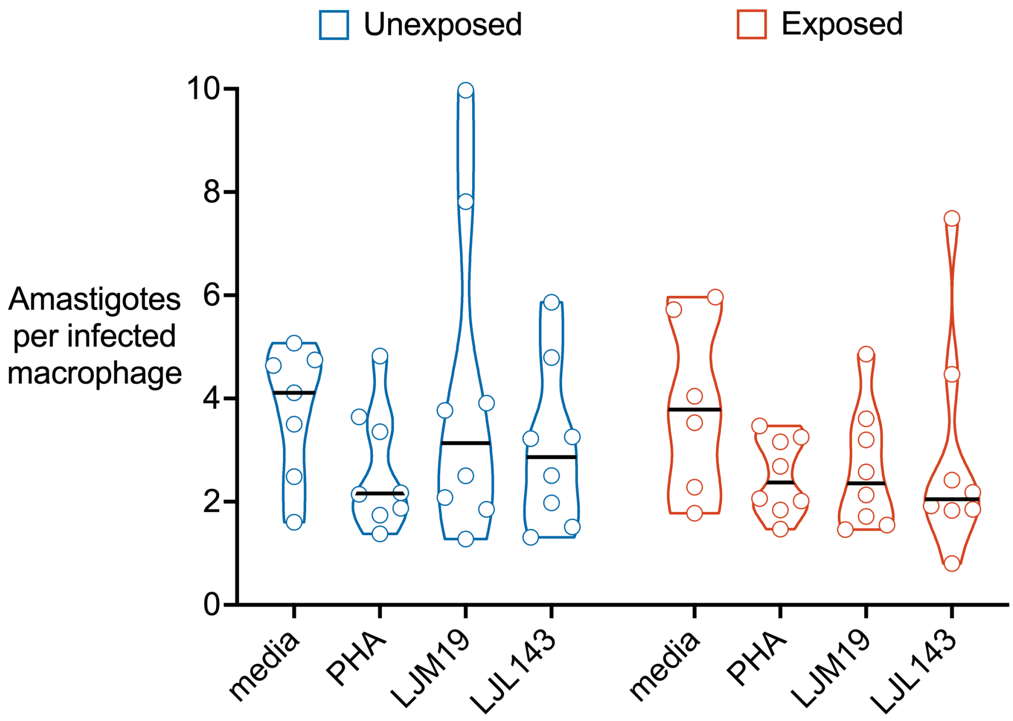

FIG E4. Stimulation with LJM19 or LJL143 has no effect on the number of amastigotes per infected macrophage. Co-culture of PBMC-derived macrophages with stimulated PBMCs was performed as described in Fig 7. After 5 days of co-culture, the number of amastigotes per infected macrophage was quantified by manual counting of Giemsa-stained cells by light microscopy. Black bar indicates the median. Differences between treatment groups were analyzed via Kruskal-Wallis test. All *p*-values were > 0.05.

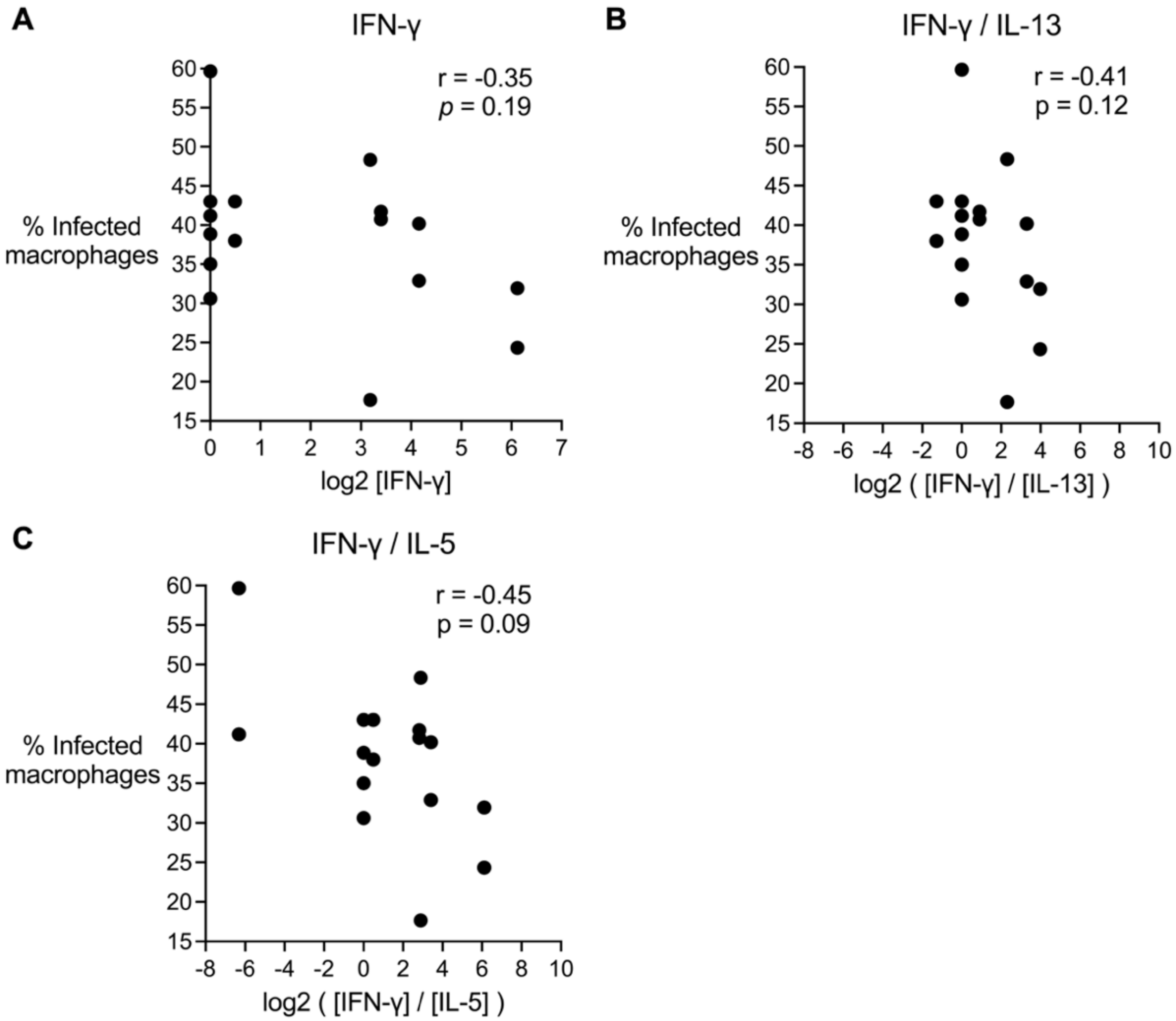

FIG E5. Correlation of T_H cytokines with macrophage killing of *Leishmania* parasites. Spearman correlation between IFN-γ alone (**A**), IFN-γ/IL-13 ratio (**B**), or IFN-γ/IL-5 ratio (**C**) as calculated in Fig 5 and the percentage of infected macrophages for LJM19- and LJL143-treated samples.

SUPPLEMENTARY REFERENCES

1. de Araujo FF, Abdeladhim M, Teixeira C, Hummer K, Wilkerson MD, Ressner R, et al. Immune response profiles from humans experimentally exposed to Phlebotomus duboscqi bites. Front Immunol 2024; 15:1335307.
2. Oliveira F, Kamhawi S, Seitz AE, Pham VM, Guigal PM, Fischer L, et al. From transcriptome to immunome: identification of DTH inducing proteins from a Phlebotomus ariasi salivary gland cDNA library. Vaccine 2006; 24:374-90.
