# Supplementary material for: Lutzomyia longipalpis salivary proteins elicit human innate and adaptive immune responses detrimental to Leishmania parasites
Source: bioRxiv. 2025 Mar 4:2025.02.25.640210. Preprint. [Version 1] doi: 10.1101/2025.02.25.640210 (PMC11974753; doi:10.1101/2025.02.25.640210)
Supplement: Supplementary file 1 [file media-1.pdf]

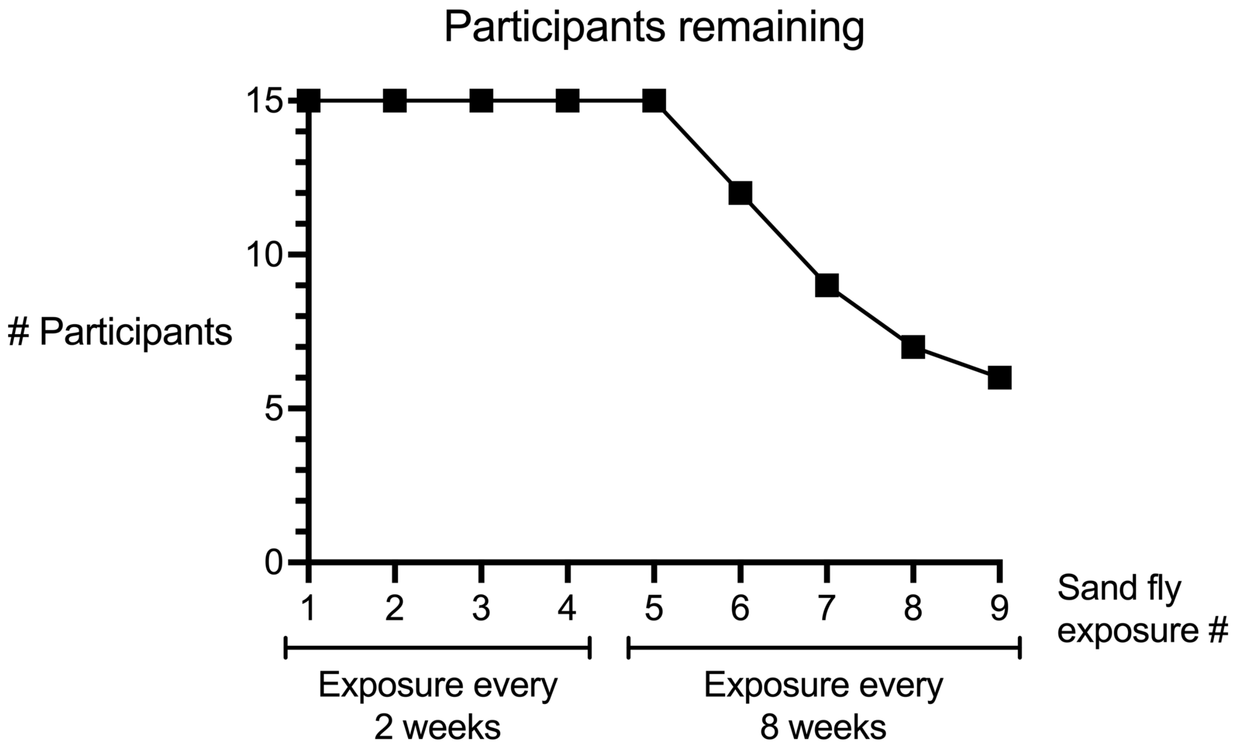

Supplemental Figure 1. Number of participants remaining at each sand fly exposure.

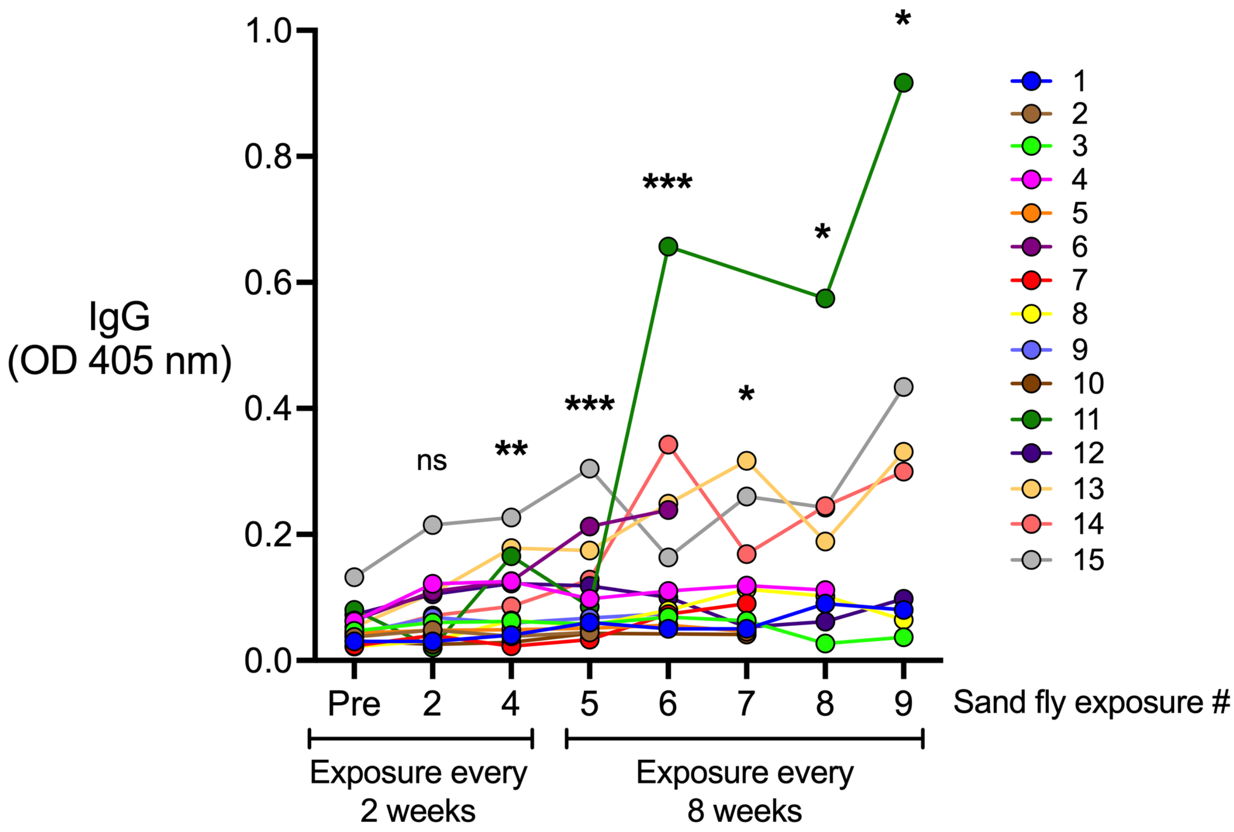

Supplemental Figure 2. Repeated exposure to sand fly bites induces progressively higher levels of anti-SGE IgG in plasma. Plasma was collected ~1 week after the indicated sand fly exposures and the amount of IgG binding *Lu. longipalpis* SGE was measured by ELISA. Each color and line and their connecting points correspond to a single study participant. IgG levels were compared between the pre-exposure plasma (Pre) and each subsequent time point using a mixed effects model with Dunnett's multiple comparisons test, "ns" not significant, * $p < 0.05$, ** $p < 0.01$, *** $p < 0.001$.

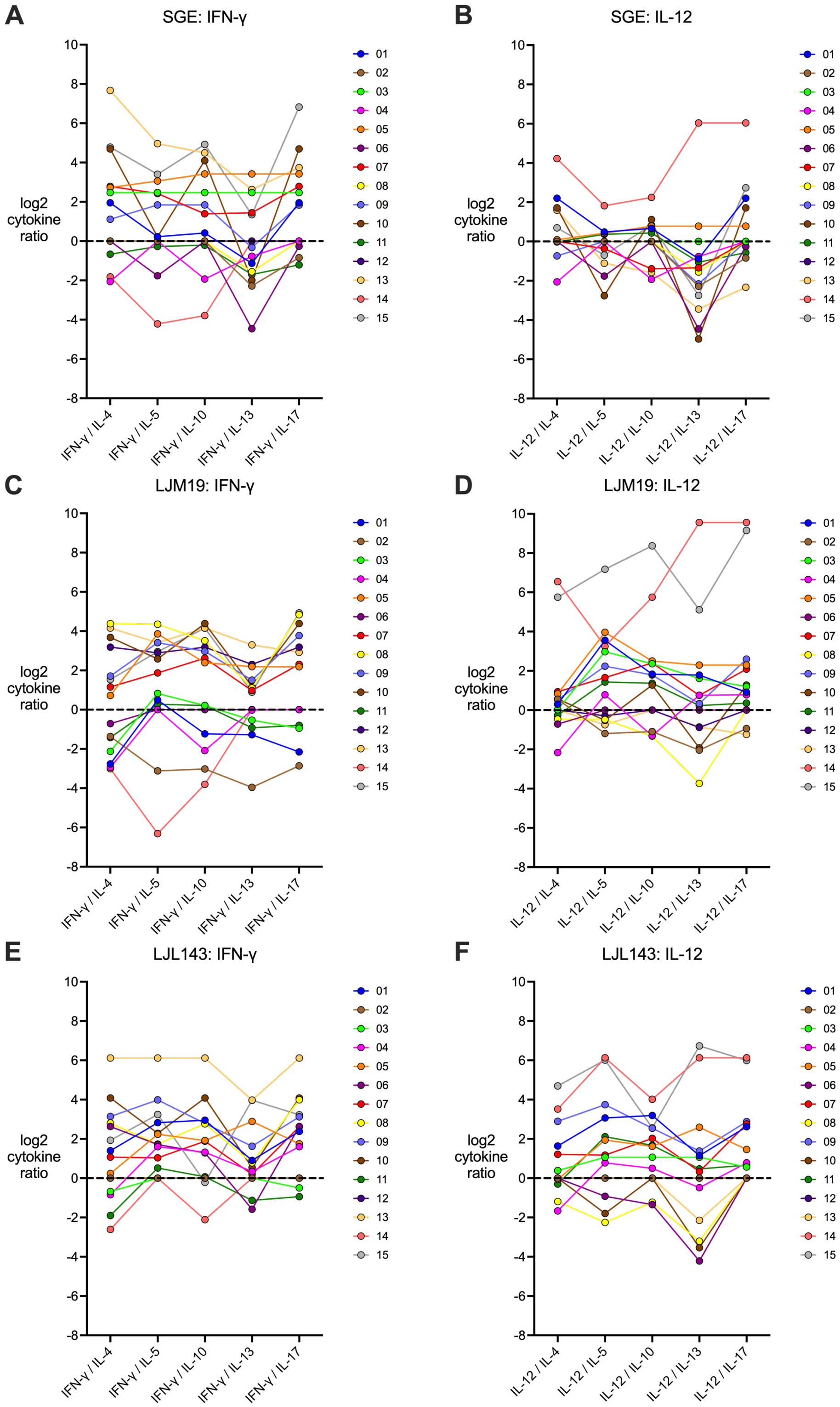

Supplemental Figure 3. Individual T_H cytokine response profiles to *Lu. longipalpis* SGE and salivary proteins. Re-plot of data from Figure 4 of cytokines produced by PBMCs from *Lu. longipalpis* exposed participants following stimulation with SGE (**A** and **B**), LJM19 (**C** and **D**), or LJL143 (**E** and **F**). Each color and line and their connecting points represent a single individual.

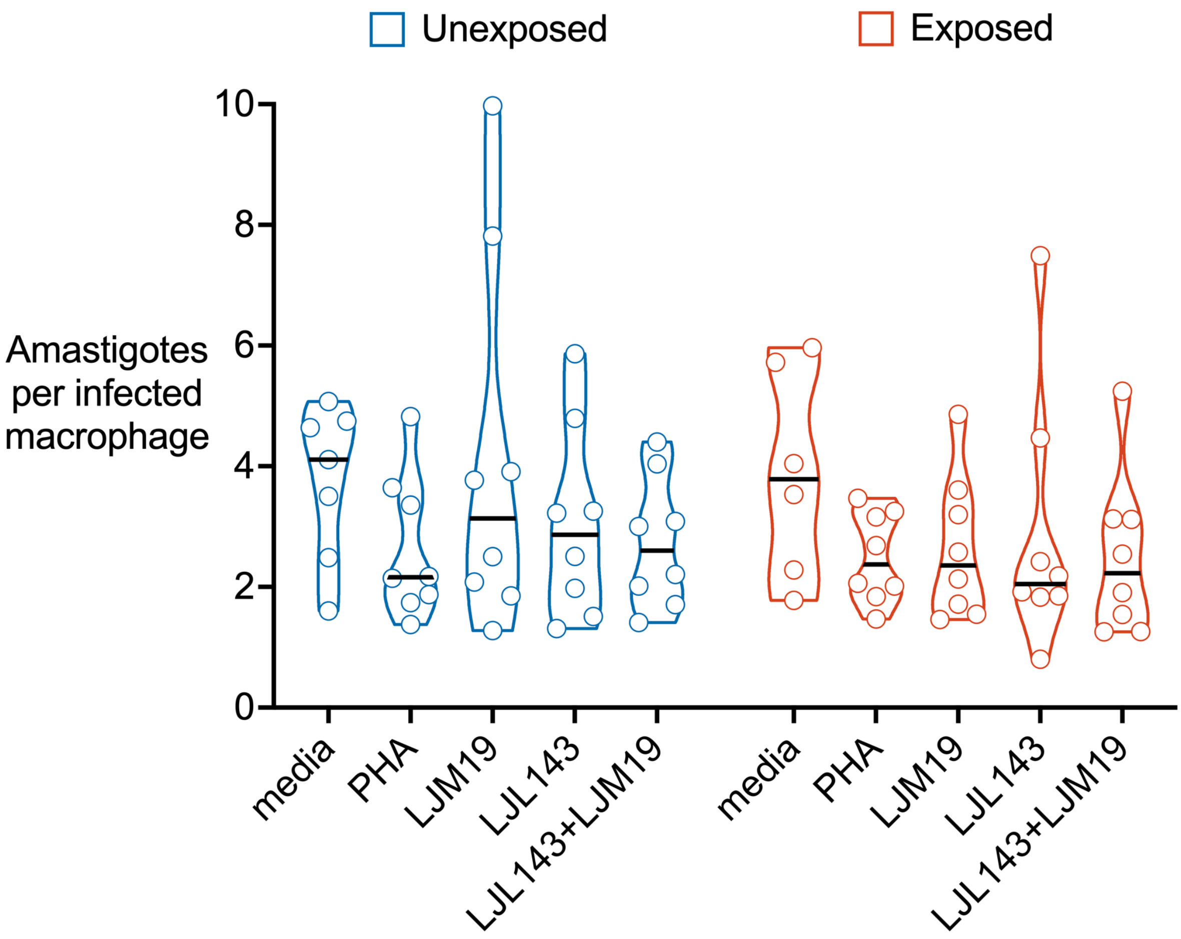

Supplemental Figure 4. Stimulation with LJM19 or LJL143 has no effect on the number of amastigotes per infected macrophage. Co-culture of PBMC-derived macrophages with stimulated PBMCs was performed as described in Figure 6. After 5 days of co-culture, the number of amastigotes per infected macrophage was quantified by manual counting of Giemsa-stained cells by light microscopy. Black bar indicates the median. Differences between treatment groups were analyzed via Kruskal-Wallis test. All p-values were > 0.05.

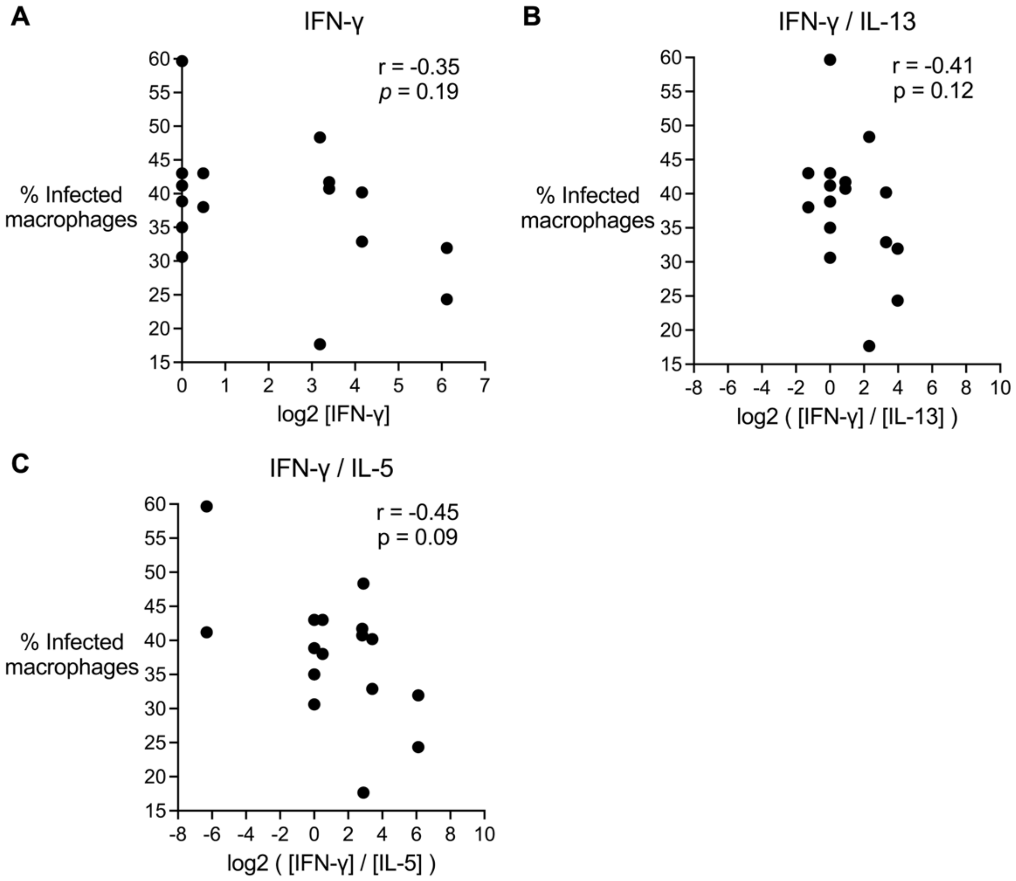

Supplemental Figure 5. Correlation of T_H cytokines with macrophage killing of *Leishmania* parasites. Spearman correlation between IFN-γ alone (**A**), IFN-γ/IL-13 ratio (**B**), or IFN-γ/IL-5 ratio (**C**) as calculated in Figure 4 and the percentage of infected macrophages for LJM19- and LJL143-treated samples.
